# Controlling the hydrogenolysis of silica-supported tungsten pentamethyl leads to a class of highly electron deficient partially alkylated metal hydrides†
†Electronic supplementary information (ESI) available: Complete experimental and quantum chemical procedures and characterization data for the prepared compounds. Additional information pertaining the calculation of NMR parameters. See DOI: 10.1039/c5sc03490f


**DOI:** 10.1039/c5sc03490f

**Published:** 2015-11-30

**Authors:** Niladri Maity, Samir Barman, Emmanuel Callens, Manoja K. Samantaray, Edy Abou-Hamad, Yury Minenkov, Valerio D'Elia, Adam S. Hoffman, Cory M. Widdifield, Luigi Cavallo, Bruce C. Gates, Jean-Marie Basset

**Affiliations:** a King Abdullah University of Science & Technology , KAUST Catalysis Center (KCC) , 23955-6900 Thuwal , Saudi Arabia . Email: jeanmarie.basset@kaust.edu.sa ; Email: emmanuel.callens@kaust.edu.sa; b Department of Materials Science and Engineering , Vidyasirimedhi Institute of Science and Technology , 21210 , Rayong , Thailand; c Department of Chemical Engineering and Materials Science , University of California at Davis , Davis , California 95616 , USA . Email: bcgates@ucdavis.edu; d Department of Chemistry , Durham University , Stockton Road , Durham DH1 3LE , UK

## Abstract

The well-defined single-site silica-supported tungsten complex [(≡Si–O–)W(Me)_5_], **1**, is an excellent precatalyst for alkane metathesis. The unique structure of **1** allows the synthesis of unprecedented tungsten hydrido methyl surface complexes *via* a controlled hydrogenolysis. Specifically, in the presence of molecular hydrogen, **1** is quickly transformed at –78 °C into a partially alkylated tungsten hydride, **4**, as characterized by ^1^H solid-state NMR and IR spectroscopies. Species **4**, upon warming to 150 °C, displays the highest catalytic activity for propane metathesis yet reported. DFT calculations using model systems support the formation of [(≡Si–O–)WH_3_(Me)_2_], as the predominant species at –78 °C following several elementary steps of hydrogen addition (by σ-bond metathesis or α-hydrogen transfer). Rearrangement of **4** occuring between –78 °C and room temperature leads to the formation of an unique methylidene tungsten hydride [(≡Si–O–)WH_3_(=CH_2_)], as determined by solid-state ^1^H and ^13^C NMR spectroscopies and supported by DFT. Thus for the first time, a coordination sphere that incorporates both carbene and hydride functionalities has been observed.

## Abstract 2

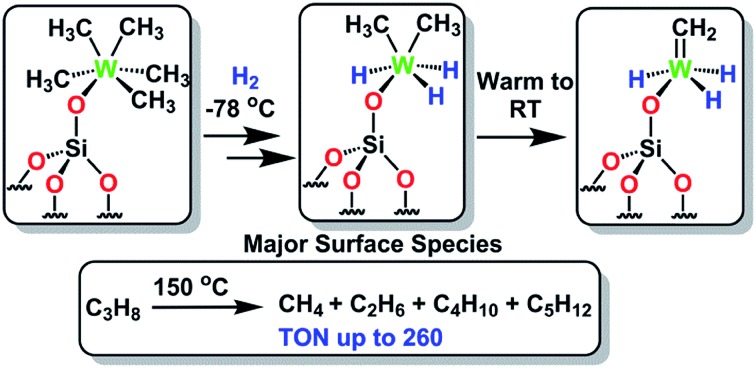
Accessing highly electron deficient partially alkylated tungsten hydrides on silica *via* controlled hydrogenolysis of surface organometallic complex (≡Si–O–)W(Me)_5_.

## Introduction

Since the discovery of [Cp_2_ReH] by Green and Wilkinson,^1^ transition metal hydrides have played an important role in organometallic chemistry. Their reactivity in many chemical transformations has been elucidated, including their involvement in the elementary steps of numerous industrial catalytic processes.^2^ Notably, transition metal hydrides have been extensively investigated for the activation of nearly inert C–H and C–C bonds in alkanes.^3^–^5^ Highly coordinated tungsten hydrides bearing phosphine ligands have been widely studied, and a few tungsten hydrides have been found to be catalytically active for the dehydrogenation of alkanes.^6^–^14^ The family of tungsten hydrides includes complexes grafted to oxide supports and synthesized by a surface organometallic chemistry (SOMC) strategy.^15^,^16^

This approach has led to the synthesis of numerous well-defined supported species that have allowed the elucidation of elementary reaction steps in heterogeneous catalytic processes.^17^ Compared to the phosphine-chelated tungsten hydrides used in solution, the surface organometallic species are generally highly reactive and electron-deficient. They were typically prepared and stabilized on supports including SiO_2_, Al_2_O_3_, and SiO_2_/Al_2_O_3_ by the hydrogenolysis of the grafted Schrock-type tungsten neopentyl/neopentylidyne complex [W(≡C–*t*-Bu)(CH_2_–*t*-Bu)_3_] (Bu = butyl) at 150 °C.^18^–^20^ These supported metal hydrides catalyse challenging and potentially valuable transformations including the metathesis of alkanes,^21^,^22^ the hydrogenolysis of saturated hydrocarbons,^23^,^24^ and the non-oxidative coupling of methane.^25^,^26^

Herein, we address alkane metathesis, defined as a reaction that transforms an alkane into its lower and higher homologues. In this regard, the immobilized tungsten hydrides serve as multifunctional precatalysts that engage in separate, successive elementary steps. These involve propagative species that are proposed to display both hydridic (for the C–H bond activation and olefin hydrogenation) and carbene functionalities (for the olefin metathesis steps).^27^ The SiO_2_/Al_2_O_3_ or Al_2_O_3_ supported tungsten hydrides were found to be more efficient precatalysts (with higher numbers of catalytic turnovers)^19^,^20^ than their silica-supported analogues due to the higher stability of the surface species on the former supports. The lower stability of the silica-supported tungsten hydrides was attributed either to the sintering of tungsten during hydrogenolysis of the precursor complex (at 150 °C) or to the transfer of hydrides from tungsten to silicon atoms of the support. Both effects lead to a decrease of the number of active sites available for catalysis.

We recently demonstrated that the silica-supported tungsten pentamethyl [(≡Si–O–)W(Me)_5_] (**1**) (Me = CH_3_) is a precatalyst for the metathesis of propane.^28^ As the initial C–H bond activation occurs by sigma bond metathesis,^3^ we postulated that this step would be more favourable by employing tungsten hydride precatalysts rather than tungsten methyl species. We also anticipated that hydrogenolysis of the W–Me moieties in **1** should take place more readily than for the analogous Schrock species,^29^ thus reducing the tendency of the W atoms towards sintering and H-transfer to the SiO_2_ surface.

**Scheme 1:**
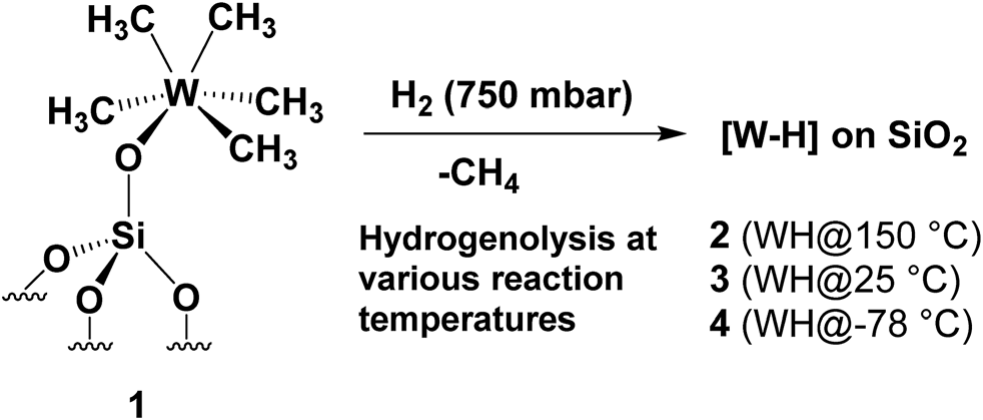
Synthesis of silica-supported tungsten hydrides [WH_*x*_/SiO_2_] (**2–4**) by treatment of **1** at various hydrogenolysis temperatures.

Herein, we demonstrate that the controlled hydrogenolysis of **1** at various temperatures (Scheme 1) leads to a variety of different surface complexes in terms of structure (as supported by DFT computations, infrared (IR) and solid-state NMR spectroscopies, and elemental microanalysis) and reactivity. Amongst them, unprecedented partially alkylated tungsten hydride species **4**, obtained from the hydrogenolysis of **1** at low temperature (<–70 °C), is the most active supported tungsten hydride species yet reported for single metal propane metathesis under batch conditions. The specific structural features of surface complex **4** are investigated here with the aid of solid-state NMR spectroscopy, DFT calculations and EXAFS spectroscopy (using **1** as a model substrate) leading to a deeper insight into the catalytically relevant intermediates generated upon its thermal rearrangement.

## Results and discussion

### IR spectroscopic characterization of the tungsten hydrides prepared from [(≡Si–O–)W(Me)_5_] (**1**) at three different hydrogenolysis temperatures

#### Hydrogenolysis at 150 °C

**Fig. 1:**
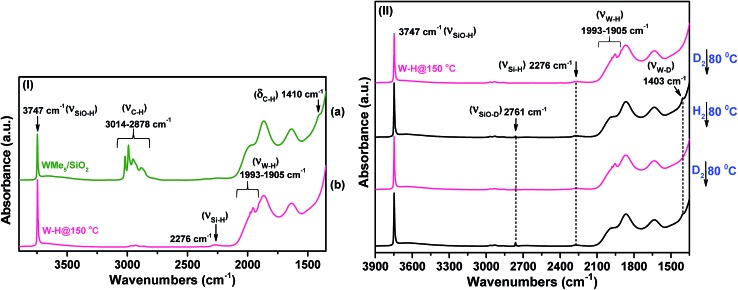
(I) IR spectra in the 3900–1400 cm^–1^ region recorded under vacuum (pressure <10^–5^ mbar) at 25 °C: (a) species **1** and (b) after hydrogenolysis at 150 °C (**2**). (II) IR spectra in the 3900–1400 cm^–1^ region of species **2** before and after the addition of D_2_ at 80 °C, followed by evacuation and then further addition of H_2_. This cycle of operations was repeated twice.

**Fig. 2:**
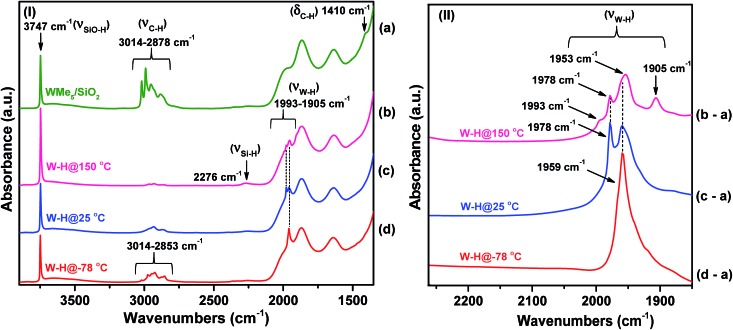
(I) IR spectra in the 3900–1400 cm^–1^ region recorded under vacuum (10^–5^ mbar) and 25 °C: (a) species **1** and (b) after hydrogenolysis of **1** at 150 °C (**2**); (c) after hydrogenolysis at 25 °C (**3**) and (d) after hydrogenolysis at –78 °C (**4**) (this spectrum was uniquely recorded at a temperature below –78 °C). (II) Difference IR spectra in the 2260–1850 cm^–1^ region obtained by subtraction of the spectra of hydrogenolysis products (**2–4**) and **1**, namely, (b-a) (representing the difference between spectrum b and spectrum a), (c-a), and (d-a).

**1** was initially treated with hydrogen gas (at 750 mbar and 150 °C for 15 h) to produce **2**. This hydrogenolysis treatment led to an immediate color change of the material from pale yellow to dark brown. Methane was evolved (detected by gas chromatography (GC)), corresponding to 4.9 ± 0.3 molecules per tungsten atom (matching within the error of theoretically expected C/W ratio of 5). The IR spectrum of **2** (Fig. 1(Ib)) clearly revealed the almost complete disappearance of the characteristic *ν*_(C–H)_ (3014–2878 cm^–1^) and *δ*_(C–H)_ (1410 cm^–1^) vibrational modes assigned to the CH_3_ groups of **1** (Fig. 1(Ia)), as an effect of hydrogenolysis and the appearance of more than one (weak) new bands in the 1993–1905 cm^–1^ range. The multiple broad bands at 1993, 1978, 1953, and 1905 cm^–1^ (Fig. 2(II(b-a))), fall in the frequency region expected for W(vi) hydride(s) (W–H) (*i.e.*, with tungsten in its highest oxidation state).^30^,^31^ Moreover, these values are close to those determined experimentally for distorted trigonal prismatic tungsten hexahydrides by Wang and Andrews.^30^,^31^ The stronger absorptions are assigned to the symmetric and antisymmetric (W–H) bond stretching vibrations. Contacting the sample of **2** with D_2_ at 80 °C led to the disappearance of all W–H vibrational absorptions and the emergence of a new band at 1403 cm^–1^ (Fig. 1(II)). This band is attributed to the W–D bond stretching and confirms the presence of a tungsten hydride species (on the basis of the harmonic approximation, the expected value of *ν*_W–D_ would be 1402 cm^–1^ for the *ν*_W–H_ band observed at 1978 cm^–1^). This deuterium/hydrogen exchange was found to be reversible, further corroborating the identifications of the silica-supported tungsten hydrides.

Moreover, the band at 2276 cm^–1^ in the IR spectrum of **2** supports the formation of ≡Si–H species possibly arising *via* hydride transfer from tungsten to the silica surface. This is a well-documented rearrangement whereby a hydride transfers to a neighbouring siloxane bridge, leading to the formation of supported bipodal metal hydride species at high temperatures.^32^ Taken together, the data strongly support the formation of W–H and Si–H bonds upon hydrogenolysis of **1** at 150 °C.

#### Hydrogenolysis at 25 °C

In agreement with our initial hypothesis, the hydrogenolysis of **1** could be carried out also at lower temperature. When **1** is allowed to react with H_2_ at 25 °C for 4 min, the IR spectrum of the resulting material **3** (Fig. 2(Ic) and (II(c-a))) clearly shows *ν*_(W–H)_ bands at 1978 and 1959 cm^–1^. Moreover, the absence of a band at 2276 cm^–1^ for *ν*_(Si–H)_, which is indirectly associated with a bipodal tungsten species,^32^ rules out the transfer of hydrides to the silica surface at room temperature and below.

#### Hydrogenolysis at –78 °C

This facile hydrogenolysis at 25 °C prompted us to investigate the same process at even lower temperatures (0, –20, and –78 °C).^29^ The tungsten hydrides obtained at 0 and at –20 °C showed similar multiple IR bands (with slight alterations in intensity when compared to **2** and **3**) corresponding to tungsten hydrides (Fig. S1†). In contrast, the material obtained at –78 °C (**4**), under otherwise identical hydrogenolysis conditions, showed mainly one intense band, at 1959 cm^–1^, which is assigned to a *ν*_(W–H)_ mode, along with residual *ν*_(C–H)_ bands in the 3014–2853 cm^–1^ region (Fig. 2(Id) and (II(d-a))). Therefore, the IR spectrum of **4** shows the coexistence of hydride functionality and residual or unreacted alkyl groups. **4** was observed to be stable at temperatures below –78 °C for more than 2 h (Fig. S2†).

Overall, the IR investigation shows that the controlled hydrogenolysis of **1** at different temperatures leads to unambiguously different surface species. Hydrogenolysis at 25 °C prevents the formation of the ≡Si–H species observed at 150 °C, which implies the lack of hydride transfer from tungsten to the support. On the other hand, residual alkyl species are still present on the surface after hydrogen treatment at –78 °C (**4**) along with a single strong *ν*_(W–H)_ band observed at 1959 cm^–1^. In subsequent sections, we provide discussion and support for the various possible surface species at each reaction temperature.

The presence of residual alkyl groups in **4** hints at a partial hydrogenolysis of **1** at low temperature resulting in the presence of unreacted methyl groups at the tungsten centres along with the newly introduced hydride functionalities. Partial hydrogenolysis was also confirmed by elemental microanalysis, which gave a C/W atomic ratio of 0.6 and 1.3 measured for **2** and **3**, respectively (Table S1†). This ratio further increases to 2.1 for **4** (measured at room temperature). Therefore, it is evident that decreasing the hydrogenolysis temperature leads to an increase in the amount of residual carbon. The microanalysis data are complemented by the CH_4_ gas quantification analysis after hydrogenolysis of **1**. At 150 °C, the loss of nearly 5 molecules of CH_4_ per tungsten atom was measured for **2**. This number decreased to 4.0 for **3** and to 1.7 for **4**. In agreement with our initial hypothesis, the silica-supported Schrock analogue^18^ of **1** did not show any trace of hydrogenolysis when reacted with H_2_ at –78 °C under identical conditions as per **1**. At 25 °C, partial hydrogenolysis of the silica-supported Schrock analogue was observed, but to a lesser extent than for **1** (see IR spectra in Fig. S3 in the ESI†).

### Solid-state NMR characterization of silica-supported tungsten hydride species **2**, **3** and **4**

**Fig. 3:**
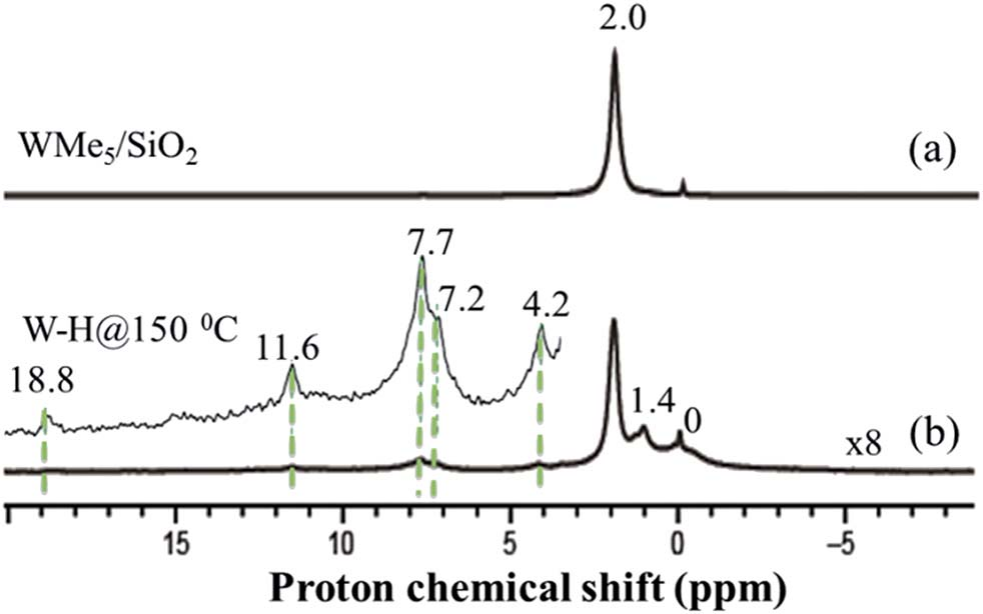
One-dimensional (1D) ^1^H solid-state NMR spectra recorded at room temperature for species **1** (a) and **2** (b) acquired at 500 MHz (*i.e.*, within a magnetic field, *B*_0_, of 11.7 T) with a 10 kHz MAS frequency for (a) and 12.5 kHz MAS frequency for (b), a repetition delay of 5 s, 32 scans for (a), and 128 scans for (b).

**Fig. 4:**
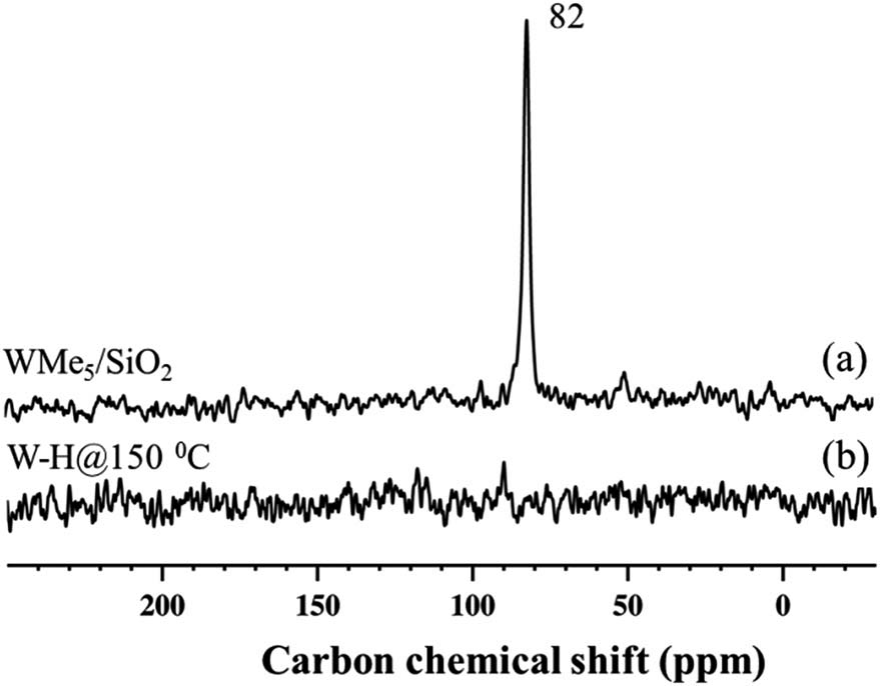
^13^C CP/MAS NMR spectra of ^13^C-enriched **1** recorded at room temperature (a) and its hydride analogue **2** (b) acquired at 125 MHz (*B*_0_ = 11.7 T) with a 10 kHz MAS frequency, 4 s repetition delay, and 2 ms contact time. 40 000 scans were recorded to produce (a) and 26 500 were recorded to yield (b).

Further spectroscopic analyses were also conducted with solid-state NMR. The ^1^H magic-angle spinning (MAS) solid-state NMR spectrum of **1** (Fig. 3(a)) displays one signal at 2.0 ppm, attributed to the five dynamically equivalent methyl groups,^28^ which overlaps with the Si–OH signal.^33^ However, the ^1^H MAS NMR spectrum of **2** (Fig. 3(b), scaled vertically by a factor of 8) indicates a large reduction in the intensity at 2.0 ppm and shows a very weak signal in the ^1^H–^1^H double-quantum/single-quantum (DQ/SQ) spectrum (Fig. S5†). These results indicate that most of the methyl groups were consumed during the reaction with H_2_ and that the signal at 2.0 ppm is mostly attributed to unreacted silanol (Si–OH).^33^ The ^1^H signal at 1.4 ppm, that shows a strong autocorrelation in the DQ/SQ NMR spectrum, is attributed to the W–Me moieties present in the newly formed surface species. The ^1^H resonance observed at 0.0 ppm is assigned to methyl groups attached to silicon atoms of the support surface, these can originate from methyl transfer from tungsten to the silica surface with a change of podality at the W-center (*vide infra*).^28^,^34^ The resonance at 4.2 ppm is assigned to the development of ≡Si–H through a bipodal structural transformation, which is consistent with the IR band observed at 2276 cm^–1^ (Fig. 1(Ib)).^32^ The signal at 7.2–7.7 ppm, showing autocorrelation in the DQ/SQ NMR spectrum (Fig. S5†), falls in the region of the W methylidene species (*vide infra*). However, in this case, it was not possible to confirm this attribution on the basis of the ^13^C spectrum of **2** (Fig. 4(b)).

Because tungsten hydrides are expected to be highly fluxional on the NMR time scale, and are known to be found within a wide ^1^H chemical shift range (*i.e.*, ranging from approximately +24 to –10 ppm),^7^,^35^,^36^ their detection and spectroscopic quantification can often be difficult.^19^,^37^ Indeed, an expanded view of the solid-state ^1^H NMR spectrum (inset of Fig. 3(b)) reveals several low-intensity resonances with positive chemical shifts. Consequently, we used density functional theory (DFT) calculations which included relativistic effects (both scalar and spin–orbit) using the zeroth-order regular approximation (ZORA) to predict the magnetic shielding and chemical shift values (*vide infra*) of a variety of tungsten hydrides on silica surface models. A complete specification of the protocol used here builds upon that established in an earlier study of **1**.^28^ In brief, we carefully benchmarked the ZORA DFT method using several well-characterized systems that can be found at the various points in the W–H shift range denoted above (see ESI, additional experimental discussion, Fig. S12–S15 and Tables S4 and S5†). The ^1^H chemical shifts for models of the W(vi) pentahydride species on silica have been calculated to occur in the range of 12–16 ppm (see the ESI†), depending upon the surface chosen to model the silica and also by considering the slightly different shifts for each of the 5 ^1^H sites in [(≡Si–O–)WH_5_].

Intriguingly, the variation in these chemical shifts appears to be nearly fully attributable to spin–orbit (*i.e.*, relativistic) effects, with variation in the other magnetic shielding mechanisms (*i.e.*, non-relativistic) being on the order of a few tenths of one ppm. This aspect is discussed more fully in the ESI, Section 6.† Hence, the weak high-frequency ^1^H signals at 11.6 and 18.8 ppm are assigned to surface WH_*x*_, consistent with the results of the DFT calculations. This is also in agreement with the presence of multiple IR bands of **2** assigned for *ν*_(W–H)_ in the range 1993–1905 cm^–1^ (Fig. 2(II(b-a))). The presence of multiple hydride bands in the FT-IR spectrum of **2**, and the likely formation of ≡Si–H and ≡Si–CH_3_ moieties observed respectively by FT-IR and ^1^H solid-state NMR demonstrate that hydrogenolysis at 150 °C leads to the formation of a variety of species with little control on structure and identity of the ensuing surface complexes.

The ^13^C cross-polarization (CP)/MAS NMR spectrum of **2** (Fig. 4(b)), prepared from 95% ^13^C enriched **1**, displayed no detectable signals in the +250 to –35 ppm range, even after >26 000 scans, consistent with the inference that most of the tungsten methyl groups had been hydrogenolysed. Solid-state ^1^H NMR spectra of **3** and **4** were compared with the spectrum obtained for **2** formed in the typical hydrogenolysis treatment at high temperatures. A set of proton resonances similar to that of **2** was detected in the spectrum of **3**, with the exception of the signal at 4.2 ppm corresponding to the ≡Si–H moieties of **2**. Furthermore, the solid-state ^13^C CP/MAS NMR spectrum of **3** shows signals at 42 and 47 ppm (Fig. S6†) attributable to unreacted tungsten methyl groups (W–*C*H_3_) which are left as a consequence of the partial hydrogenolysis of **1**.

Because species **4** was prepared at low temperature, we recorded the solid-state ^1^H NMR spectrum using a low-temperature probe, at 100 K. The spectrum displays multiple signals in the low-frequency range of 0–2 ppm, and at high frequency, weak signals, at 8.7 and 16.3 ppm. Assignments of these signals are presented after discussion of the various hydrogenolysis pathways starting with **1**.

### Evaluation of hydrogenolysis pathways by DFT calculations

To provide a better understanding on how the hydrogenolysis of the silica-supported tungsten complex [(≡Si–O–)W(Me)_5_], **1**, takes place, we proceeded with a DFT approach using a cluster model of the silica support. To ensure that our relatively small cluster model could be used to simulate the reactions on the silica surface, we validated it against periodic DFT and using slab silica models (see ESI† for details).^38^ For clarity in what follows, we use Roman numerals to denote all the various minimum-energy species. Transition states are described with the notation exemplified by [**I–II**]^‡^, referring to the transition state characterizing the transformation of **I** to **II**.

**Scheme 2:**
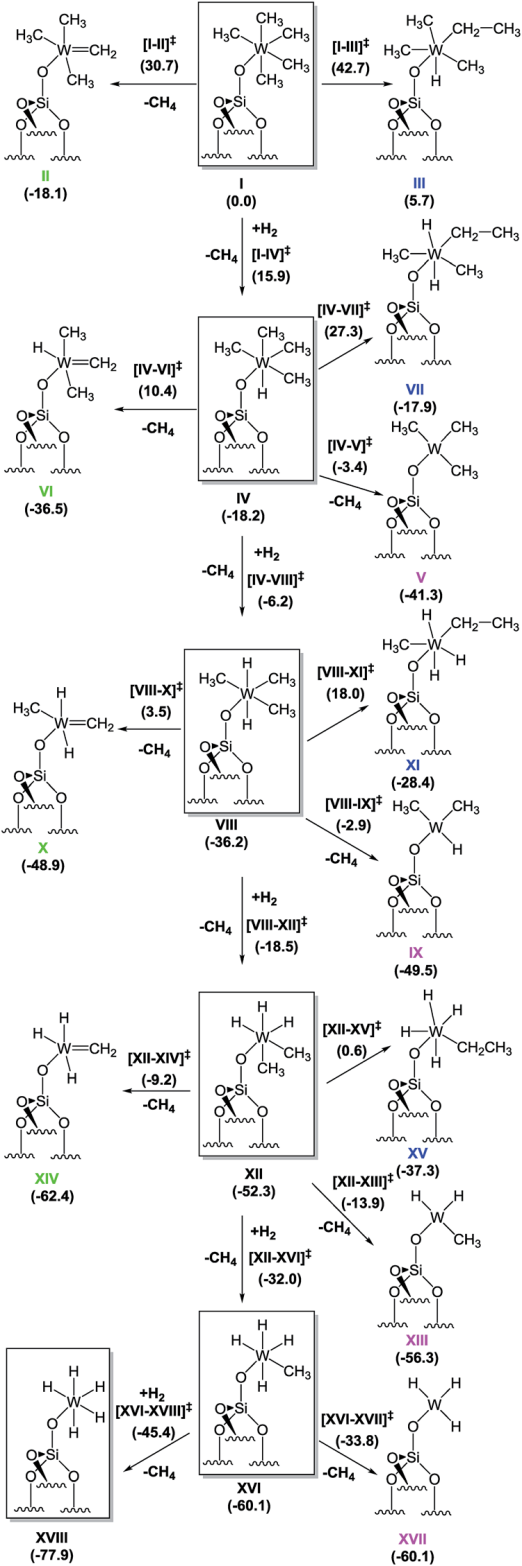
Proposed mechanism for hydrogenolysis of **1** (*i.e.*, **I**) into various tungsten hydrides; the values shown are free energies, in kcal mol^–1^. The energetically most favourable hydrogenolysis pathway is highlighted by the structures inside the boxes.

Starting from the silica-supported [(≡Si–O–)W(Me)_5_] precursor **1** depicted as **I** in Scheme 2, the calculations show that the reaction with molecular hydrogen leads to the formation of a dihydrogen bonded (*η*^2^–H_2_) heptacoordinated tungsten species, with predicted W–H distances of 1.84 Å, and the H–H bond elongated to 0.86 Å from 0.741 Å in gaseous H_2_.^39^ The H_2_ coordination is endergonic by 14.8 kcal mol^–1^. The hydrogenolysis of **I** occurs *via* transition state (TS) [**I–IV**]^‡^ and requires an activation energy of 15.9 kcal mol^–1^, making this process possible at room temperature. This step corresponds to a σ-bond metathesis event with the release of a CH_4_ molecule and formation of the tungsten monohydride [(≡Si–O–)WH(Me)_4_], **IV**, which is 18.2 kcal mol^–1^ lower in free energy than **I**.

In addition to this step, two other intramolecular transformations of **I** were explored. First, **I** can form tungsten methylidene **II***via* α-H elimination from a methyl group with the simultaneous release of one methane molecule. This process is also exergonic, with a Gibbs free energy release of 18.1 kcal mol^–1^, but the associated transition state [**I–II**]^‡^ is 30.7 kcal mol^–1^ higher in energy than **I**, and it is also 14.8 kcal mol^–1^ higher in energy than transition state [**I–IV**]^‡^, making α-H elimination highly disfavoured. Second, **I** can also undergo an intramolecular α-H transfer to the metal, with migration of another methyl group to form a tungsten ethyl hydride, **III**, *via* transition state [**I–III**]^‡^. This process is endergonic by 5.7 kcal mol^–1^, with an associated free energy barrier of 42.7 kcal mol^–1^, which excludes it from the reactivity scenario.

The subsequent transformation of **IV** to **VIII** was found to be even easier than the addition of the first hydrogen molecule to **I**, with a free energy barrier of only 12.0 kcal mol^–1^. This step is again exergonic, and the product [(≡Si–O–)WH_2_(Me)_3_], **VIII**, is more stable than **IV** by 18.0 kcal mol^–1^. Similar to the previous findings for **I**, the formation of a methylidene species (**VI**) or an ethyl hydride (**VII**) requires too high of an activation energy and these processes can thus be excluded from further consideration. However, in contrast to **I**, **IV** can undergo reductive elimination to form a triplet W(iv) species [(≡Si–O–)W(Me)_3_], **V**, with the release of one methane molecule. This process is favoured thermodynamically, with a free energy change of –23.1 kcal mol^–1^. The associated energy barrier estimated at the minimum energy crossing point (MECP) along the CH_3_–H(W) bond stretching mode turned out to be only 14.8 kcal mol^–1^. However, because direct hydrogen addition occurs *via* a free energy barrier lower by ∼3.0 kcal mol^–1^, the conversion of **IV** to **VIII** is the most likely route for further hydrogenolysis. The addition of the third, fourth, and fifth hydrogen atoms occurs *via* direct successive hydrogen additions. All other competing reactions again were found to require higher activation energies than the hydrogenation steps. Even reductive elimination, with formation of a W(iv) complex (which was found to have an activation energy of 14.8 kcal for **IV**), requires 33.3, 38.4, and 26.3 kcal mol^–1^ for complexes **VIII**, **XII**, and **XVI**, respectively. Eventually, the final step is the formation of a silica-supported tungsten pentahydride [(≡Si–O–)WH_5_], **XVIII**, which was found to be the most stable species of all those considered, with a Gibbs free energy of –77.9 kcal mol^–1^ relative to **I**.

The above characterization of transformations from **I** to **XVIII** suggests that hydrogenolysis of **I** is an exergonic process (Δ*G* ≈ –80 kcal mol^–1^), leading to the formation of silica-supported tungsten pentahydride as the thermodynamic product. The proposed mechanism is a cascade or sequence of hydrogen addition reactions forming the series of supported metallo hydrides ([(≡Si–O–)WH_*x*_(Me)_*y*_], *x* = 1–5, *y* = 4–0) along with the elimination of free methane molecules. The lowest barrier (+12 kcal mol^–1^) corresponds to the reaction with the second H_2_ molecule. The highest barrier was calculated for the addition of the fourth hydrogen molecule (during transformation of **XII** to **XVI**) and amounts to more than 20 kcal mol^–1^, suggesting that this step might be rate determining. On the basis of these calculations it is possible to suggest that the silica-supported [(≡Si–O–)WH_3_(Me)_2_] (**XII**) might represent the predominant surface species if **1** is hydrogenolysed to afford **4** at –78 °C. Other mechanistic pathways involving the reduction of W(vi) species have been found to be characterized by higher activation barriers.

### Evaluation of propane metathesis activities of catalysts formed from precatalysts **2**, **3**, and **4**

The catalytic performances of the various tungsten hydride species (**2–4**, Table 1) were examined for propane metathesis in a batch reactor. The results were compared with previously reported data for tungsten hydrides (entry 5). A relatively high turnover number (TON) (*i.e.*, 104) was obtained with **2** (prepared at 150 °C) (entry 1) compared with the tungsten hydrides prepared from a grafted neopentyl organometallic precursor.^19^ For species **3** (prepared at 25 °C), a further increase in activity for propane metathesis was observed (154, entry 2). This result could be attributed to a higher number of active tungsten hydride sites at this treatment temperature (25 °C), inasmuch as no apparent hydride transfer to the silica surface was observed by solid-state ^1^H NMR and IR spectroscopy (Fig. 2(Ic)). Notably, precatalyst **4**, prepared at an even lower temperature (–78 °C), gave the best activity for this transformation (entry 3, TON: 261). Significantly, the catalytic activity of species **4**, when it was allowed to warm to room temperature (Table 1, entry 6) before addition of propane, is comparable to that of **3**. This observation is in agreement with the structural rearrangement of **4** as discussed in the following paragraphs and demonstrates that the structure obtained at low temperature plays a key role in the catalytic activity. In general, the catalytic activity increases from **2** to **4**, and, therefore, with the number of unreacted methyl functionalities on the W-centre. Therefore, it appears that tuning the degree of hydrogenolysis by reducing the hydrogenolysis temperature is a crucial point in allowing the formation of highly active, partially alkylated hydrides. As shown below with the aid of DFT calculations, partially alkylated hydrides are required to generate key methylidene hydride intermediates. Moreover, it has to be taken into account that the amount of H_2_ generated in the reactor upon contact of the sample with propane is different for completely and partially alkylated species. The presence of H_2_ is related to the amount of alkenes, key intermediates in this alkane transformation.^40^ It has also been demonstrated that, under dynamic conditions, a competitive reaction, the hydrogenolysis of alkanes, occurs.^41^ Thus, the presence of partially hydrogenolysed species could have also an effect on the amount of H_2_ released in the reactor and favor alkane metathesis.

**Table 1:** Comparative propane metathesis activities expressed as TON and product selectivities of tungsten species on silica and their corresponding hydrides in a batch reactor at 150 °C for 5 days^*a*^

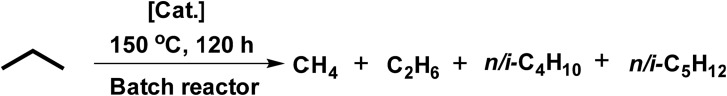							
Entry	Precatalyst	TON^*b*^ (% conversion)	Product selectivity^*c*^ (%)				Ref.
			Methane	Ethane	Butanes^*d*^	Pentanes^*d*^	
1	[WH@150 °C] (**2**)	104 (11.5)	4	50	28/15	2/1	This work
2	[WH@25 °C] (**3**)	154 (17)	3	56	30/5	4/2	This work
3	[WH@–78 °C] (**4**)^*e*^	261 (29)	3	58	27/5	4.5/2	This work
4	[WMe_5_/SiO_2_] (**1**)	127 (12)	2	54	33/4	6/1	28
5	[WH@150 °C]^*f*^	8 (1.2)	5.7	56	29/2.8	5.1/1.4	19
6	[WH@–78 °C] (**4**)^*g*^	166 (18.4)	3	55	29/5.4	5/2.4	This work

^*a*^In a typical procedure, propane gas (20.4 mmol) was introduced into the reactor at 25 °C.

^*b*^TON is expressed in (mol of propane transformed)/(mol of W) assuming that all the W atoms on the support are active.

^*c*^The selectivities are defined as the number of mols of product per mol of total product.

^*d*^Ratio of linear to branched alkanes.

^*e*^Propane (20.4 mmol) was introduced into the reactor at 77 K.

^*f*^Data taken from the following communication (*Angew. Chem., Int. Ed.*, 2005, **44**, 6755) whereby the tungsten hydride species were synthesized by hydrogenolysis of [(≡Si–O–)W(≡C–*t*-Bu)(CH_2_–*t*-Bu)_2_].

^*g*^Propane was introduced after allowing **4** to warm to room temperature.

### Structural investigation on species **4**

To have a better understanding of the activity enhancement in propane metathesis for pre-catalyst **4** and on how structural features are intertwined with catalytic activity we examined its thermal stability without substrate. First, an *in situ* IR variable temperature investigation of **4** was carried out. When **4**, prepared at –78 °C, was allowed to warm to room temperature, the initial W–H stretching band at 1959 cm^–1^ decreased with time, along with a decrease in intensity of the *ν*_C–H_ bands at 3014–2853 cm^–1^. As a result, a new, weaker band centred at 1978 cm^–1^ was observed (Fig. S4†). These data strongly support the evolution of a new tungsten hydride species at temperatures ranging from –78 °C to room temperature.

**Fig. 5:**
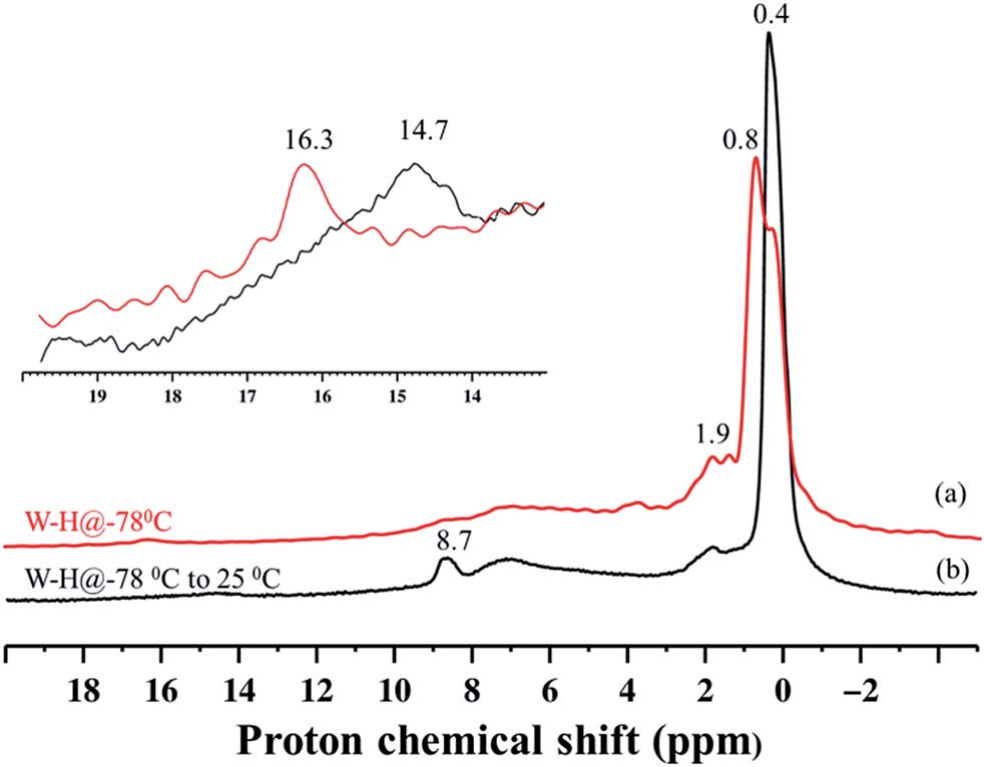
^1^H MAS solid-state NMR spectra of fresh **4** (a) and after warming to room temperature and holding for *ca.* 10 minutes (b). The spectra were acquired at 400 MHz (*B*_0_ = 9.4 T) with a 10 kHz MAS frequency, a repetition delay of 5 s, 8 scans, and at –173 °C.

**Fig. 6:**
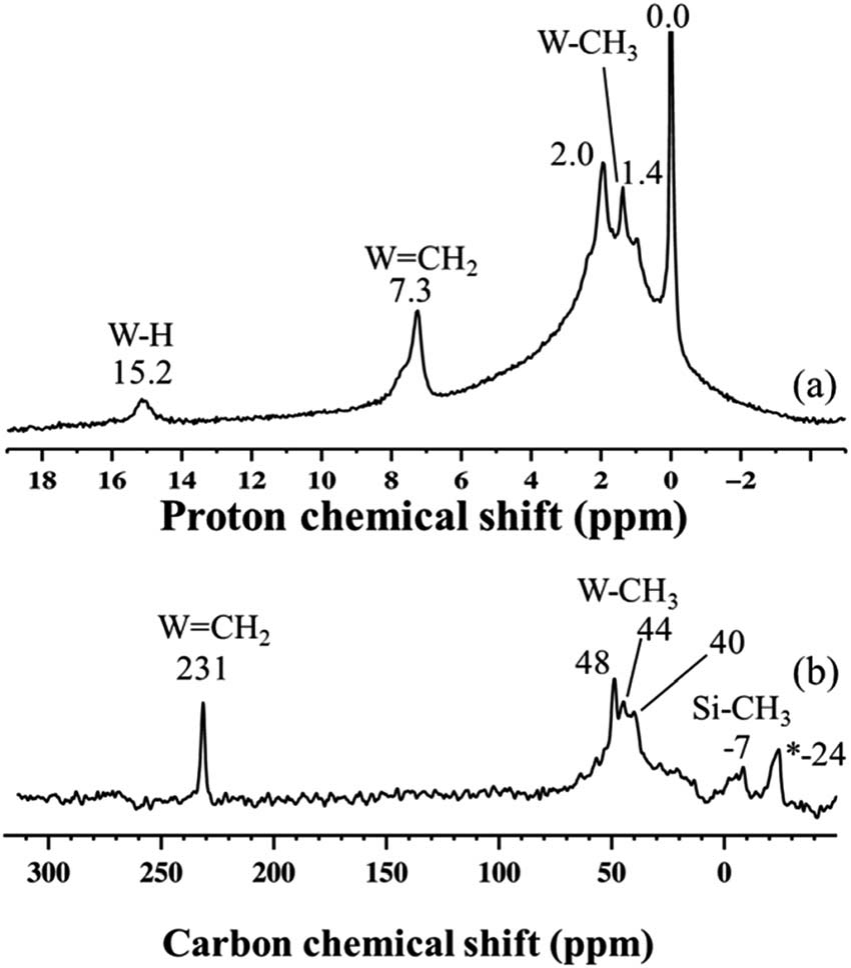
(a) One-dimensional (1D) ^1^H solid-state NMR spectrum of **4** recorded at room temperature; data acquired at 400 MHz with a 10 kHz MAS frequency, a repetition delay of 5 s, and 8 scans. (b) 1D ^13^C CP/MAS NMR spectrum of **4** recorded at room temperature; data acquired at 100 MHz with a 10 kHz MAS frequency, 4 s repetition delay, 2 ms contact time, and 20 000 scans. *The peak at –24 ppm is attributed to adsorbed ZnMe_2_, which was used in the preparation of the precursor complex WMe_6_.^45^

Second, this process was monitored by solid-state ^1^H and ^13^C CP/MAS NMR spectroscopies. Knowing the relatively poor thermal stability of **4**, we initially recorded the ^1^H NMR spectra using a pre-cooled system with the probe temperature set at 100 K (Fig. 5). In the solid-state proton NMR spectrum, ^1^H resonances in the 0.4 to 16.3 ppm region are evident (Fig. 5a). The ^1^H NMR signals at 0.4 and 0.8 ppm should correspond respectively to ≡Si–C*H*_3_ (ref. 42) and to the unreacted W–C*H*_3_ moieties in **4**. The signal at 1.9 ppm probably corresponds to unreacted silanols,^42^ whereas the weak signal at 16.3 ppm can be attributed to tungsten hydrides according to the calculations discussed above. The presence of ≡Si–CH_3_ or of a different kind of methyl group functionalities implies that the initial monopodal species **4** might have partially rearranged into a bipodal species under the experimental conditions by the transfer of a methyl group to the silica support.^28^,^43^ This transformation is not surprising as this behaviour has also been observed by EXAFS spectroscopy for the parent material [(≡Si–O–)W(Me)_5_] (*vide infra*). Allowing the rotor sample to warm to room temperature for 10 min (outside the NMR probe) and then reintroducing it into the cold NMR probe led to the complete disappearance of the ^1^H chemical shift at 0.8 ppm and to the increase of the signal at 0.4 ppm (Fig. 5b). Additionally, we observed the appearance of a signal at 8.7 ppm and a shift in the W hydride signal (from 16.3 ppm to 14.7 ppm, Fig. 5b). All the observations support the progressive rearrangement of **4** at temperatures higher than –78 °C. In a different experiment, the solid-state ^1^H and ^13^C NMR spectra of **4** were measured after allowing the sample to warm to room temperature (Fig. 6a) and with a longer acquisition time. We clearly observed an intense signal in the W hydride region at 15.2 ppm that allowed us to measure the average of spin–lattice relaxation *T*_1_ value for these protons of greater than 1500 ms (Fig. S7†) as we fitted the data with a stretched exponential decay.^44^ This value is characteristic of tungsten hydride species without the formation of tungsten dihydrogen complexes (*i.e.*, without W(*η*^2^–H_2_)).^13^

**Fig. 7:**
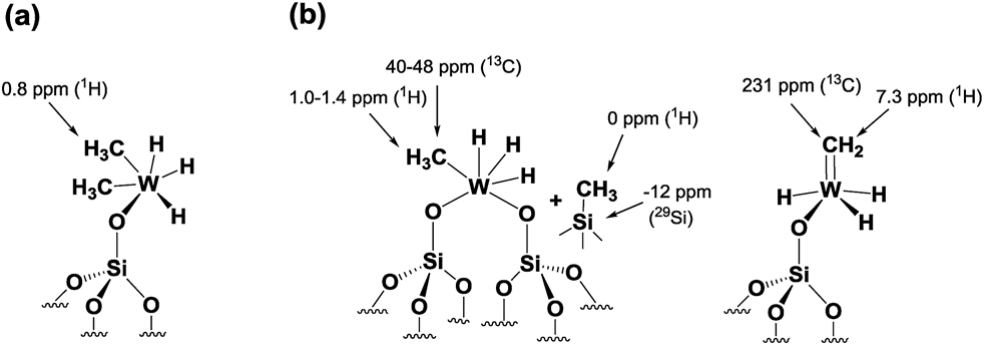
Plausible surface structures of tungsten hydride species in **4** (a) before and (b) after warming from –78 °C to RT.

The ^1^H NMR spectrum in Fig. 6a along with the corresponding ^1^H–^13^C HETCOR (Fig. S8†) spectrum shows a clear indication of static disorder that we tentatively attribute to the formation of bipodal species upon warming.^46^ Under these conditions we observed as well a signal at 7.3 ppm. The ^13^C CP/MAS NMR spectrum (Fig. 6b) displayed signals at 40–48 ppm, attributed, as before, to tungsten methyl (W–*C*H_3_) moieties, and a distinct signal at 231 ppm. The ^1^H peak at 7.3 ppm correlates only with the carbon resonance at 231 ppm in the 2D ^1^H–^13^C HETCOR NMR spectrum (Fig. S8†). These correlated signals are typical of a tungsten methylidene (W=C*H*_2_) moiety.^26^,^47^ This signal was not observed in the ^13^C solid-state NMR spectrum of species **3** generated directly at room temperature (Fig. S6†). No other correlation was observed between the ^13^C resonance signal at 231 ppm and the signals in the 30–50 ppm region, excluding the co-existence of a W-methyl within this W-methylidene species. Therefore, the structure proposed for this species is that of a methylidene hydride. Notwithstanding the apparent absence of spectroscopic correlation also between the W=CH_2_ and W–H signals, the formation of a methylidene hydride from partially alkylated W-hydrides is supported by DFT calculations (*vide infra*). The W-alkylidene species formed was found by ^1^H NMR spectroscopy to be stable at temperatures up to 150 °C, but it decomposed readily upon introduction of D_2_ gas at 80 °C. Moreover, at room temperature we observed a proton chemical shift at 0.0 ppm corresponding to ≡Si–C*H*_3_ moieties. This result is supported by the appearance of a peak at –12 ppm (attributed to surface ≡Si–CH_3_) in the ^29^Si CP/MAS NMR spectrum of **4** recorded at room temperature (Fig. S9†).^28^ Thus, on the basis of DFT calculations and a systematic solid-state NMR investigation we infer that warming of **4** to room temperature would lead to various monopodal and bipodal structures on the amorphous silica surface (Fig. 7).

To summarize our spectroscopic and DFT investigation, we represent species **4** as [(≡Si–O–)WH_3_(Me)_2_] (Fig. 7a); whereas the coexistence of alkyl and hydride moieties has been observed by IR (Fig. 2(Id)) and by solid state NMR spectroscopies (Fig. 5 and 6a), the average C/W ratio obtained by microanalysis (*ca.* 2 : 1, measured at room temperature) is in agreement with the inferred structure as a main surface component. Considering the low hydrogenolysis temperature of –78 °C, the structural proposal is also in agreement with the DFT calculations that show a relatively high energy barrier of 20.3 kcal mol^–1^ for the reaction of [(≡Si–O–)WH_3_(Me)_2_] with the fourth molecule of hydrogen (transition state **XII–XVI** of Scheme 2). Other surface species, that are consistent with the solid state NMR investigation reported above (and with the X-ray absorption spectroscopy results reported in the next paragraph), and are proposed to form on the surface from the evolution of **4** (when allowed to warm to room temperature), are depicted with the selected characteristic NMR signals assigned (Fig. 7b).

### X-ray absorption spectroscopy

The transformation of the well-defined monopodal species into a bipodal surface complex, accompanied by the transfer of methyl groups to the silicon atoms of the support, is invoked in the text as a major decomposition pathway for **4** when it is allowed to warm to room temperature. To provide further evidence for this chemical transformation, the evolution of **1** as a model compound was monitored by X-ray absorption spectroscopy (XAS) at room temperature with the sample in a stream of dry helium. As reported previously, microelemental analyses of **1** indicate that the tungsten pentamethyl moiety is anchored *via* only one isolated silanol group of the surface, maintaining its hexacoordination around the W center.

The structural parameters determined by the best fit of the data of the first W L_III_-edge EXAFS scan of **1** in flowing helium strongly support the inference of two absorber–backscatterer pairs, W–C and W–O, with internuclear distances consistent with typical sigma bonds. The W–C and W–O coordination numbers were found to be 4.9 and 1.0, respectively. Within the expected error (±20%), these values are consistent with the initial supported species **1** being monopodal on the surface. Furthermore, there was no detectable W–W contribution in the fit, indicating that, within error, the tungsten species remained mononuclear.

When **1** was present in the flow-through cell in the presence of helium (flow rate: 1.0 mL min^–1^), with continuous exposure to the X-ray beam, changes in the X-ray absorption near edge structure (XANES) region of the X-ray absorption spectrum show that the supported tungsten species was transformed.

**Fig. 8:**
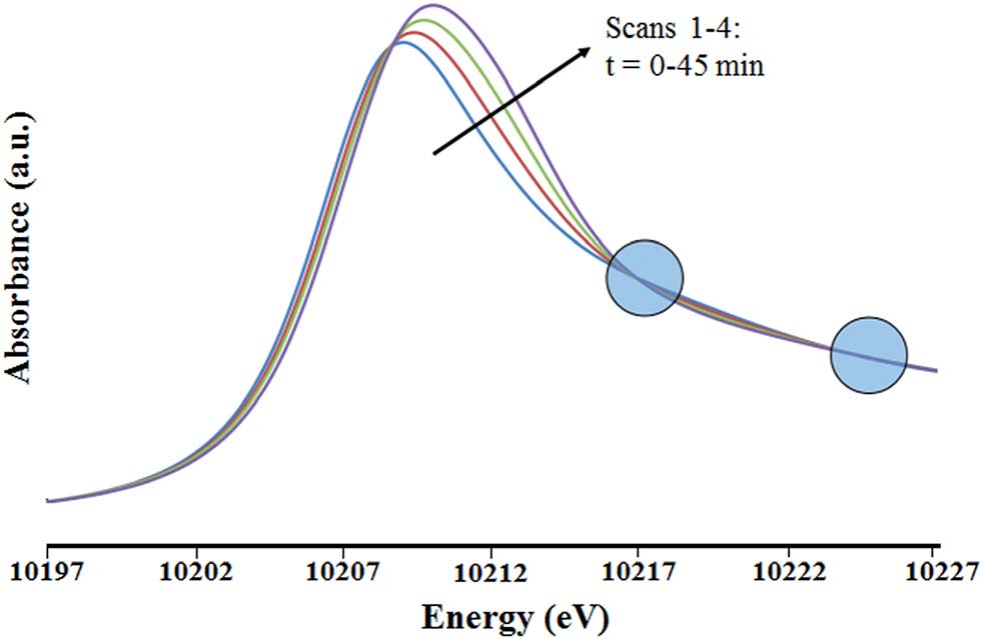
XANES region of the X-ray absorption spectra at the W L_III_ edge characterizing the sample initially present as the silica-supported tungsten complex [(≡Si–O–)W(Me)_5_] as it underwent changes in the presence of flowing helium at 298 K and 1 bar. Isosbestic points (circled) indicate a nearly stoichiometric conversion of the initial species. The blue, red, green and purple spectra represent the first to fourth scans, respectively.

The XANES region (Fig. 8) shows the edge energy increasing slightly, from 10 208.9 to 10 209.9 eV. This change suggests a structural rearrangement and/or an increase in the tungsten oxidation state during the treatment. Isosbestic points in the XANES spectra (Fig. 8) indicate a stoichiometric change from the initial species. Because the changes occurred slowly, the first EXAFS spectrum of the sample in flowing helium (which was recorded during the first 15 min of exposure to the X-ray beam) is considered to be essentially indicative of the initial supported tungsten complex. The two EXAFS spectra recorded after the first one show that the tungsten coordination sphere was changing during the experiment. The second and third scans were only slightly different from the first, but the fourth was sufficiently different to justify a detailed analysis.

This fourth EXAFS spectrum was recorded starting 45 min after the initial exposure of the sample to the X-ray beam. The data were of lower quality than those of the first scan, so that the fit parameters were determined with less confidence. Several plausible models were tested in the fitting of the data characterizing the first and fourth spectra. The fits according to the recommended models (see Tables 2, S2, S3 and Fig. S10, S11 in ESI†) are characterized by physically realistic values of the fitting parameters. Attempts were made to obtain satisfactory fits with alternative structural models. However, all the alternative models converged to the best fit models previously stated. Details of the data and analyses, including fits according to structural models that were rejected, are shown in the ESI.† The relatively large Δ*E*_0_ value determined in the best fit of the W–C shell of species **1** after 45 min in contact with flowing helium (Table 2) is attributed to the limited number of shells permitted in a statistically justified fit (indicated by the overall data quality and the Nyquist theorem;^48^ details are given below).

**Table 2:** EXAFS fit parameters^*a*^ characterizing the tungsten species supported on SiO_2_ at the W L_III_ edge^*b*^

Species	Shell	*N*	*R* [Å]	10^3^ × Δ*σ*^2^ [Å^2^]	Δ*E*_0_ [eV]
**1**	W–O_support_	1.03	1.81	3.70	–3.73
	W–C	4.91	2.02	8.56	4.68
**1** after 45 min in flowing helium (1 bar) at 298 K	W–O_support_	1.56	1.82	9.78	–4.23
	W–C	3.77	2.00	12.0	14.6

^*a*^Notation: *N*, coordination number; *R*, distance between absorber and backscatterer atoms; Δ*σ*^2^, disorder term; Δ*E*_0_, inner potential correction. Error bounds characterizing the structural parameters are estimated to be as follows: *N*, ±20%; *R*, ±0.02 Å; Δ*σ*^2^, ±20%; and Δ*E*_0_, ±20%.

^*b*^Details pertaining to the best fits can be found in the ESI (Tables S2, S3 and Fig. S10 and S11).

A comparison of the data from the first and fourth scans shows that, within error, the supported tungsten complexes lost methyl groups during the treatment in helium but remained six-coordinate, as a new W–support-oxygen bond formed. Thus, the EXAFS data are in agreement with the rearrangement of **1** with a proximate Si–O–Si bridge from monopodal to bipodal at ambient temperature (Scheme S1†).

### DFT calculation to support the formation of tungsten methylidene (W=CH_2_) moiety by warming **4** from –78 °C to room temperature

**Scheme 3:**
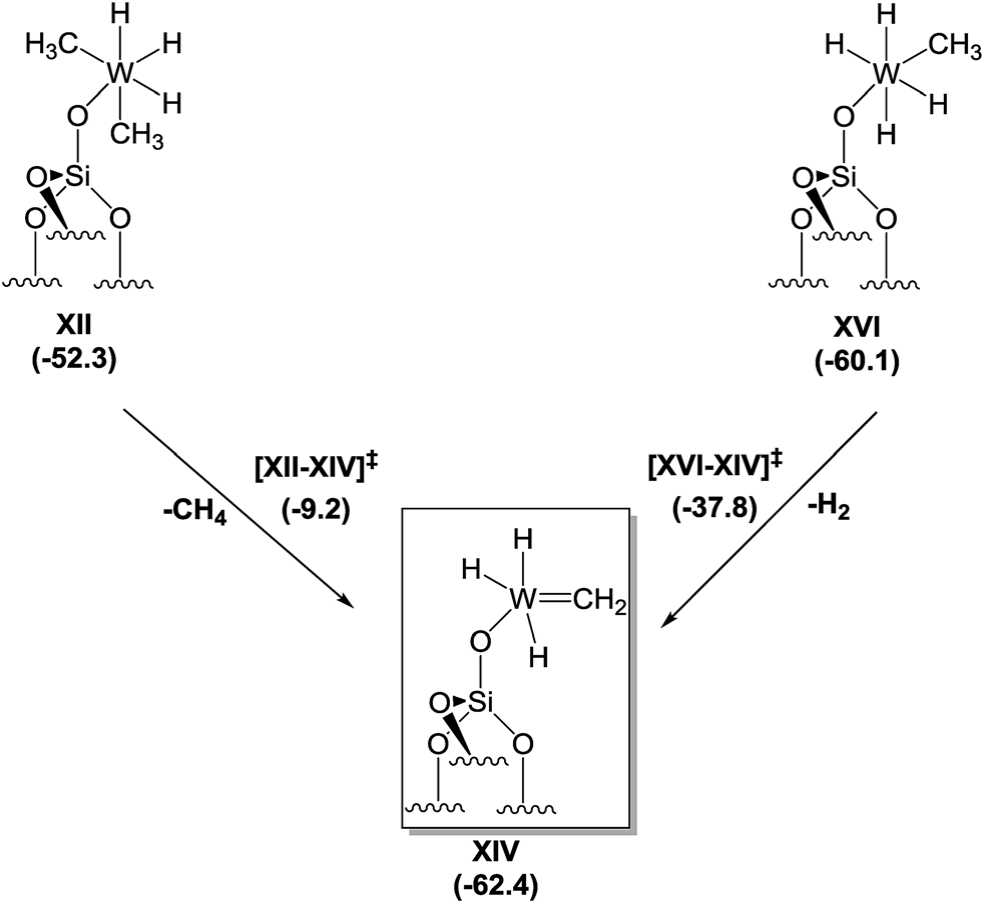
Proposed mechanism for the formation of tungsten methylidene hydrides from **XII** or **XVI** based on DFT calculations.

Motivated by the structural rearrangements in the structure of **4**, inferred from the solid-state ^1^H and ^13^C NMR data, we investigated the formation of the supported tungsten methylidene species (*i.e.*, **XIV**, Scheme 3) by DFT, envisaging two possible routes for its formation. The first corresponds to α-H elimination from **XII** with release of a CH_4_ molecule to produce a carbene, **XIV**, whereas the second corresponds to an α-H transfer from **XVI**. Focusing on the former, we found that the conversion of **XII** to **XIV** is an exergonic process (–10.1 kcal mol^–1^), but the associated activation barrier for the direct conversion *via* transition state [**XII–XIV**] is quite high (43.1 kcal mol^–1^) to be accessible under the reaction conditions used. In contrast, the conversion of **XVI** to **XIV** is exergonic by only 2.3 kcal mol^–1^, and the associated free energy barrier for the direct conversion *via* transition state [**XVI–XIV**] and the release of a H_2_ molecule amounts to just 22.3 kcal mol^–1^. This barrier can be overcome at room temperature, suggesting that the dominant route for the formation of **XIV** starts from **XVI**.

Notwithstanding the result that **XII** is the predominant species at low temperatures according to our spectroscopic investigation and DFT calculations, species **XVI** can be formed by a less than stoichiometric conversion in the hydrogenolysis of **XII** to **XVI**, which we calculated (Scheme 2) to be a facile process.

Thus, the DFT calculations are consistent with the formation of an unprecedented silica-supported tungsten methylidene hydride species (Scheme 3) following hydrogenolysis of **1** at –78 °C and warming to room temperature.

## Conclusion

The controlled hydrogenolysis of silica-supported tungsten pentamethyl [(≡Si–O–)W(Me)_5_] (**1**) has been investigated at various temperatures. The hydride products have been characterized by means of elemental microanalysis, IR and solid-state NMR spectroscopies revealing that hydrogenolysis is temperature dependent and leads to different structural features. The results demonstrate that the hydrogenolysis of [(≡Si–O–)W(Me)_5_] at –78 °C leads to an unprecedented silica-supported partially methylated tungsten hydride species. On the basis of DFT calculations, we propose that [(≡Si–O–)WH_3_(Me)_2_] is the predominant surface species at this temperature. The thus-synthesized tungsten hydride represents the precursor of the most active catalyst for propane metathesis yet reported. The thermal rearrangement of this partially methylated species, when allowed to warm to room temperature, has been observed to proceed *via* divergent pathways. This change involves methyl transfer to the silica surface, evidenced by the presence of ≡Si–CH_3_ species indicated by solid-state NMR spectroscopy, transforming the initial monopodal surface complex **4** into a bipodal species. Such a change in podality was indicated by EXAFS spectra determined with **1** as a model substrate. Furthermore, the formation of a tungsten alkylidene (methylidene) hydride intermediate, which has always been considered in alkane metathesis but never been observed, has been proposed on the basis of solid-state NMR spectroscopy and DFT calculations.

## Supplementary Material

Supplementary information

## References

[cit1] Green M. L. H., Pratt L., Wilkinson G. (1958). J. Chem. Soc..

[cit2] McGrady G. S., Guilera G. (2003). Chem. Soc. Rev..

[cit3] Labinger J. A., Bercaw J. E. (2002). Nature.

[cit4] Watson P. L., Parshall G. W. (1985). Acc. Chem. Res..

[cit5] Parshall G. W. (1975). Acc. Chem. Res..

[cit6] Moss J. R., Shaw B. L. (1968). Chem. Commun..

[cit7] Gregson D., Howard J. A. K., Nicholls J. N., Spencer J. L., Turner D. G. (1980). J. Chem. Soc., Chem. Commun..

[cit8] Hursthouse M. B., Lyons D., Thorntonpett M., Wilkinson G. (1983). J. Chem. Soc., Chem. Commun..

[cit9] Crabtree R. H., Hlatky G. G. (1982). J. Organomet. Chem..

[cit10] Hlatky G. G., Crabtree R. H. (1985). Coord. Chem. Rev..

[cit11] Green M. L. H., Parkin G. (1985). J. Chem. Soc., Chem. Commun..

[cit12] Green M. L. H., Parkin G. (1987). J. Chem. Soc., Dalton Trans..

[cit13] Sattler A., Zuzek A. A., Parkin G. (2014). Inorg. Chim. Acta.

[cit14] Crabtree R. H., Felkin H., Morris G. E., King T. J., Richards J. A. (1976). J. Organomet. Chem..

[cit15] Copéret C., Chabanas M., Saint-Arroman R. P., Basset J. M. (2003). Angew. Chem., Int. Ed..

[cit16] Modern Surface Organometallic Chemistry, ed. J. M. Basset, R. Psaro, D. Roberto and R. Ugo, Wiley-VCH Verlag GmbH & Co. KGaA, Weinheim, 2009.

[cit17] Serna P., Gates B. C. (2014). Acc. Chem. Res..

[cit18] Le Roux E., Taoufik M., Chabanas M., Alcor D., Baudouin A., Copéret C., Thivolle-Cazat J., Basset J. M., Lesage A., Hediger S., Emsley L. (2005). Organometallics.

[cit19] Le Roux E., Taoufik M., Copéret C., de Mallmann A., Thivolle-Cazat J., Basset J. M., Maunders B. M., Sunley G. J. (2005). Angew. Chem., Int. Ed..

[cit20] Le Roux E., Taoufik M., Baudouin A., Copéret C., Thivolle-Cazat J., Basset J. M., Maunders B. M., Sunley G. J. (2007). Adv. Synth. Catal..

[cit21] Vidal V., Theolier A., Thivolle-Cazat J., Basset J. M. (1997). Science.

[cit22] Basset J. M., Copéret C., Soulivong D., Taoufik M., Thivolle-Cazat J. (2010). Acc. Chem. Res..

[cit23] Norsic S., Larabi C., Delgado M., Garron A., de Mallmann A., Santini C., Szeto K. C., Basset J. M., Taoufik M. (2012). Catal. Sci. Technol..

[cit24] Dufaud V. R., Basset J. M. (1998). Angew. Chem., Int. Ed..

[cit25] Soulivong D., Norsic S., Taoufik M., Copéret C., Thivolle-Cazat J., Chakka S., Basset J. M. (2008). J. Am. Chem. Soc..

[cit26] Szeto K. C., Norsic S., Hardou L., Le Roux E., Chakka S., Thivolle-Cazat J., Baudouin A., Papaioannou C., Basset J. M., Taoufik M. (2010). Chem. Commun..

[cit27] Basset J. M., Copéret C., Lefort L., Maunders B. M., Maury O., Le Roux E., Saggio G., Soignier S., Soulivong D., Sunley G. J., Taoufik M., Thivolle-Cazat J. (2005). J. Am. Chem. Soc..

[cit28] Samantaray M. K., Callens E., Abou-Hamad E., Rossini A. J., Widdifield C. M., Dey R., Emsley L., Basset J. M. (2014). J. Am. Chem. Soc..

[cit29] Shortland A. J., Wilkinson G. (1973). J. Chem. Soc., Dalton Trans..

[cit30] Wang X. F., Andrews L. (2002). J. Am. Chem. Soc..

[cit31] Wang X. F., Andrews L. (2002). J. Phys. Chem. A.

[cit32] Rataboul F., Baudouin A., Thieuleux C., Veyre L., Copéret C., Thivolle-Cazat J., Basset J. M., Lesage A., Emsley L. (2004). J. Am. Chem. Soc..

[cit33] Legrand A. P., Hommel H., Tuel A., Vidal A., Balard H., Papirer E., Levitz P., Czernichowski M., Erre R., Vandamme H., Gallas J. P., Hemidy J. F., Lavalley J. C., Barres O., Burneau A., Grillet Y. (1990). Adv. Colloid Interface Sci..

[cit34] Chen Y., Callens E., Abou-Hamad E., Merle N., White A. J. P., Taoufik M., Copéret C., Le Roux E., Basset J. M. (2012). Angew. Chem., Int. Ed..

[cit35] Schrock R. R., Shih K. Y., Dobbs D. A., Davis W. M. (1995). J. Am. Chem. Soc..

[cit36] Dobbs D. A., Schrock R. R., Davis W. M. (1997). Inorg. Chim. Acta.

[cit37] Joubert J., Delbecq F., Sautet P., Le Roux E., Taoufik M., Thieuleux C., Blanc F., Copéret C., Thivolle-Cazat J., Basset J. M. (2006). J. Am. Chem. Soc..

[cit38] The relative electronic energies obtained for other intermediates are also similar between those two models; the difference is within 5.0 kcal/mol. Because such a difference is acceptable and equal to what one would expect from simply changing of the DFT functional and basis set, and also due to much larger CPU resources/time required for periodic simulations, it was decided to use the cluster model in the present study. For recent computational applications of the same cluster model see also: D'Elia V. Dong H. Rossini A. J. Widdifield C. M. Vummaleti S. V. Minenkov Y. Poater A. Abou-Hamad E. Pelletier J. D. Cavallo L. Emsley L. Basset J. M., J. Am. Chem. Soc., 2015, 137 , 7728 . 25950495 10.1021/jacs.5b02872

[cit39] Craw J. S., Bacskay G. B., Hush N. S. (1993). Inorg. Chem..

[cit40] Riache N., Callens E., Samantaray M. K., Kharbatia N. M., Atiqullah M., Basset J. M. (2014). Chem.–Eur. J..

[cit41] Soignier S., Saggio G., Taoufik M., Basset J. M., Thivolle-Cazat J. (2014). Catal. Sci. Technol..

[cit42] Chen Y., Zheng B., Abou-Hamad E., Hamieh A., Hamzaoui B., Huang K. W., Basset J. M. (2014). Chem. Commun..

[cit43] Ahn H., Marks T. J. (2002). J. Am. Chem. Soc..

[cit44] Lapadula G., Trummer D., Conley M. P., Steinmann M., Ran Y.-F., Brasselet S., Guyot Y., Maury O., Decurtins S., Liu S.-X., Copéret C. (2015). Chem. Mater..

[cit45] Luzgin M. V., Rogov V. A., Arzumanov S. S., Toktarev A. V., Stepanov A. G., Parmon V. N. (2008). Angew. Chem., Int. Ed..

[cit46] Badia A., Demers L., Dickinson L., Morin F. G., Lennox R. B., Reven L. (1997). J. Am. Chem. Soc..

[cit47] Blanc F., Berthoud R., Copéret C., Lesage A., Emsley L., Singh R., Kreickmann T., Schrock R. R. (2008). Proc. Natl. Acad. Sci. U. S. A..

[cit48] Lytle F. W., Sayers D. E., Stern E. A. (1989). Phys. B.

