# Supplementary material for: Controlling the hydrogenolysis of silica-supported tungsten pentamethyl leads to a class of highly electron deficient partially alkylated metal hydrides
Source: Chem Sci. 2015 Nov 30;7(2):1558–68. doi: 10.1039/c5sc03490f (PMC5964938; doi:10.1039/c5sc03490f)
Supplement: Supplementary file 1 [file SC-007-C5SC03490F-s001.pdf]

Electronic Supplementary Material (ESI) for Chemical Science.
This journal is © The Royal Society of Chemistry 2015

Electronic Supplementary Information

Controlling the Hydrogenolysis of Silica-Supported Tungsten Pentamethyl Leads to a Class of Highly Electron Deficient Partially Alkylated Metal Hydrides

Niladri Maity,[a] Samir Barman,[a] Emmanuel Callens,[*a] Manoja K. Samantaray,[a] Edy Abou-Hamad,[a] Yury Minenkov,[a] Valerio D′Elia,[a,b] Adam Hoffman,[c] Cory M. Widdifield,[d] Luigi Cavallo,[a] Bruce C. Gates[*b] and Jean-Marie Basset[*a]

[a]King Abdullah University of Science & Technology, KAUST Catalysis Center (KCC), 23955-6900 Thuwal, Saudi Arabia.
[b] Department of Materials Science and Engineering, Vidyasirimedhi Institute of Science and Technology, Thailand.
[c]Department of Chemical Engineering and Materials Science, University of California at Davis, Davis, California 95616, United States.
[d]Department of Chemistry, Durham University, Stockton Road, Durham DH1 3LE, United Kingdom.

Table of contents

1. General procedures……………………………………………………….......S2

2. Microelemental Analysis and GC analysis of 1-4…………………………………S7

3. IR spectra……………………………………………………………………………S8

4. Solid-state NMR investigation ……………………………………………………..S10

5. EXAFS investigation of 1…………………………………………………….…..S14

6. Additional details pertaining to the computation of hydrogen magnetic shielding and ^{1}H chemical shift values…………………………………………………………………S19

7. Computational models, details and coordinates of all species……………………S25

8. Supporting references………………………………………………………………S131

1. General procedures

All sample handling steps were performed in a glovebox and with standard Schlenk techniques under argon atmosphere. The syntheses and treatments of the surface organometallic complexes were carried out by using high-vacuum lines (< 10^{-5} mbar) and glovebox techniques. Chemicals were purchased from commercial sources and used as received. Solvents were dried, distilled, and degassed through freeze-pump-thaw cycles before use. Silica-supported (SiO_{2-700}; the subscript 700 refers to the treatment temperature in degrees C) precursor **1** [(≡Si–O–)W(Me)$_5$] and its ^{13}C-enriched analogue [(≡Si–O–)W(Me*)$_5$] were synthesized according to a literature report.[1] Gaseous hydrogen and propane were dried and deoxygenated before use by passage through a mixture of freshly regenerated molecular sieves (3Å) and R$\bar{3}$-15 catalysts (BASF). Elemental analyses were performed at Mikroanalytisches Labor Pascher (Germany). Gas-phase analysis of alkanes was performed with an Agilent 6850 gas chromatograph with a split injector coupled with a flame ionization detector. An HP-PLOT Al_2O_3 KCl 30 m × 0.53 mm, 20.00 mm capillary column coated with a stationary phase of aluminum oxide deactivated with KCl was used with helium as the carrier gas at 32.1 kPa. Each analysis was carried out under the same conditions, with a flow rate of 1.5 mL/min and an isotherm at 80 °C.

Typical procedure for synthesis of supported WH_x/SiO_2 hydrides (2, 3, and 4).

(a) **2**: A sample of **1** (100 mg) and dry H_2 (750 mbar) were added into a batch reactor of known volume (485 mL). The reaction mixture was heated to 150 °C at a rate of 5°C/min, with continued heating for 15 h. An aliquot of the gas mixture was released and analyzed by gas chromatography. Subsequently, the reactor was evacuated under vacuum (10^{-5} mbar) to remove gaseous components from the solid.

(b) **3**: Dry H_2 (750 mbar) reacted with sample **1** (100 mg) at 25 °C for 4 min, and evacuated (10^{-5} mbar) to remove the gaseous components from the solid.

(c) **4**: Dry H_2 (750 mbar) reacted with sample **1** (100 mg) at −78 °C for 4 min, and evacuated (10^{-5} mbar) at this temperature to remove the gaseous components from the solid.

Procedure for propane metathesis reactions. A mixture of a potential catalytic material (150 mg, 2.26×10^{-2} mmol W) and dry propane (20.4 mmol) was held at 150 °C in a batch reactor of known volume (485 mL) for a 5-day period. At the end of the run, an aliquot was drawn and analyzed by gas chromatography.

Infrared spectroscopy. IR spectra were recorded on a Nicolet 6700 FT-IR spectrometer equipped with a controlled atmosphere cell. Typically, 32 scans were accumulated for each spectrum (resolution 4 cm^{-1}).

Solid-state nuclear magnetic resonance spectroscopy. One-dimensional ^{1}H MAS and ^{13}C CP/MAS NMR spectra were recorded with Bruker AVANCE III spectrometers operating at 400,

500 or 700 MHz resonance frequencies for ^{1}H. ^{29}Si solid-state NMR spectra were recorded with a 400 MHz Bruker AVANCE III spectrometer. Experiments using the 400 MHz spectrometer employed a conventional double-resonance 4 mm CP/MAS probe, and experiments using the 700 MHz spectrometer employed a 3.2 mm HCN triple-resonance probe. For the 100 K experiment with sample **4** using the 400 MHz spectrometer, a Bruker low-temperature 3.2 mm double-resonance probe was employed. In each case the sample was packed into a zirconia rotor under an inert atmosphere inside a glovebox. Dry nitrogen gas was utilized for sample spinning to prevent degradation of the samples. NMR chemical shifts are reported with respect to adamantane as an external reference. For ^{13}C and ^{29}Si CP/MAS NMR experiments, the following sequence was used: 90° pulse on the proton channel (pulse length of 2.4 μs), then a cross-polarization (CP) step with a contact time of typically 2 ms, and finally acquisition of the ^{13}C and ^{29}Si NMR signals under the influence of high-power proton decoupling. The delay between the scans was set to 5 s to allow for the complete relaxation of the ^{1}H nuclear magnetization, and the number of scans ranged between 3000 and 5000 for ^{13}C; between 30 000 and 50 000 for ^{29}Si and 32 for ^{1}H. An exponential apodization function corresponding to a line broadening between 80 and 150 Hz was applied prior to Fourier transformation.

The 2D ^{1}H−^{13}C heteronuclear correlation (HETCOR) solid-state NMR spectroscopy experiments were conducted with a Bruker AVANCE III spectrometer using a 3.2 mm MAS probe. The experiments were performed according to the following scheme: 90° proton pulse, t_1 evolution period under homonuclear ^{1}H-^{1}H decoupling, CP to ^{13}C, and detection of the ^{13}C magnetization under TPPM decoupling. For the cross-polarization step, a ramped radio frequency (RF) field centered at 75 kHz was applied to the protons, while the ^{13}C channel RF field was matched to obtain an optimal signal. A total of 32 t_1 increments with 2000 scans each was collected. The sample spinning frequency was 8.5 kHz. Using a short contact time (0.2 ms) for the CP step, we verified that the polarization transfer in the dipolar correlation experiment was selective for the first coordination sphere about the tungsten: that is, to infer connectivity between pairs of attached ^{1}H−^{13}C spins (direct C−H bonding).

Computational details. *Geometry optimizations and calculations of thermochemical corrections*. All geometry optimizations were performed by using the Perdew, Burke, and Ernzerhof (PBE) generalized gradient approximation (GGA) exchange-correlation (XC) functional, as implemented in the PRIRODA 13 DFT code.[2] All electron basis sets (L1) comparable in quality to the correlation consistent valence double-ζ plus polarization (cc-PVDZ) basis sets of Dunning were used.[3] All stationary geometries were characterized by an analytically calculated Hessian matrix. Possible scalar relativistic effects (for tungsten) were taken into account *via* the Dyall Hamiltonian.[4]

The default, adaptively generated PRIRODA grid, corresponding to an accuracy of the exchange-correlation energy per atom of 1×10^{-8} Hartree was decreased by a factor of 100 for a more accurate evaluation of the exchange-correlation energy. Default values were used for the self-consistent-field (SCF) convergence and the maximum gradient for geometry optimization criterion (1×10^{-4} au), whereas the maximum displacement geometry convergence criterion was decreased to 0.0018 au.

Translational, rotational, and vibrational partition functions for thermal corrections used to arrive at the total Gibbs free energies were computed within the ideal-gas, rigid-rotor, and harmonic oscillator approximations. The temperature used in the calculations of thermochemical corrections was set to 298.15 K in all the cases.

Single-point (SP) energy evaluations. The energies were re-evaluated at the optimized geometries by means of the M06 functional[5] as implemented in the ORCA code.[6] All electron def2-tzvpp basis sets of Ahlrichs' group were used with corresponding density-fitting basis sets on all elements but W.[7,8] Quasi-relativistic effective core potentials (ECP) of the Stuttgart type[9] were used to describe the 60 inner electrons of W in combination with a corresponding def2-tzvpp basis set. The "TightSCF" option (energy change 1.0 e^{-08} au) was adopted for the SCF procedure. Numerical integrations of the XC terms were performed using the tighter-than-default "Grid 6" option (Lebedev590 and IntAcc = 5.34 and no FinalGrid) to reduce the potential numerical noise.

Minimum energy crossing point (MECP) calculations. The closed shell species **IV**, **VIII**, **XII**, and **XVI** can undergo reductive elimination to form a triplet W(IV) species with the release of one methane molecule. An estimation of a barrier between the stationary points of different multiplicities is not a trivial task, and usually requires finding the point of the minimum energy of the crossing potential energy surfaces (PES) of different spin-states.

To locate the MECP, the following procedure was applied: (a) the potential energy surface scan along C (of a CH_3 group)–H (of a hydride) was performed as implemented in PRIRODA 13 code for multiplicities M = 1 and M = 3; (b) the PES of M = 1 and M = 3 were plotted with respect to the C–H distance (scan optimization step), and a preliminary geometry guess for the MECP was obtained; (c) MECP optimization of these structures was done as implemented in ORCA code. The PBE functional was used with all electron def2-svp basis sets of Ahlrichs groups with corresponding density-fitting basis sets on all elements but W.[7,8] Quasi-relativistic ECP of the Stuttgart type[9] were used to describe 60 inner electrons of W in combination with corresponding def2-svp basis sets. The "TightSCF" option (energy change 1.0 e^{-08} au) was adopted for the SCF procedure. Numerical integrations of the XC terms were performed using the tighter-than-default "Grid 6" option (Lebedev590 and IntAcc = 5.34 and no FinalGrid) to reduce the potential numerical noise; (d) SP energy evaluation (*vide supra*) was done on the thus-obtained MECP structure; (e) for consistency, and only for multiplicity change barriers, the initial complexes

(reactants) **IV**, **VIII**, **XII**, and **XVI** were re-optimized in ORCA followed by a standard SP energy evaluation. Because optimizations both in PRIRODA and ORCA were done with the PBE GGA functional and with basis sets of similar quality (L1 and def2-svp), the re-optimization took only few steps; (f) Eventually, the free energy barrier associated with the W(VI) → W(IV) transformation was approximated as $\Delta G^{\ddagger} = E(\text{MECP}) - E(\text{reactant})$ because only minor entropy changes were expected.

Periodic calculations. The 001-4 periodic model from the work of Sautet *et al.*[10] was chosen to mimic the bulk silica surface.[11] The PBE GGA XC functional as implemented in the VASP code[12-16] using plane wave basis sets was employed for geometry optimizations to maintain consistency with our cluster model calculations. The projector augmented wave approach was used to describe the interactions between the valence and core electrons.[17] The Brillouin zone was sampled with a 1 × 1 × 1 Monkhorst-Pack *k*-point grid. All other parameters were kept at their default values. The box size of 22.027 Å × 22.027 Å × 25.000 Å was kept during all the optimizations. First, the metal-free silica structure was fully re-optimized without any constraints. Then the hydrogen atom of the central silanol group was replaced with a W-containing species. In all subsequent geometry optimizations the nuclear positions of the three bottom layers were fixed.

For the SP energy evaluations, the PBE electronic energy obtained at the optimized geometry was calculated and included a dispersion energy correction *via* the DFT-D3(BJ) scheme.[18]

Calculation of ^{1}H nuclear magnetic shielding and chemical shift values of W-H containing model systems.

All calculations of ^{1}H magnetic shielding values used the Amsterdam Density Functional (ADF) software, ver. 2010.02,[19,20] as distributed by Scientific Computing & Modeling (SCM). The zeroth-order regular approximation (ZORA)[21-23] was used to include relativistic effects, and both scalar as well as spin-orbit[24] effects were included at all stages of computation. All-electron basis sets were used, which were triple-ζ in the valence and included polarization functions (corresponding to the 'TZ2P' basis set, as specified by the SCM documentation). Calculations were performed with the PBE GGA XC functional.[25,26] In many cases, geometry optimizations were performed prior to calculation of the hydrogen magnetic shielding values. Shielding values were computed using the 'NMR' module that is bundled with the ADF software, and converted to chemical shifts using the line of best fit arrived at by considering a number of appropriate benchmarking systems. In particular, these benchmarking systems were chosen such that they contained W-H groups, and possessed high quality structural data (both in terms of diffraction structures and solution state ^{1}H NMR). Crystal structures for these systems can be found in the literature[27,28,29] and are more fully disclosed below. Note that to better model the systems where the experimental ^{1}H shifts were measured in solution, both the geometry optimizations on molecular units extracted from the corresponding crystal structures, as well as for the shielding

calculations, were carried out using the solvent characteristics defined within the conductor-like screening model (COSMO).[30,31] All further details, including detailed specification of all ^{1}H shift values calculated, as well as figures of the systems used both for benchmarking and silica-supported tungsten hydride-containing systems, are outlined below.

X-ray absorption spectroscopy. **In-operando *EXAFS experiments*.** X-ray absorption spectra were recorded at the X-ray beamline 4-1 at the Stanford Synchrotron Radiation Lightsource (SSRL). The storage-ring electron energy was 3.0 GeV. The cryogenic double-crystal monochromator, Si(220) $\varphi = 0$, was detuned by 20% at the W L_{III} edge (10207 eV) to minimize higher-order harmonics present in the X-ray beam.

The sample was prepared in an N_2-filled glovebox with < 1 ppm moisture and < 1 ppm O_2, pressed into a wafer, and mounted in a flow-through cell.[32] The sample mass was 160 mg, calculated on the basis of the tungsten content and the cell dimensions to give a near optimal X-ray absorbance at the W L_{III} edge.

Spectra were collected in transmission mode. A tungsten reference foil was scanned simultaneously for edge energy calibration. The sample was scanned at ambient temperature in the presence of flowing helium for 1 h. The gas flow rate was held constant at 50 mL/min (normal temperature and pressure). EXAFS spectra were recorded, with the interval between the start of one measurement and the next being 15–20 min.

***EXAFS data analysis*.** EXAFS analysis was carried out with the software packages ATHENA and XDAP.[33,34] Reference files, calculated with the FEFF 7.0 code,[35] were used for phase shift and backscattering amplitude corrections. Experimental X-ray diffraction crystallography data characterizing $W(CH_3)_6$[36] were used for the W–C contributions. Reference files for W–O and W–Si contributions were calculated from structure parameters representing the compounds WO_3 and WSi_2, respectively.[37]

The energy of the absorption edge is defined as that at the inflection point of the first absorption peak at the W L_{III} edge. This value was compared with the calibrated edge energy determined for the tungsten reference foil. ATHENA was used for edge calibration and deglitching. XDAP was used for data normalization, background subtraction, and EXAFS data fitting, which was done with a "difference-file" technique[38,39] to determine a best fit based on a comparison of data with overall fits as well as fits of individual shell contributions. In the XDAP code, the disorder term (Debye-Waller factor) and inner potential correction (ΔE_0) are with respect to the corresponding reference files.

Fitting was an iterative process that continued until the parameters characterizing each of the proposed shells and the overall fit were in good agreement with the k^1- and k^3-weighted EXAFS data (k is the wave vector) and the Fourier-transformed data. Fitting was done iteratively for each of the individual shells, and all the fitting parameters were required to have physically appropriate

values for an acceptable fit. The number of usable parameters in the fitting was limited by the Nyquist theorem (a statistical criterion): $n = 2\Delta k\Delta r/\pi + 2$, where Δk and Δr are the ranges in k space used in the fitting and the range in R (distance) space fit in the Fourier Transform.

2. Microelemental and GC analysis of 1-4

Table S1 Elemental and GC analysis data

Compound	CH_4/W^a	C/W^b	Element (%)	
			C	W
**1**: [$WMe_5/SiO_{2\text{-}700}$]	NA[c]	5.3	0.97	2.77
**2**: [WH@150 °C]	4.9 ± 0.3	0.6	0.11	2.75
**3**: [WH@25 °C]	4.0 ± 0.3	1.3	0.24	2.74
**4**: [WH@-78 °C]	1.7 ± 0.3[d]	ND[e]	-	-
**4**: [WH@-78 °C][f]	-	2.1	0.38	2.76

[a] Ratios were obtained from GC quantification of the released CH_4 gas during preparation of the compounds **2**, **3** and **4** by hydrogenolysis of **1**. [b] Calculated from microanalysis of the compounds. [c] Not applicable. [d] The lower experimental values (1.7 ± 0.3) from what expected (3) could be due to partial adsorbtion of CH_4 molecules onto the material at this sample collecting temperature (-78 °C). [e] Not detected, since elemental analysis could not be conducted at low temperature. [f] After warming **4** to room temperature.

3. IR spectra

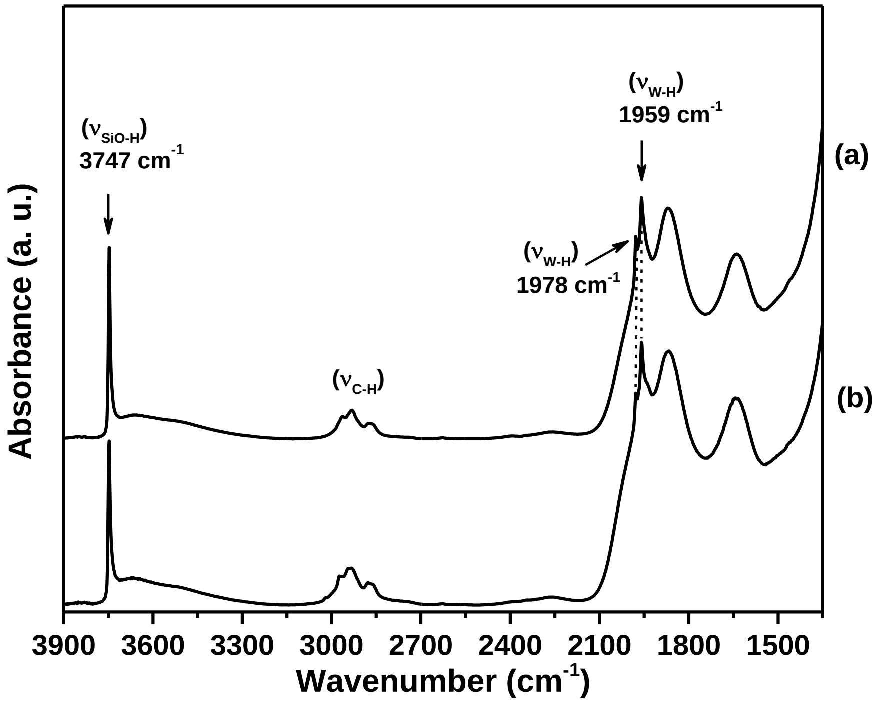

Fig. S1 IR spectra of silica-supported tungsten hydrides synthesized from **1** at 0 (a) and -20 °C (b).

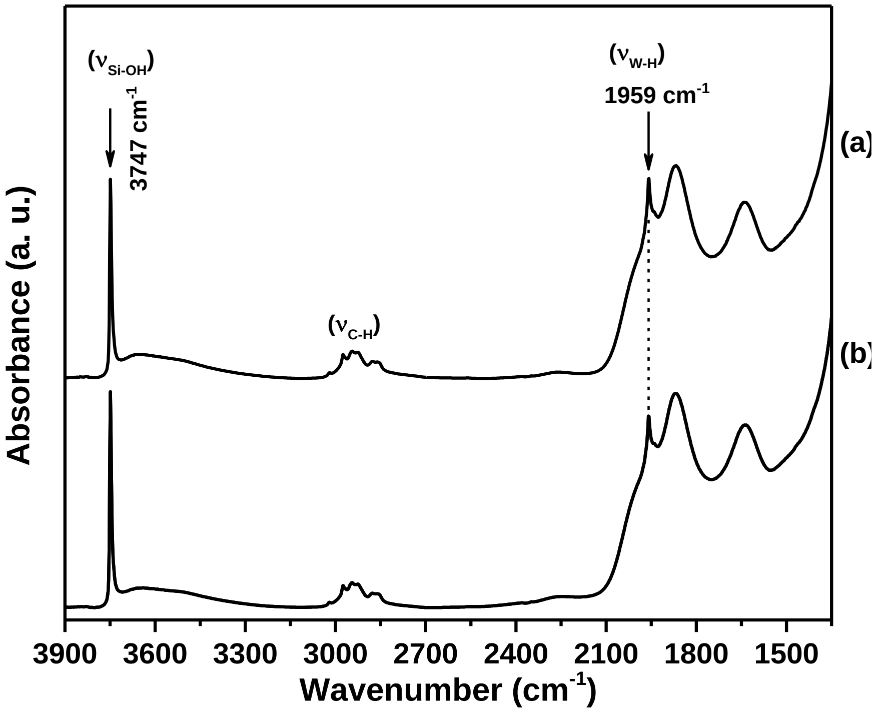

Fig. S2 IR spectrum of **4** (a) and its invariance after maintaining the sample at 77 K for 2 h (b).

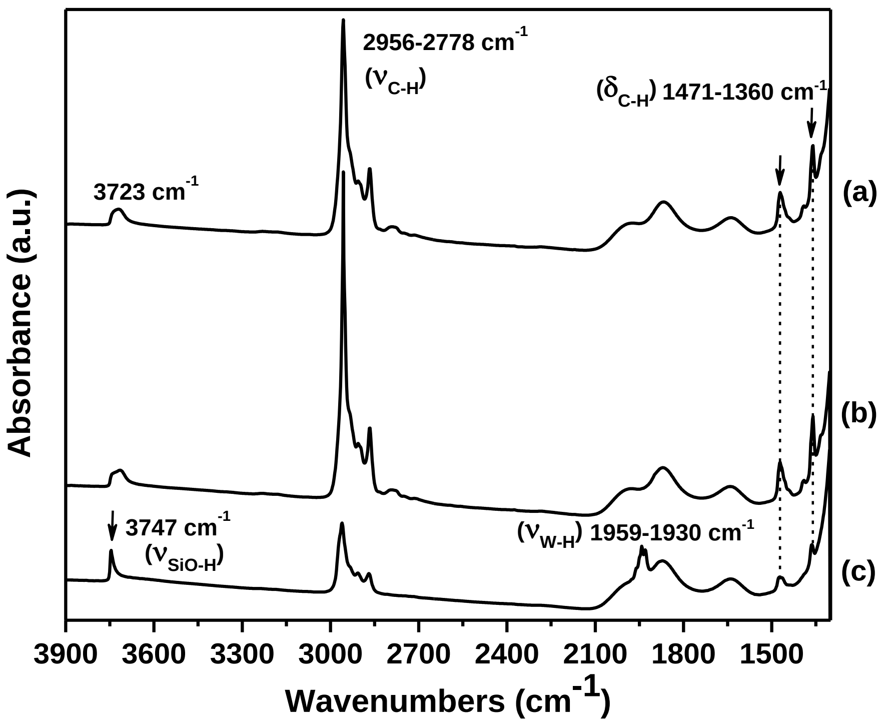

Fig. S3 IR spectra of (a) silica-supported Schrock complex [(≡Si–O–)W(≡C-*t*-Bu)(CH_2–*t*-Bu)$_2$], (b) after attempted hydrogenolysis at -78 °C and (c) after hydrogenolysis at 25 °C.

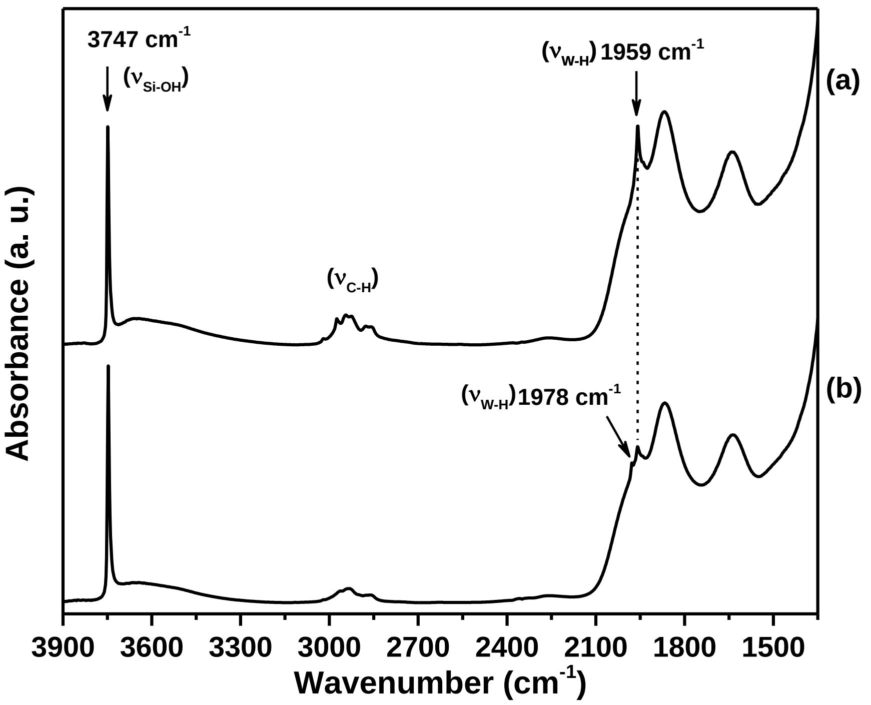

Fig. S4 IR spectrum of fresh **4** (a) and after storage at room temperature (b).

4. Solid-state NMR investigation

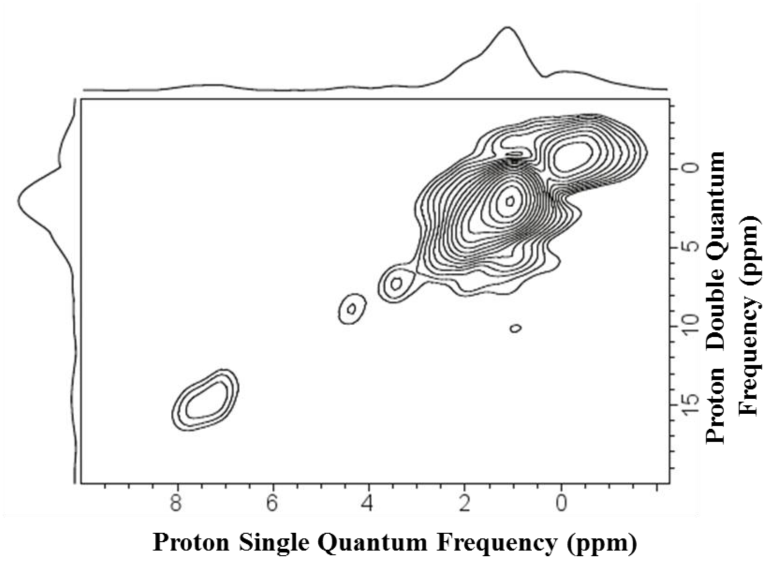

Fig. S5 Two-dimensional (2D) ^{1}H-^{1}H double-quantum (DQ)/single-quantum (SQ) NMR spectrum of **2**, acquired using 500 MHz NMR spectrometer (B_0 = 11.7 T), under 22 kHz MAS with a back-to-back recoupling sequence, number of scans per t_1 increment = 128, repetition delay = 5 s, number of t_1 increments = 128.

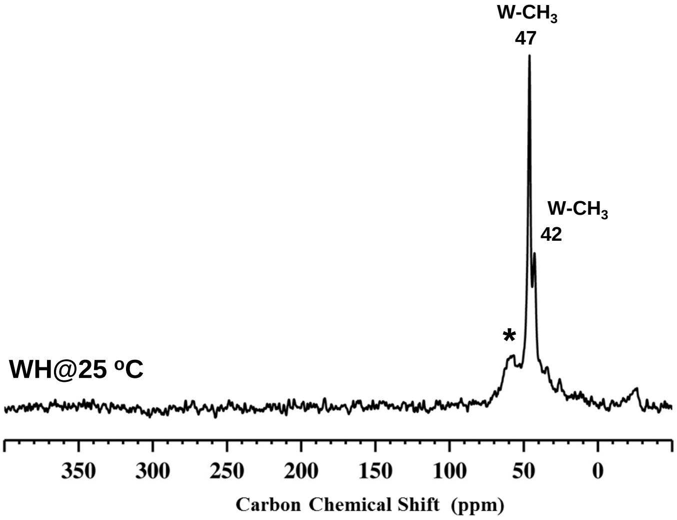

Fig. S6 One-dimensional (1D) ^{13}C CP/MAS NMR spectrum of **3** recorded at room temperature and 100 MHz ^{13}C frequency (i.e., B_0 = 9.4 T) with a 10 kHz MAS frequency, 25 000 scans, a 4 s repetition delay, and a 2 ms comtact time. Exponential line broadening of 80 Hz was applied prior to Fourier transformation. *The weak peak at 59 ppm is attributed to the formation W-OCH_3 moieties due to the contamination of O_2 in the NMR rotor as an effect of prolongued acquisition times.

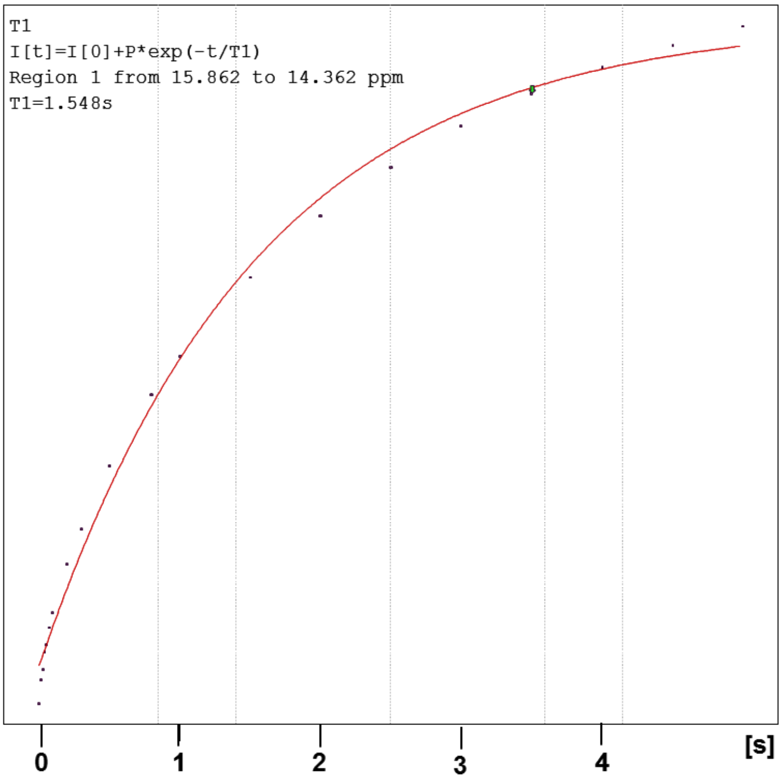

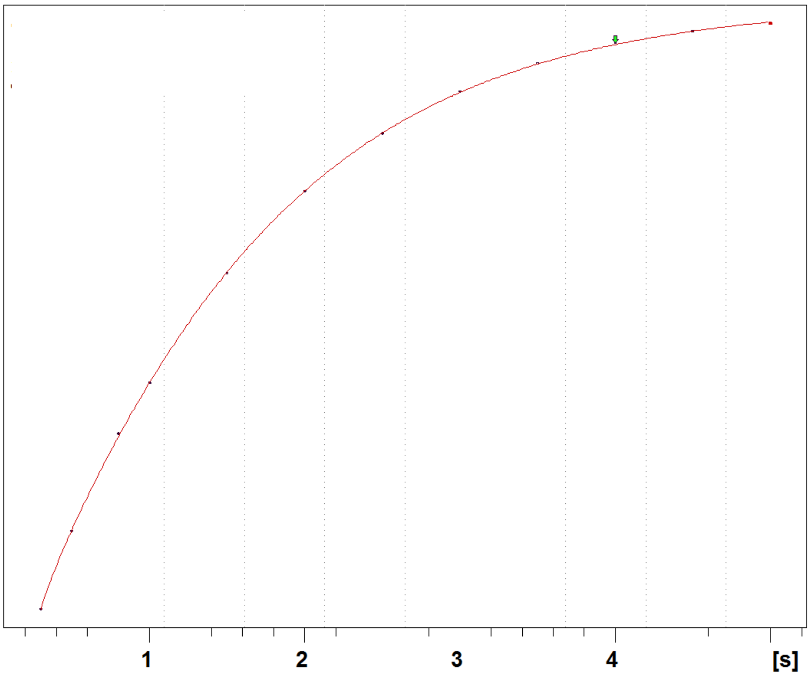

Fig. S7 Saturation-recovery spin-lattice (T_1) relaxation measurement for **4** after warming to room temperature in the region 15.8 to 14.3 ppm with corresponding data fit provided in red. Plots correspond to (top) mono-exponential decay and (bottom) stretched exponential decays with 4 components.

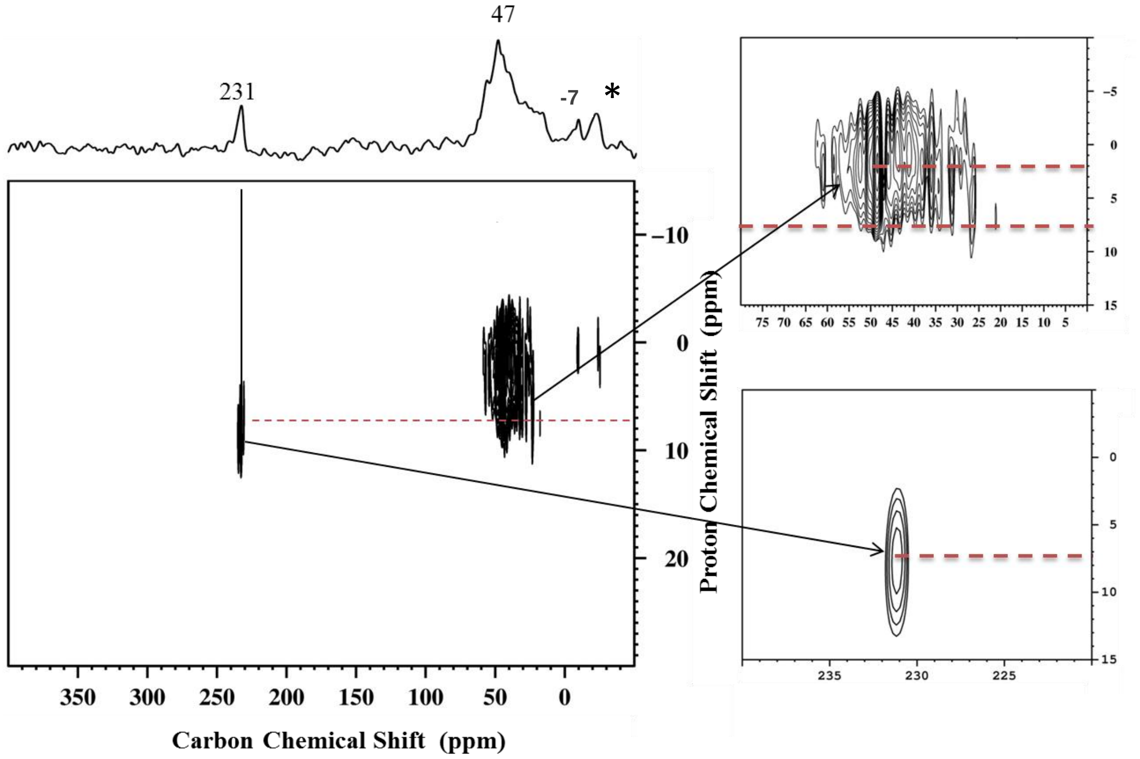

Fig. S8 Two dimensional (2D) ^{1}H-^{13}C CP/MAS dipolar HETCOR spectrum (acquired with 10 kHz MAS frequency, 4000 scans per t_1 increment, a 4 s repetition delay, 64 individual t_1 increments, and a 0.2 ms contact time). *The peak at -24 ppm in the correlation spectra can be attributed to the adsorbed reactant $ZnMe_2$.[40]

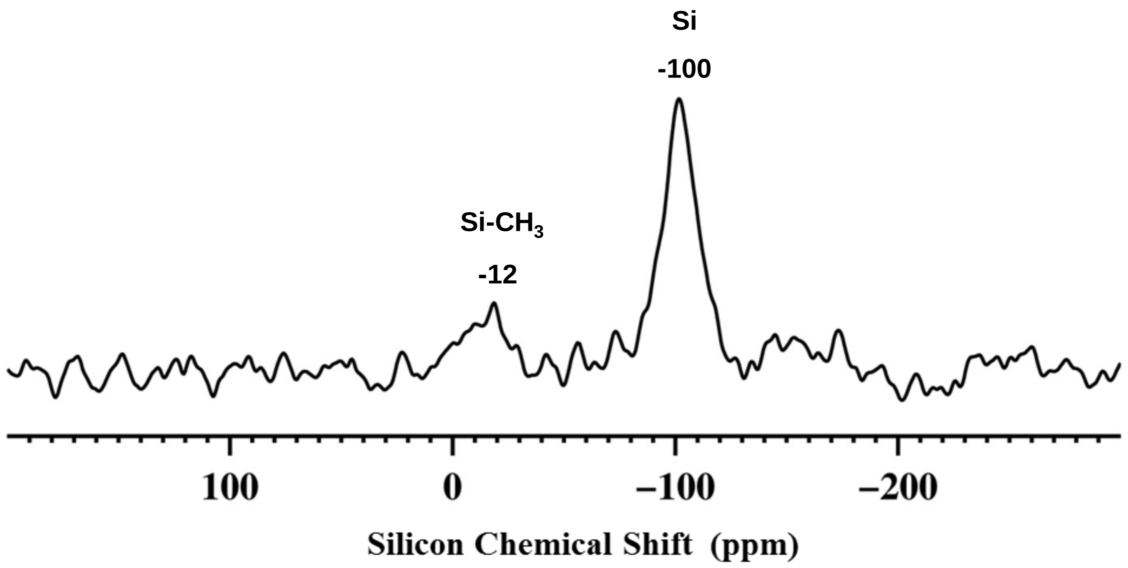

Fig. S9 ^{29}Si CP/MAS NMR spectrum recorded at room temperature of **4** after warming up to room temperature, acquired at 79.4 MHz with a 5 kHz MAS frequency. The number of scans was 20 000, and the recycle delay was 5 s. A cross polarization time of 5 ms was used. An exponential line broadening of 100 Hz was applied prior to Fourier transformation.

5. EXAFS investigation of 1

XDAP was used to generate EXAFS models that could be compared to the EXAFS data of **1**. The quality of the fit is determined based upon the goodness of fit $(\Delta X)^2$, indicating the deviation between the model and the data, and if the values of the fitted parameters make good chemical sense. As stated in the main text, **1** was changing under the influence of the beam requiring analysis of the first and fourth scans independently.

The starting material was speculated to be [(≡Si–O–)W$(CH_3)_5$]. Multiple models were compared to the data of scan 1. Table S2 and Figure S10 show the best fit model, that within error, agrees with the starting material [(≡Si–O–)W$(CH_3)_5$]. Alternate models were also compared to the data. Models varied the coordination of the W-O and W-C contributions. These models were deemed unfit due to Debeye Waller factors and inner potentials that were deemed to be outside of range of acceptable. When comparing the plots of the models to the data the overall k space appeared adequate but when comparing the k^1 and k^3 weighted R space it was evident that the models did not match the data.

Analysis of the fourth scan of the EXAFS was fit to the form of [(≡Si–$(O)_Y$)–W$(CH_3)_X$] with the anticipation that the overall podality of the tungsten was changing under the influence of the beam, which is similar to long term storage at room temperature. Due to the complexity of the silica surface it is expected that multiple species now exist as postulated by the NMR, resulting in a greater uncertainty in the optimal fit. Multiple models were tested varying the oxygen and carbon contributions. Models were also tested that included a $=CH_2$ contribution. All models except the one presented in Table S3 and Figure S11 were rejected due to unrealistic parameters, mainly Debeye Waller factors and inner potentials, or due to the model not aligning well with the data when comparing the *k* space, R space, and individual shell fits.

Table S2 EXAFS model considered representing [(≡Si–O–)W(CH_3)$_5$] sample, **1**. The model was chosen for its realistic coordination shells and good fit of the data. The fit was optimized to give the lowest goodness of fit value, (ΔX)^2, indicating minimal deviation between the model and the data.

**Model**	**(ΔX)^2**	**Coordination shell**	***N***	***R* (Å)**	**10^3 x $\Delta\sigma^2$ (Å^2)**	**ΔE_0 (eV)**	**Reasons for model Acceptance**
[(≡Si–O–)W(CH_3)$_5$]	3.83	W–C_{Methyl}	4.9	2.02	8.6	4.86	This model is recommended, as it has the lowest goodness of fit value of the models tested. The ΔE_0 values are within the physically acceptable range. Coordination numbers and distances are all appropriate.
		W–$O_{support}$	1.0	1.81	3.7	-3.73	

Table S3 EXAFS model considered for the [(≡Si–O–)W(CH_3)$_5$] sample, **1** after 45 minutes of helium flow and exposure to the X-ray beam. The model was chosen because the parameter values are physically realistic and appropriate. The fit was optimized to give the lowest goodness of fit, $(\Delta X)^2$, indicating minimal deviation between the model and the data. The relatively large value of ΔE_0 characterizing the W–C contribution of the model is explained by the small number of scans (one) dictated by the changes occurring in the sample.

Model	$(\Delta X)^2$	Coordination shell	*N*	*R* (Å)	10^3 x $\Delta\sigma^2$ (Å^2)	ΔE_0 (eV)	Reasons for model Acceptance
[(≡Si–(O)$_Y$)–W(CH_3)$_X$]	2.87	W–C_{Methyl}	3.8	2.00	12.0	14.60	This model has the lowest goodness of fit value of the ones tested. One of the ΔE_0 values is within the range considered appropriate, but the other is outside the range, but it is considered acceptable because of the challenge of determining a good fit with multiple species present. The coordination numbers and distances make good physical sense.
		W–$O_{support}$	1.6	1.82	9.78	-4.23	

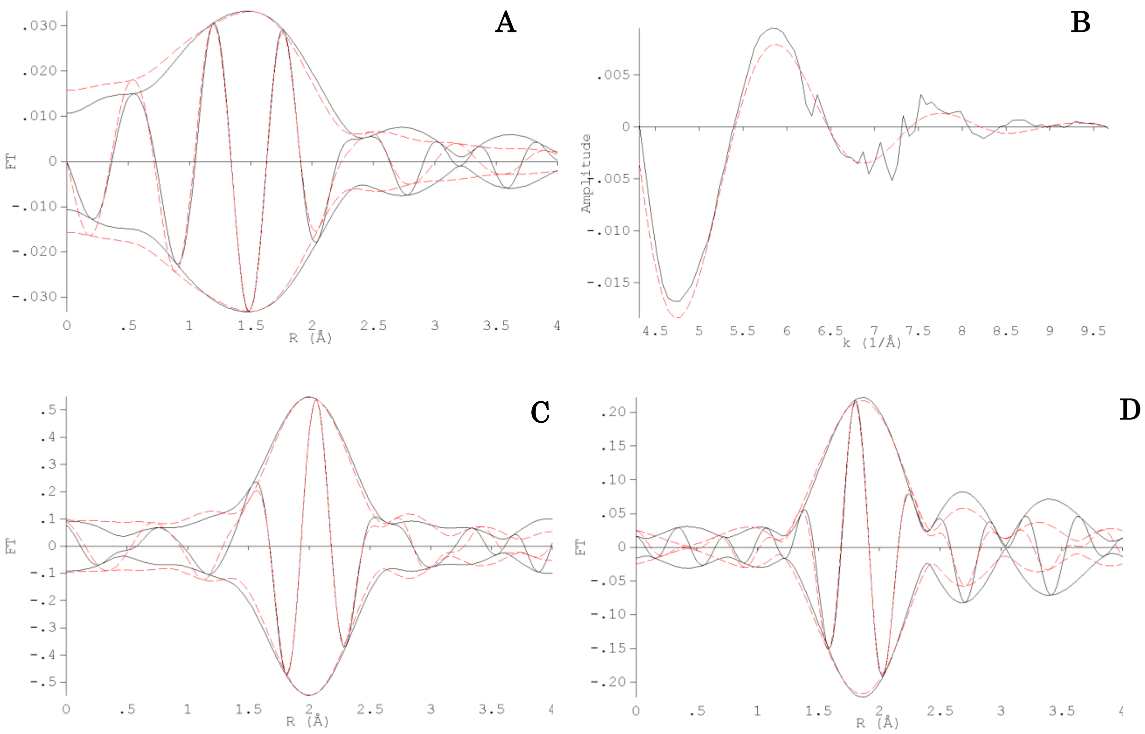

Fig. S10 EXAFS data and fits characterizing the sample initially consisting of silica-supported tungsten pentamethyl in flowing helium at 298 K and 1 bar. Fitting range: k = 4.32–9.66 $Å^{-1}$; goodness of fit = 3.83. **A**, k^1-weighted data (EXAFS function, solid line) and sum of the calculated absorber–backscatterer contributions (dashed line). **B**, k^1-weighted imaginary part and magnitude of the Fourier transform of the data (solid line) and sum of the calculated contributions according to the best-fit model (dashed line). **C**, k^1-weighted, phase-corrected, imaginary part and magnitude of the Fourier transform of the data (solid line) and calculated contribution according to the best-fit model (dashed line) of the W–C shell. **D**, k^1-weighted, phase-corrected imaginary part and magnitude of the Fourier transform of the data (solid line) and calculated contribution (dashed line) of the W– O shell.

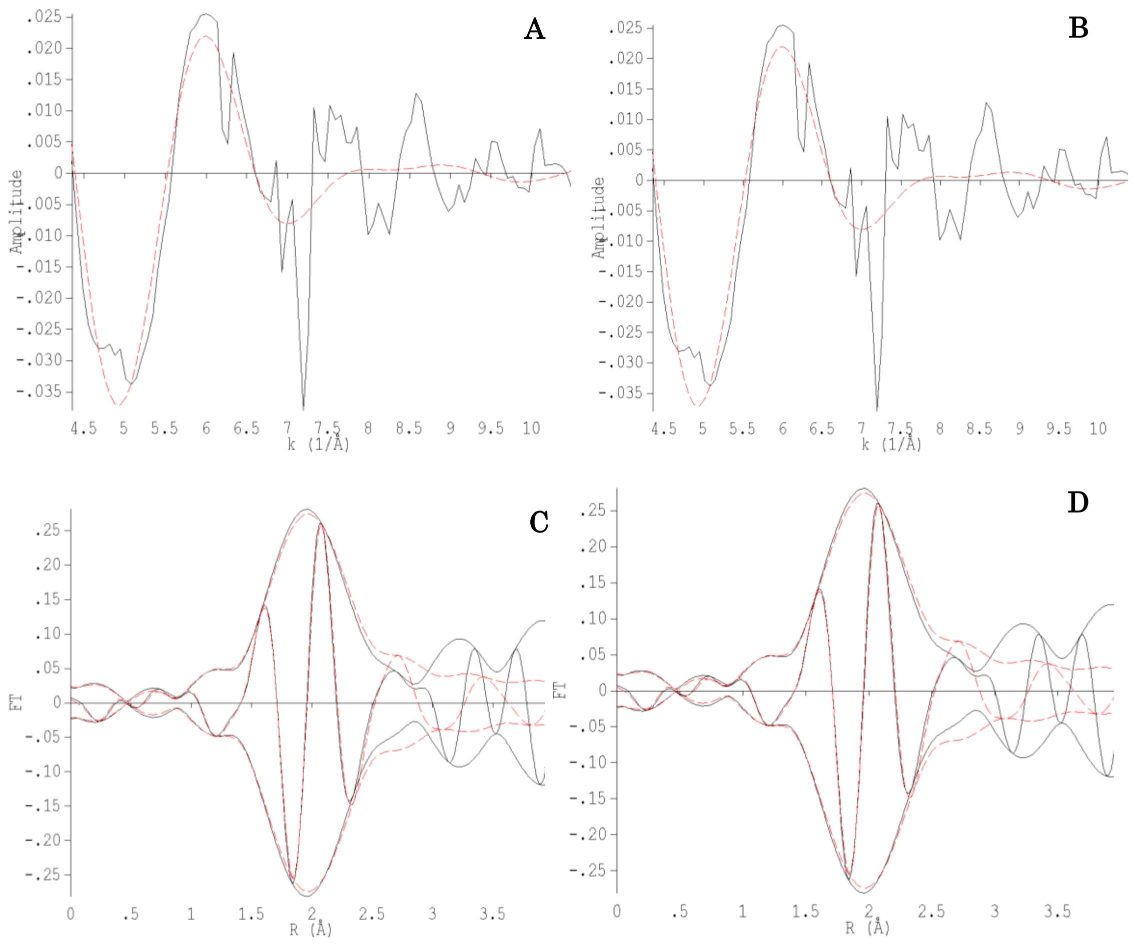

Fig. S11 EXAFS data characterizing the sample initially consisting of silica-supported tungsten pentamethyl after 45 min in flowing helium at 298 K and 1 bar. Fitting range: k = 4.36–10.43 Å^{-1}; goodness of fit = 2.87. **A**: k^1-weighted data (EXAFS function, solid line) and sum of the calculated absorber-backscatterer contributions (dashed line). **B**: k^1-weighted imaginary part and magnitude of the Fourier transform of the data (solid line) and sum of the calculated contributions according to the best-fit model (dashed line). **C**: k^1-weighted, phase-corrected, imaginary part and magnitude of the Fourier transform of the data (solid line) and calculated contribution according to the best-fit model (dashed line) of the W–C shell. **D**: k^1-weighted, phase-corrected imaginary part and magnitude of the Fourier transform of the data (solid line) and calculated contribution (dashed line) of the W–O shell.

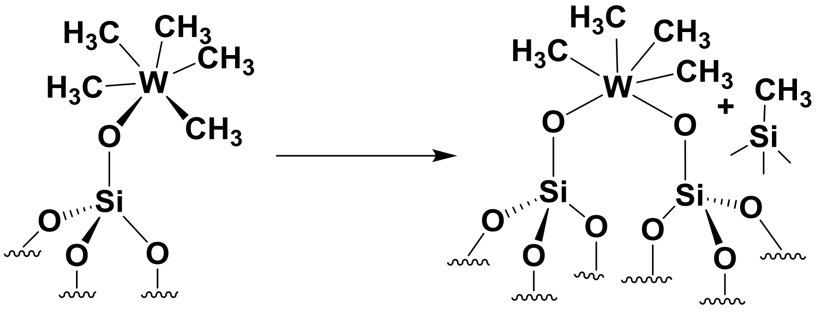

(1)

Scheme S1 Transformation of monopodal surface species **1** [(≡Si–O–)W(CH_3)$_5$] into the bipodal species through migration of methyl (-CH_3) group as suggested by EXAFS analysis.

6. Additional details pertaining to the computation of hydrogen magnetic shielding and ^{1}H chemical shift values.

As specified in the main text, representative systems were selected to establish the applicability of the density functional theory (DFT) method that was chosen to calculate the various magnetic shielding (and ultimately chemical shift) values. The systems chosen supplement the other W-containing systems chosen as part of an earlier study,[1] with the key difference in the present study being the inclusion of molecules which contain W–H groups. In many other respects, the general approach remains the same as specified in the earlier account. The new systems under consideration are depicted in Figure S12.

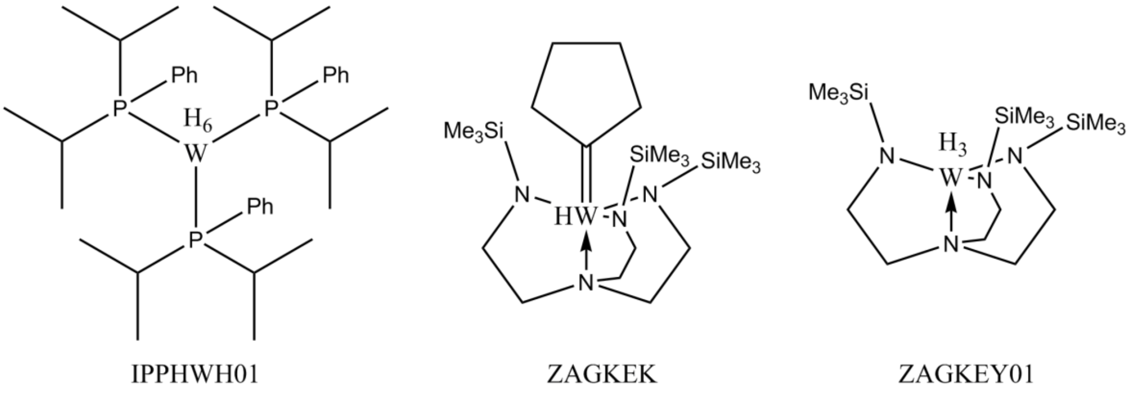

Fig. S12 Tungsten hydride-containing systems chosen to aid in benchmarking the magnetic shielding values calculated via the zeroth-order regular approximation (ZORA) DFT method. The short string of text written below each structure corresponds to the crystal structure reference ID, as reported in the Cambridge Structural Database (CSD). Original literature references for the crystal structures are found in references 41-43. Optimized molecular coordinates under

appropriate COSMO settings can be found in the coordinates disclosure sections below.

Subsequent to a full geometry optimization including non-explicit solvent effects via the COSMO, hydrogen magnetic shielding calculations were performed. By merging the data generated at as part of the present study, with that which was published in the recent study on surface-supported W-containing species (as disclosed above), we arrive at the following plot which correlates the computed hydrogen magnetic shielding values with experimental ^{1}H chemical shift values (Figure S13):

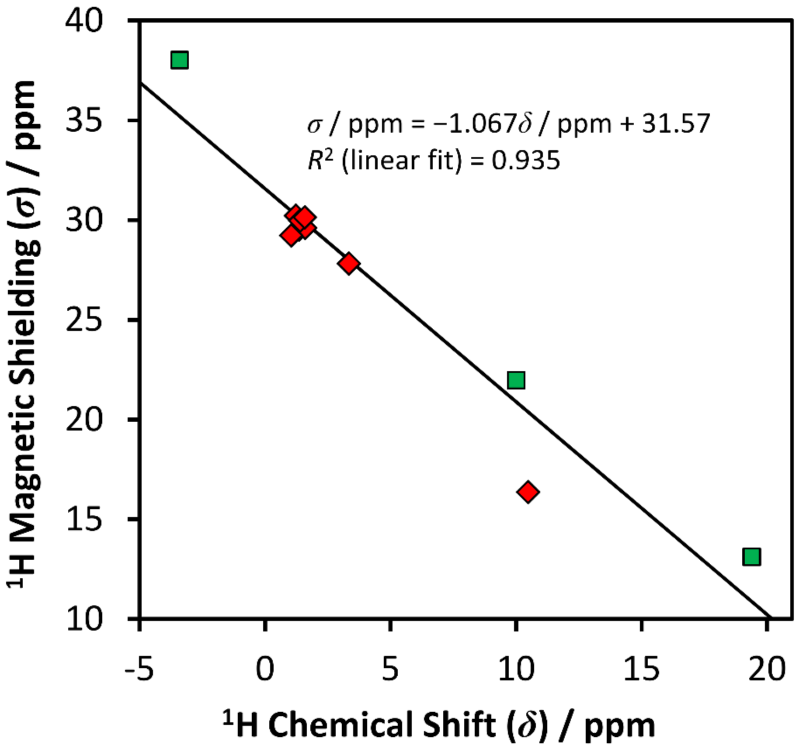

Fig. S13 Correlation plots relating isotropic hydrogen magnetic shielding and experimental ^{1}H isotropic chemical shift values (in ppm) for the calibration molecules featured in Figure S12 (green data points), in combination with those specified in the earlier account of (red data points).[1] Linear regression fit to all data and Pearson R^2 value appears in the upper right the plot. This equation was subsequently used to transform calculated isotropic hydrogen magnetic shielding values for the tungsten hydride model systems into calculated isotropic ^{1}H chemical shift values.

With the ^{1}H magnetic shielding/chemical shift correlation curve established using the data summarized in Figure S13, we now discuss the models chosen to represent the possible surface-supported species found in **2**, **3**, and **4**. As noted in the main text, and corroborated with additional DFT computations that highlighted a variety of possible products and their relative free

energies, it is believed that one of the major products created as a result of hydrogenolysis at 150 °C would be the homoleptic tungsten pentahydride, [(≡Si–O–)WH_5]. When considering this species for hydrogen magnetic shielding calculations, we employed two slightly different surface models to establish if the choice of surface greatly influences the resulting calculated ^{1}H shift values. The first model surface (model ‘A’) is derived in a similar fashion to that presented in the literature using other silica-supported W-containing species.[1] Pictured in Figure S14 (left) is the resulting structure which is obtained, commencing from this literature account, and after the appropriate modification of the surface-supported species and subsequent geometry optimization of the supported species, as well as the terminating H atoms of the surface itself.

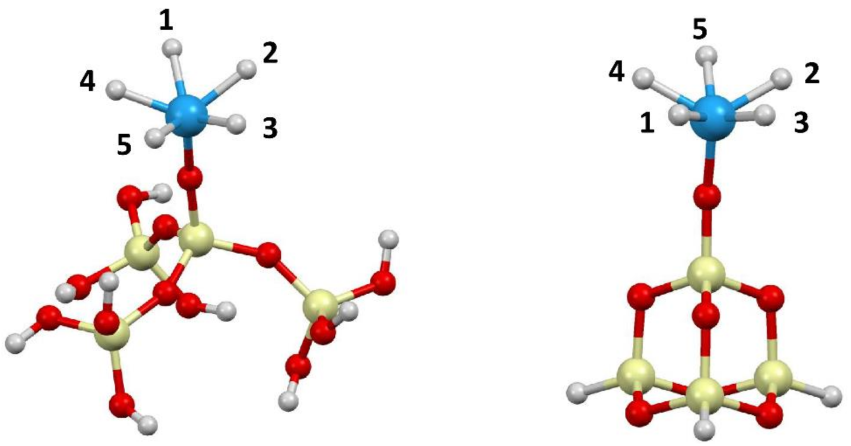

δ(^{1}H, avg$_{(1\text{-}5)}$) = 13.8 ppm δ(^{1}H, avg$_{(1\text{-}5)}$) = 12.9 ppm

Fig. S14 Depictions of the optimized model structures used to model [(≡Si–O–)WH_5]. On the left, the silica surface model used is similar to what was established in an earlier account by Samantary *et al.*, (‘model A’) whereas on the right, a very slightly altered model based upon structure XVIII (main text, Scheme 2, ‘model B’) is used. Site labels are provided and can be referred to when consulting Table S4, where we report the calculated hydrogen magnetic shielding and ^{1}H chemical shift values for a variety of model structures.

In addition to this species, species ‘XVIII’ (see Scheme 2 in the main text, and referred to as model ‘B’ in Table S4) was also considered as an adequate surface model, and after a very minor geometry optimization from that reported in the other portions of this report (i.e., by ‘minor,’ the all-atom root-mean squared deviation (RMSD) between XVIII and this newly ‘optimized’ XVIII used for NMR property calculations was 0.054 Å), it was found that, on average, the calculated ^{1}H chemical shift values are comparable to what was reported using surface model ‘A’. The primary item to note when contrasting the NMR property calculations with each particular surface model structure is that for the model ‘A,’ there is a relatively large variation in the ^{1}H chemical

shifts calculated for each H atom (ranging from 12.1 to 15.6 ppm), whereas this variation is greatly muted for model 'B' (12.4 to 13.2 ppm). Although under the conditions at which the ^{1}H NMR experiments were carried out we fully expect these values to be averaged by the fast exchange between the various sites, it is clear that surface modifications can be manifested in experimental ^{1}H shift deviations for tungsten hydrides, and this effect is on the order of a few ppm. A detailed disclosure of the computed hydrogen shielding information is given in Table S4.

Table S4 Calculated hydrogen magnetic shielding and ^{1}H chemical shifts for models of silica-supported tungsten hydrides of the form [(≡Si–O–)WH_5].[a]

surface model	site label	σ_p / ppm[b]	σ_d / ppm[c]	σ_{SO} / ppm[d]	σ_{iso} / ppm[e]	δ_{iso} / ppm[f]
model 'A'	1	−4.364	27.620	−6.088	17.168	13.491
	2	−4.389	27.706	−4.653	18.664	12.085
	3	−4.264	27.797	−6.016	17.517	13.163
	4	−4.264	27.655	−8.483	14.909	15.609
	5	−4.358	27.978	−7.883	15.737	14.831
	avg.	—	—	—	**16.802**	**13.836**
model 'B'	1	−4.377	28.284	−6.429	17.478	13.202
	2	−4.328	28.343	−6.113	17.902	12.805
	3	−4.360	28.277	−6.392	17.525	13.158
	4	−4.308	28.338	−6.058	17.972	12.739
	5	−4.208	28.356	−5.852	18.296	12.436
	avg.	—	—	—	**17.835**	**12.868**

[a] As depicted in Figure S14. [b] Paramagnetic shielding contribution. [c] Diamagnetic shielding contribution. [d] Spin-orbit shielding contribution (relativistic contribution). [e] The isotropic magnetic shielding values in this column are a simple linear combination of the three preceeding columns (i.e., $\sigma_{iso} = \sigma_d + \sigma_p + \sigma_{SO}$). [f] The isotropic chemical shift values were determined using the relationship depicted in Figure S13.

Importantly, it is evident that it is the relativistic spin-orbit mechanism which appears to be responsible for much of the difference in the various computed shifts for these model structures of [(≡Si–O–)WH_5]. This point informs us that non-relativistic computations would not be able to capture much of the observed chemical shift behavior, in addition to predicting that the ^{1}H chemical shift values of the W–H sites would be considerably lower (ca. 7.8 ppm if relativistic effects were ignored).

For the other surface-supported species, additional model systems were also considered. In Figure S15, pictures of these models are provided, and the hydrogen magnetic shielding and ^{1}H

chemical shift values are given in Table S5. Atomic coordinates for all systems are presented in this SI.

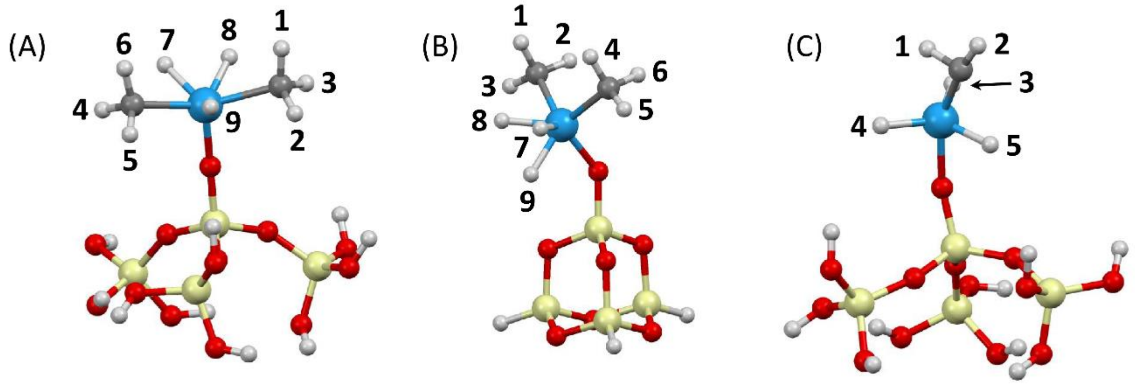

Fig. S15 In (A) and (B), two different silica surface models are used with the supported species being [(≡Si–O–)$WH_3(Me)_2$] in both cases (denoted again as model 'A' and model 'B', respectively, in Table S5). Note that in (B), the structure is identical as provided in structure 'XII' in Scheme 2 of the main paper. Note also that the H_3 group faces away from the silica surface in (A), whereas it points towards the surface in (B). As expected, this has a significant influence on the computed ^1H shift values (Table S5). In (C), we provide the model used for [(≡Si–O–)$WH_3(=CH_2)$]. Note the almost bridging nature of one of the hydrogen atoms in the alkylidene moiety.

Table S5 Calculated hydrogen magnetic shielding and ^1H chemical shifts for the model silica-supported tungsten hydrides of the form [(≡Si–O–)$WH_3(Me)_2$] and [(≡Si–O–)$WH_3(=CH_2)$].[a]

surface model	site label	σ_p / ppm[b]	σ_d / ppm[c]	σ_{SO} / ppm[d]	σ_{iso} / ppm[e]	δ_{iso} / ppm[f]
			[(≡Si–O–)$WH_3(Me)_2$]			
model 'A'	1	3.002	27.382	−0.608	29.776	1.677
	2	0.339	27.632	−0.580	27.391	3.912
	3	0.250	27.693	−1.746	26.197	5.031
	avg.	—	—	—	**27.788**	**3.540**
	4	0.444	27.327	−1.081	26.690	4.569
	5	0.429	27.495	−0.519	27.405	3.899
	6	2.925	27.317	−0.424	29.818	1.638
	avg.	—	—	—	**27.971**	**3.369**
	7	−4.621	27.261	−7.077	15.563	14.997
	8	−4.608	27.068	−10.969	11.491	18.813
	9	−4.536	27.289	−4.648	18.105	12.615

	avg.	—	—	—	**15.053**	**15.475**
model ‘B’	1	2.894	27.159	0.037	30.090	1.383
	2	1.295	27.127	0.044	28.466	2.905
	3	1.534	27.317	−0.657	28.194	3.160
	avg.	—	—	—	28.917	2.483
	4	2.894	27.159	0.037	**30.09**	**1.383**
	5	1.534	27.317	−0.657	28.194	3.160
	6	1.295	27.127	0.044	28.466	2.905
	avg.	—	—	—	**28.917**	**2.483**
	7	−0.966	26.915	−7.953	17.996	12.717
	8	−0.966	26.915	−7.953	17.996	12.717
	9	2.905	27.123	−5.474	24.554	6.571
	avg.	—	—	—	**20.182**	**10.668**
$[(\equiv Si–O–)WH_3(=CH_2)]$						
	1	−2.313	31.125	0.816	29.628	1.816
	2	−6.387	32.75	−3.23	23.133	7.903
	avg.	—	—	—	**26.380**	**4.859**
—	3	3.160	27.632	−6.584	24.208	6.895
	4	3.178	28.003	−6.580	24.601	6.527
	5	1.602	27.681	−5.376	23.907	7.177
	avg.	—	—	—	**24.239**	**6.867**

[a] As depicted in Figure S15. [b] Paramagnetic shielding contribution. [c] Diamagnetic shielding contribution. [d] Spin-orbit shielding contribution (relativistic contribution). [e] The isotropic magnetic shielding values in this column are a simple linear combination of the three preceeding columns (i.e., $\sigma_{iso} = \sigma_d + \sigma_p + \sigma_{SO}$). [f] The isotropic chemical shift values were determined using the relationship depicted in Figure S13.

It is again clear that depending upon the silica surface chosen to anchor the W–H species, there is a substantially large variation in the computed ^1H chemical shift values (e.g., contrast model ‘A’ and model ‘B’ for $[(\equiv Si–O–)WH_3(Me)_2]$), which predict isotropic (and dynamically averaged) values for the hydrides to be 15.475 and 10.668 ppm, respectively. However, it is important that, in each case, the calculated shift value is predicted to be greater than 10 ppm, which is consistent with the region to which it is assigned in the experimental ^1H solid-state NMR spectra. Furthermore, we note that the computational method appeared to not be quite as successful at predicting the ^1H chemical shift value for the $W=CH_2$ group, as well as the group of three hydrides. The geometry-optimized structure displayed in Figure S15C, which is consistent with that also provided in Structure XIV (Scheme 2 of the main text), shows that the

methylidene group hydrogen atoms appear to be able to bridge the tungsten atom. It is appropriate to note that, in the literature, there are a very large number of examples whereby a hydrogen atom bridges metal centres in polymetallic systems (included those containing tungsten).

7. Computational models, details, and coordinates of all species

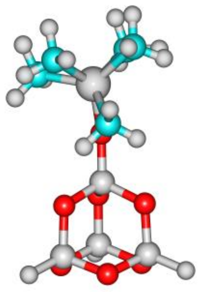
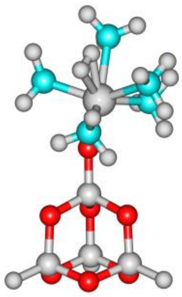
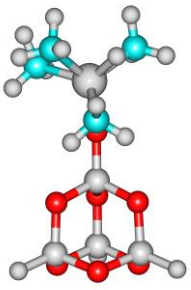

**I**	**I_H$_2$**	**IV**
E (PM): 0.0	E (PM): 0.5	E (PM): -18.1
E (CM): 0.0	E (CM): 2.5	E (CM): -16.1

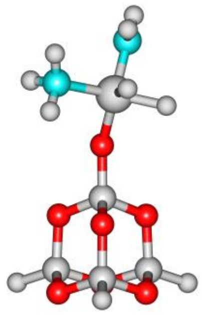
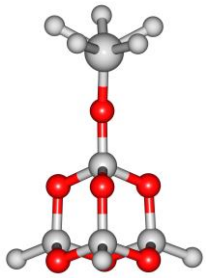

**X**	**XVIII**
E (PM): -43.2	E (PM): -74.5
E (CM): -48.7	E (CM): -77.1

Fig. S16 The relative electronic energies (kcal/mol) of possible intermediates in hydrogenolysis mechanism (Scheme 2) calculated with cluster model (CM) and periodic model (PM).

::::::::::::::

XIV (VASP)

::::::::::::::

E(PBE, VASP) = -2556.50257057 (eV)

Edisp /kcal,au,eV: -597.7703 -0.95260750 -25.9217701

header

1.0000000000000000

22.0272006988999998	0.0000000000000000	0.0000000000000000
0.0000000000000000	22.0272006988999998	0.0000000000000000
0.0000000000000000	0.0000000000000000	25.0000000000000000

Si	O	H	W	C
90	207	58	1	1

Selective dynamics

Direct

0.8918002060665272	0.1013542641683654	0.5759248385131155	T	T	T
0.8898747728415236	0.4338476134267966	0.5757023694586666	T	T	T
0.8890326128348214	0.7676557754756851	0.5761615961284994	T	T	T
0.5567061261349459	0.0998323388892634	0.5766226343041730	T	T	T
0.5541900895362081	0.4337543200860545	0.5745904551631603	T	T	T
0.5542684763572712	0.7677119108048857	0.5768238770725591	T	T	T
0.2245145737846299	0.0976533595005908	0.5745830115380002	T	T	T
0.2224085930064200	0.4311875956128943	0.5745604089997146	T	T	T
0.2215531562297317	0.7640675443094050	0.5749755014132728	T	T	T
0.7882081255362086	0.2013069435230466	0.5727723309654885	T	T	T
0.7871654612319874	0.5347989850825692	0.5728386827474099	T	T	T
0.7863260675932174	0.8685475023731866	0.5731482410623314	T	T	T
0.4542330037492807	0.2009986703717980	0.5713895443167348	T	T	T
0.4532683699278564	0.5346644747163128	0.5682885459403039	T	T	T
0.4513075111635318	0.8683969034947941	0.5717417124095303	T	T	T
0.1223750564827347	0.1989985004858727	0.5697922064964147	T	T	T
0.1203862364774437	0.5323565395125813	0.5694695503095455	T	T	T
0.1196859172922514	0.8662415890942528	0.5703914823550372	T	T	T
0.8372891573883779	0.3224700849102485	0.5146839703367495	T	T	T
0.8359505125457725	0.6562206456019437	0.5147420567029141	T	T	T
0.8369774124321424	0.9901033647450919	0.5165933857034916	T	T	T
0.0034054546812775	0.1519744189480581	0.5085393934490335	T	T	T
0.0014245480270365	0.4851750962830081	0.5087271920463053	T	T	T
0.0004810848878030	0.8188701786753128	0.5088222022444175	T	T	T
0.5044141195009462	0.3213259977181517	0.5120220889512654	T	T	T

0.5037005146475556	0.6562558491164130	0.5120556677507955	T	T	T
0.5038297750476385	0.9887045941341625	0.5139830728713601	T	T	T
0.6689496305349001	0.1544814692613551	0.5119926285090342	T	T	T
0.6676243681864983	0.4880367323246443	0.5121587947375176	T	T	T
0.6665312424288253	0.8217793792408424	0.5121367862559665	T	T	T
0.1711214730806603	0.3197829887123265	0.5101015058234719	T	T	T
0.1696367372365858	0.6530701166250089	0.5099900233748810	T	T	T
0.1705087820477212	0.9869059151903399	0.5117974417050908	T	T	T
0.3358950401685734	0.1529010857852165	0.5097557926950447	T	T	T
0.3338211709057882	0.4860555828889956	0.5096983876130763	T	T	T
0.3327794177996521	0.8197860370792385	0.5100445880765911	T	T	T
0.9252931479857907	0.2479518950073754	0.4412057999999988	F	F	F
0.9234721060137190	0.5812809469992928	0.4411998089999969	F	F	F
0.9216511250117136	0.9146088959913143	0.4411938189999987	F	F	F
0.7529111499777699	0.0692349000150614	0.4404208059999988	F	F	F
0.7510890959842271	0.4025638999803931	0.4404148160000005	F	F	F
0.7492681149822289	0.7358919379987086	0.4404087959999998	F	F	F
0.5919740800133155	0.2461308839970187	0.4390428069999999	F	F	F
0.5901531579838561	0.5794588920069828	0.4390367869999992	F	F	F
0.5883311029915461	0.9127878550180029	0.4390307959999973	F	F	F
0.4195911590101247	0.0674138810145806	0.4382577840000010	F	F	F
0.4177701179914166	0.4007419050047858	0.4382517929999992	F	F	F
0.4159491659989953	0.7340708370086944	0.4382458030000009	F	F	F
0.2586551010217377	0.2443099019962531	0.4368798140000010	F	F	F
0.2568331360090852	0.5776379110049774	0.4368738229999991	F	F	F
0.2550121249987285	0.9109659190149415	0.4368678029999984	F	F	F
0.0862721130104802	0.0655929070039392	0.4360948200000010	F	F	F
0.0844511320084749	0.3989208939944362	0.4360888000000003	F	F	F
0.0826301130080012	0.7322489020043932	0.4360828100000020	F	F	F
0.8370211119890811	0.1602299059805716	0.3748038109999996	F	F	F
0.8352000710157768	0.4935579000078292	0.3747977909999989	F	F	F
0.8333781360114756	0.8268868919830368	0.3747918010000006	F	F	F
0.5037021039879122	0.1584088799887411	0.3726407890000019	F	F	F
0.5018801090123119	0.4917368889974725	0.3726347980000000	F	F	F
0.5000591280103066	0.8250648379894940	0.3726287779999993	F	F	F
0.1703821270029735	0.1565868999946147	0.3704777960000030	F	F	F

0.1685611460009753	0.4899158779871158	0.3704718050000011	F	F	F
0.1667401190103774	0.8232439160054312	0.3704658150000029	F	F	F
0.6731271740190010	0.3229198749868942	0.3590378170000008	F	F	F
0.6713061330003001	0.6562489269788117	0.3590317959999965	F	F	F
0.6694851519982947	0.9895768760162298	0.3590258059999982	F	F	F
0.3398081360094807	0.3210988939848889	0.3568748240000019	F	F	F
0.3379871249991311	0.6544268729852689	0.3568687739999987	F	F	F
0.3361651000151724	0.9877559539867704	0.3568627829999969	F	F	F
0.0064891129814413	0.3192778830199359	0.3547118010000005	F	F	F
0.0046671389799045	0.6526058909844963	0.3547058110000023	F	F	F
0.0028461179818962	0.9859338999932277	0.3546997899999980	F	F	F
0.9212151169991074	0.0815368740018627	0.2959358100000031	F	F	F
0.9193941349983419	0.4148648979920679	0.2959297900000024	F	F	F
0.9175731539963365	0.7481939200210306	0.2959237990000005	F	F	F
0.5878961680158810	0.0797158999912213	0.2937727870000018	F	F	F
0.5860751269971729	0.4130438570187636	0.2937667970000035	F	F	F
0.5842531320215656	0.7463728790023225	0.2937608060000017	F	F	F
0.7602561120186380	0.2375698979880525	0.2928088009999996	F	F	F
0.7584350709999299	0.5708978769884254	0.2928027809999989	F	F	F
0.7566142079884131	0.9042268990173881	0.2927967910000007	F	F	F
0.2545771300063606	0.0778948739993837	0.2916097940000029	F	F	F
0.2527551349853567	0.4112229049809457	0.2916038039999975	F	F	F
0.2509341240203966	0.7445508839813257	0.2915978130000028	F	F	F
0.4269371030187088	0.2357488869776958	0.2906458080000007	F	F	F
0.4251161220167035	0.5690768959864272	0.2906397880000000	F	F	F
0.4232951100075866	0.9024049039963842	0.2906337980000018	F	F	F
0.0936181169903705	0.2339268619937371	0.2884827849999994	F	F	F
0.0917971359883651	0.5672559139856546	0.2884767950000011	F	F	F
0.0899751189945306	0.9005838629776761	0.2884708050000029	F	F	F
0.9025256899812704	0.1076988015764234	0.6404551276791565	T	T	T
0.9010224387293458	0.4392479855932447	0.6402322780821709	T	T	T
0.8987325728483720	0.7723952501930769	0.6409070675725330	T	T	T
0.5642795552538363	0.1024386955957299	0.6416740107962218	T	T	T
0.5562159105467033	0.4392112809876534	0.6398211560266046	T	T	T
0.5594924199249410	0.7687124971873821	0.6420873173113365	T	T	T
0.2317395239504495	0.0998587834678484	0.6396624105143088	T	T	T

0.2280154945931969	0.4316765740189983	0.6396679666058083	T	T	T
0.2288177619685178	0.7638652044298899	0.6400879904130623	T	T	T
0.7859122279869462	0.2101423678760252	0.6377214328415527	T	T	T
0.7849575994031628	0.5425758321694548	0.6378844786834100	T	T	T
0.7820814107231954	0.8743081383897554	0.6383089451387681	T	T	T
0.4505459081866909	0.2109812816705428	0.6361418763126450	T	T	T
0.4478415395328673	0.5442816934538186	0.6332795436222820	T	T	T
0.4457941176962992	0.8778947179633296	0.6364572474878342	T	T	T
0.1198161765667109	0.2088584845258481	0.6346115400234277	T	T	T
0.1175168990807773	0.5448580273024406	0.6339531673389781	T	T	T
0.1150464384760455	0.8761111092320264	0.6351453847586996	T	T	T
0.8399031475863603	0.1506254721488776	0.5593694535754470	T	T	T
0.8383667483389922	0.4837371142162268	0.5594697969546480	T	T	T
0.8375328387370533	0.8174407797483927	0.5597759995681680	T	T	T
0.5050801583400523	0.1490504128347344	0.5597616443634525	T	T	T
0.5029247345367989	0.4822287083467781	0.5545991877719554	T	T	T
0.5020720847056445	0.8162708637429094	0.5599570370346627	T	T	T
0.1735575200967361	0.1474720574296831	0.5576708955666997	T	T	T
0.1705541715230856	0.4799530855389699	0.5566023414190523	T	T	T
0.1709674580154625	0.8146937715931890	0.5589018590836233	T	T	T
0.8684229307813683	0.0329177938319229	0.5616387379324862	T	T	T
0.8667099677471791	0.3656572816911448	0.5606611855168998	T	T	T
0.8659842186758808	0.6997084579418387	0.5601228330743501	T	T	T
0.5363543401136835	0.0316586409995154	0.5584069704732115	T	T	T
0.5354114306639220	0.3653371883630208	0.5567000171930404	T	T	T
0.5350405600370272	0.7001995696512761	0.5561410485425572	T	T	T
0.2035430734702353	0.0297675188187099	0.5560288292375670	T	T	T
0.2021947480722711	0.3638797255311021	0.5544015037386288	T	T	T
0.2007652340720764	0.6969368111774774	0.5545887164541392	T	T	T
0.9547235803835580	0.1125155478746258	0.5430905536738053	T	T	T
0.9527583957695877	0.4457781019092660	0.5430643127923309	T	T	T
0.9525864197878833	0.7800941840535947	0.5446616876092560	T	T	T
0.6208475989876282	0.1155724466575009	0.5473170241284130	T	T	T
0.6197817708037952	0.4497577371908976	0.5483970338273282	T	T	T
0.6195766503265215	0.7844073837321748	0.5499250690663970	T	T	T
0.7211099966991089	0.1816975587678646	0.5513044638443271	T	T	T

0.7201305751669699	0.5157450208492687	0.5506636011506731	T	T	T
0.7200126754275989	0.8506267039288292	0.5489092891837166	T	T	T
0.2889500844531080	0.1130754501251640	0.5454964921201217	T	T	T
0.2879453064612535	0.4476876815150263	0.5474806611995863	T	T	T
0.2862586690039793	0.7811336745993852	0.5470271763138254	T	T	T
0.8073968235897870	0.2636060704315625	0.5426464035359255	T	T	T
0.8063489546406054	0.5975322112449754	0.5433605097207803	T	T	T
0.8069835618094648	0.9319217731160466	0.5457131207876367	T	T	T
0.3878303312490868	0.1817817658065138	0.5483993103522056	T	T	T
0.3866260741768750	0.5161918804416251	0.5466726713631103	T	T	T
0.3853235438896844	0.8497274840103749	0.5473908893374593	T	T	T
0.4757800541471275	0.2626829233031007	0.5412298601789309	T	T	T
0.4751143287365874	0.5978069998790001	0.5422479143965672	T	T	T
0.4743761966008674	0.9302864737262641	0.5431178391751695	T	T	T
0.0558948211403548	0.1780440750552870	0.5481504606855141	T	T	T
0.0534391574392442	0.5117425636760415	0.5485645399276841	T	T	T
0.0536820528034067	0.8460164559431936	0.5471136569938142	T	T	T
0.1421974120428054	0.2607829232981051	0.5389842551096563	T	T	T
0.1412721789202683	0.5936759138805766	0.5385534662120895	T	T	T
0.1411047387385751	0.9282400983640385	0.5407684240091969	T	T	T
0.7836483948992077	0.0272322228320819	0.4862141339705155	T	T	T
0.7834222157140239	0.3592811437531270	0.4843675971966730	T	T	T
0.7818957684139690	0.6925538161857346	0.4842981220073522	T	T	T
0.9668625746947924	0.2088595932314695	0.4824027799226737	T	T	T
0.9652097107837074	0.5418215023794590	0.4820083230589198	T	T	T
0.9636334013525907	0.8747161362231960	0.4815901517757247	T	T	T
0.4499267146583842	0.0258471512686455	0.4844883432958330	T	T	T
0.4496507401430182	0.3577607929584607	0.4827224749757003	T	T	T
0.4482646784352559	0.6911143198657308	0.4825090889782246	T	T	T
0.6323066669646505	0.2092467076553042	0.4826170934760571	T	T	T
0.6306514515494057	0.5421264738185297	0.4822958494745367	T	T	T
0.6291393660078008	0.8749737025414144	0.4816761820171123	T	T	T
0.1164577737378217	0.0243430817772194	0.4827194486664740	T	T	T
0.1161817188152532	0.3559490756523330	0.4806375508716234	T	T	T
0.1147675048503354	0.6893268972115426	0.4805887825654965	T	T	T
0.2986252861453572	0.2070818422805586	0.4803850628839441	T	T	T

0.2966696731371131	0.5397229187791271	0.4799381127165206	T	T	T
0.2954929209687422	0.8730072903696154	0.4795673035628520	T	T	T
0.8903647406180828	0.3023984063845470	0.4727219841048385	T	T	T
0.8888248265669029	0.6357109279986305	0.4727884485279535	T	T	T
0.8883408314806368	0.9689822318356678	0.4734970422097423	T	T	T
0.5562341900538376	0.3014213981787751	0.4689482390086783	T	T	T
0.5549923974846432	0.6348875993641736	0.4691217152589133	T	T	T
0.5542498636433283	0.9679860099553517	0.4699141124597739	T	T	T
0.7003553934368525	0.1113949121198853	0.4673433840632128	T	T	T
0.6984874370048967	0.4436727049940467	0.4680528146745960	T	T	T
0.6965148782172845	0.7763896258386880	0.4683392143357247	T	T	T
0.2229418369395582	0.2995097124377590	0.4669619109204087	T	T	T
0.2216525236206485	0.6330361882439254	0.4670013500636717	T	T	T
0.2207612890628844	0.9662354976836618	0.4675077592400997	T	T	T
0.3673376136414440	0.1100334871817227	0.4649699244608447	T	T	T
0.3650310592110405	0.4418199160773822	0.4657200313519668	T	T	T
0.3637144666482526	0.7752052838479589	0.4661222439897481	T	T	T
0.0342291849173654	0.1093934054264146	0.4629265166268525	T	T	T
0.0326483279153015	0.4418987897060316	0.4636993842755826	T	T	T
0.0307521912075994	0.7747612212053075	0.4639093690204695	T	T	T
0.8040411469935194	0.1112479120019287	0.4128897789999968	F	F	F
0.8022201659915140	0.4445758760208278	0.4128837880000020	F	F	F
0.8003991839907485	0.7779049280127381	0.4128777979999967	F	F	F
0.8755741120097511	0.2052448690053339	0.4126187860000030	F	F	F
0.8737530709910430	0.5385729069782528	0.4126137789999973	F	F	F
0.8719320889902775	0.8719009159869842	0.4126077889999991	F	F	F
0.4707220790210442	0.1094268860100911	0.4107268149999967	F	F	F
0.4689011279819937	0.4427549239830100	0.4107207950000031	F	F	F
0.4670801160182734	0.7760828730204352	0.4107148049999978	F	F	F
0.5422551040085821	0.2034229039926885	0.4104558229999995	F	F	F
0.5404341220078166	0.5367518660049484	0.4104507859999984	F	F	F
0.5386121269868127	0.8700799349849788	0.4104447960000002	F	F	F
0.1374031309911814	0.1076049130073216	0.4085637929999990	F	F	F
0.1355821489904159	0.4409338830096985	0.4085578019999971	F	F	F
0.1337601240064572	0.7742619509909687	0.4085517820000035	F	F	F
0.2089361399984782	0.2016018780008579	0.4082927999999981	F	F	F

0.2071140999876491	0.5349298719827118	0.4082878230000020	F	F	F
0.2052931489940022	0.8682588930129072	0.4082817729999988	F	F	F
0.9701991080086358	0.2756438849854916	0.3967227939999987	F	F	F
0.9683781859791765	0.6089718939942230	0.3967168029999968	F	F	F
0.9665570859879296	0.9422998429862446	0.3967108129999986	F	F	F
0.7212521429830758	0.0278279130144128	0.3955617849999982	F	F	F
0.7194311020097715	0.3611558970086151	0.3955557940000034	F	F	F
0.7176091670054703	0.6944839359802941	0.3955498039999981	F	F	F
0.6368801000074669	0.2738219200182428	0.3945597709999973	F	F	F
0.6350591180067013	0.6071508530209115	0.3945537809999990	F	F	F
0.6332371229856975	0.9404789210021818	0.3945478199999997	F	F	F
0.3879331649902653	0.0260058919801196	0.3933988209999981	F	F	F
0.3861111099979553	0.3593349160066097	0.3933928009999974	F	F	F
0.3842900989875986	0.6926628950069897	0.3933868109999992	F	F	F
0.3035611509788367	0.2720008789995347	0.3923968080000009	F	F	F
0.3017391259948852	0.6053289180166175	0.3923907880000002	F	F	F
0.2999181449928798	0.9386578799834737	0.3923847969999983	F	F	F
0.0546131130071430	0.0241849160173402	0.3912357990000004	F	F	F
0.0527921389964945	0.3575128910226582	0.3912297789999997	F	F	F
0.0509711169997260	0.6908419130062171	0.3912237879999978	F	F	F
0.7851661440059274	0.1981308910132142	0.3429927829999997	F	F	F
0.7833451630039221	0.5314588549867167	0.3429867919999978	F	F	F
0.7815241219852211	0.8647879359882182	0.3429808019999996	F	F	F
0.4518471059964071	0.1963098939855712	0.3408298189999996	F	F	F
0.4500260949860504	0.5296378729859512	0.3408237700000001	F	F	F
0.4482041000104502	0.8629658819946755	0.3408178090000007	F	F	F
0.1185281349949463	0.1944879000087383	0.3386667970000019	F	F	F
0.1167061180011117	0.5278168919839459	0.3386608060000000	F	F	F
0.1148851360003462	0.8611448999939100	0.3386547859999993	F	F	F
0.8824861050079207	0.1266008759859929	0.3339448270000034	F	F	F
0.8806651230071552	0.4599289000215947	0.3339387770000002	F	F	F
0.8788440819884471	0.7932578329788669	0.3339327870000020	F	F	F
0.9598761199854096	0.0364938789993445	0.3337728079999991	F	F	F
0.9580550790120981	0.3698219059858445	0.3337678019999970	F	F	F
0.9562330839910942	0.7031508679981044	0.3337617809999998	F	F	F
0.5491670970067517	0.1247799100108651	0.3317818049999985	F	F	F

0.5473461750226889	0.4581078590028866	0.3317758139999967	F	F	F
0.5455251340039808	0.7914358970212021	0.3317697940000031	F	F	F
0.6265561579819448	0.0346729049887031	0.3316098150000002	F	F	F
0.6247351170086333	0.3680008650125401	0.3316038250000020	F	F	F
0.6229141350078677	0.7013288740212644	0.3315988180000033	F	F	F
0.2158481329966477	0.1229578849815098	0.3296188119999997	F	F	F
0.2140271070048172	0.4562869070104725	0.3296128209999978	F	F	F
0.2122051419921647	0.7896149160192039	0.3296067710000017	F	F	F
0.2932371499807758	0.0328508839998065	0.3294467930000025	F	F	F
0.2914161090074643	0.3661799130201260	0.3294408020000006	F	F	F
0.2895951570150501	0.6995078920204989	0.3294357959999985	F	F	F
0.7125151159940089	0.2879979009914209	0.3141148090000030	F	F	F
0.7106941339932362	0.6213259100001522	0.3141087890000023	F	F	F
0.7088730930199318	0.9546548720124051	0.3141027989999969	F	F	F
0.3791961069940726	0.2861769199894226	0.3119518159999970	F	F	F
0.3773751259920672	0.6195048689814442	0.3119457960000034	F	F	F
0.3755531310164670	0.9528329370081110	0.3119398059999980	F	F	F
0.0458771439827359	0.2843548950054640	0.3097878100000031	F	F	F
0.0440551229938393	0.6176839469973814	0.3097828029999974	F	F	F
0.0422341449881287	0.9510118959894029	0.3097768129999992	F	F	F
0.8732610939964118	0.0398549059864806	0.2601577940000013	F	F	F
0.8714401720123490	0.3731828630140228	0.2601518029999994	F	F	F
0.8696190710223348	0.7065109009869417	0.2601457829999987	F	F	F
0.5399420859952428	0.0380328859963512	0.2579948010000024	F	F	F
0.5381201509909488	0.3713619110216086	0.2579888109999970	F	F	F
0.5362991090188771	0.7046898600136302	0.2579828200000023	F	F	F
0.8128991129996024	0.2660319209917859	0.2561918199999980	F	F	F
0.8110781309988297	0.5993608830040458	0.2561857999999972	F	F	F
0.8092561359778330	0.9326888920127701	0.2561797799999965	F	F	F
0.2066221390186556	0.0362119119857027	0.2558318080000035	F	F	F
0.2048011119826612	0.3695398869910207	0.2558258179999982	F	F	F
0.2029801460075333	0.7028688790116249	0.2558197979999974	F	F	F
0.4795800749900820	0.2642108800184815	0.2540287969999966	F	F	F
0.4777581389870207	0.5975399020020404	0.2540228069999984	F	F	F
0.4759371280220606	0.9308679100120045	0.2540167869999976	F	F	F
0.7232301240073369	0.1896398960131407	0.2532247899999973	F	F	F

0.7214091420065714	0.5229678750135207	0.2532187999999991	F	F	F
0.7195871469855675	0.8562968369803770	0.2532128100000008	F	F	F
0.9609541890203559	0.1165518979961959	0.2520078120000022	F	F	F
0.9591330889837053	0.4498798549783416	0.2520017920000015	F	F	F
0.9573121669996425	0.7832078929966571	0.2519958020000033	F	F	F
0.1462601129866172	0.2623898980177088	0.2518658039999977	F	F	F
0.1444391460127221	0.5957179069810365	0.2518597839999970	F	F	F
0.1426181350023725	0.9290469290099992	0.2518538240000012	F	F	F
0.3899110849990564	0.1878188850027840	0.2510617969999984	F	F	F
0.3880891499947552	0.5211468940115154	0.2510558070000002	F	F	F
0.3862681090214437	0.8544749020214795	0.2510498170000020	F	F	F
0.6276350619845417	0.1147298810023614	0.2498448039999985	F	F	F
0.6258141400004789	0.4480589029859274	0.2498387839999978	F	F	F
0.6239920850081688	0.7813869119946588	0.2498328089999973	F	F	F
0.0565911229955915	0.1859969050086576	0.2488987890000018	F	F	F
0.0547701489849430	0.5193258519940400	0.2488927989999965	F	F	F
0.0529491269881746	0.8526539210194741	0.2488867940000006	F	F	F
0.2943161430096666	0.1129089000003631	0.2476818109999996	F	F	F
0.2924941479886627	0.4462378920209744	0.2476758059999966	F	F	F
0.2906731369783060	0.7795658710213473	0.2476697860000030	F	F	F
0.9231250803448250	0.7414038095898434	0.6581054688122815	T	T	T
0.5827413233922167	0.0679176866978328	0.6593554943741072	T	T	T
0.2425203197200808	0.7266194355179736	0.6568639434508790	T	T	T
0.7478492168860552	0.2233653122631480	0.6532871447577461	T	T	T
0.0835714650200242	0.2270682318671883	0.6496197713438018	T	T	T
0.0777388971510984	0.5470950675011600	0.6503737307799624	T	T	T
0.9288206432761253	0.0783278940695476	0.6576941152111954	T	T	T
0.9210069137461688	0.4056504758301560	0.6580366127532740	T	T	T
0.5752397118845090	0.4072626815800632	0.6605400613961245	T	T	T
0.5903611244557762	0.7436959944150483	0.6587394785097569	T	T	T
0.2527127494336667	0.0665896192769404	0.6570264701017421	T	T	T
0.2678881732614734	0.4292856680426301	0.6559003790395063	T	T	T
0.7504854695956213	0.5635303170247197	0.6533530535843625	T	T	T
0.7478330557722727	0.8961374251612861	0.6532354111685557	T	T	T
0.4132626691938509	0.2273952280787565	0.6508139806459208	T	T	T
0.4057801705047085	0.8856514045068602	0.6510383955167748	T	T	T

0.0762061058274133	0.8886258159403204	0.6496247030515921	T	T	T
0.5003200770105281	0.3033929169825811	0.2606458070000031	F	F	F
0.4984991549810687	0.6367208959829540	0.2606397870000023	F	F	F
0.4966780839994058	0.9700488450203792	0.2606337960000005	F	F	F
0.9957210419885669	0.1439058930061066	0.2590687869999968	F	F	F
0.9939001800212068	0.4772338869879604	0.2590627969999986	F	F	F
0.9920781249835002	0.8105618359799820	0.2590568070000003	F	F	F
0.1670011130004241	0.3015708919986224	0.2584828139999971	F	F	F
0.1651801469798997	0.6348999139821885	0.2584767940000035	F	F	F
0.1633591199893019	0.9682278630196066	0.2584708030000016	F	F	F
0.6624021530220432	0.1420838830036288	0.2569057939999979	F	F	F
0.6605801580010393	0.4754129049871949	0.2568998039999997	F	F	F
0.6587591169823312	0.8087408539792165	0.2568938140000014	F	F	F
0.3290821310018757	0.1402629020016306	0.2547428009999990	F	F	F
0.3272611199915190	0.4735908810020035	0.2547368110000008	F	F	F
0.3254401389895136	0.8069198730226148	0.2547307910000001	F	F	F
0.8734241129859370	0.0502678940068506	0.2223587929999979	F	F	F
0.8716031309851715	0.3835968970138808	0.2223527880000020	F	F	F
0.8697810769916288	0.7169249060226051	0.2223467979999967	F	F	F
0.5401051640027106	0.0484468750063769	0.2201958149999967	F	F	F
0.5382831100091678	0.3817759160118754	0.2201897950000031	F	F	F
0.5364621280084023	0.7151039240218395	0.2201837900000001	F	F	F
0.2067850979914780	0.0466258970006734	0.2180327920000025	F	F	F
0.2049641310175829	0.3799538610195654	0.2180267869999994	F	F	F
0.2031431200072333	0.7132828830031315	0.2180207970000012	F	F	F
0.7250071169868377	0.2004988790164575	0.2167577889999990	F	F	F
0.7231860760135334	0.5338268879797852	0.2167517990000007	F	F	F
0.7213641410092322	0.8671548959897493	0.2167457939999977	F	F	F
0.3916881680036113	0.1986769140038120	0.2145947960000001	F	F	F
0.3898661140100685	0.5320059059790196	0.2145887909999971	F	F	F
0.3880451620176544	0.8653339149877510	0.2145828009999988	F	F	F
0.0583681089837356	0.1968558880119744	0.2124317880000035	F	F	F
0.0547261130217152	0.8635128740144395	0.2124197930000022	F	F	F
0.0565471309780250	0.5301839110034194	0.2124257979999982	F	F	F
0.8336401580032344	0.3052138690203918	0.2628088000000020	F	F	F
0.8318190570132202	0.6385419370016621	0.2628027800000012	F	F	F

0.8299971220089262	0.9718708990139220	0.2627967889999994	F	F	F
0.3510479901622202	0.5183449270474446	0.7056974405196328	T	T	T
0.4816839438272198	0.4986326266682048	0.7380801267125263	T	T	T
0.4061737008223573	0.4717463041142898	0.7381070175282252	T	T	T
0.4203559079781032	0.6460047442160122	0.7697063477805243	T	T	T
0.4078144518991464	0.5622829899531435	0.7826656453992863	T	T	T
0.4260266303806385	0.5365227514020997	0.7052830348253246	T	T	T
0.4197093370066661	0.6002934196665832	0.7532121626256246	T	T	T

:::::::::::::

XVIII (VASP)

:::::::::::::

E(PBE, VASP) = -2547.28122101 (eV)

Edisp /kcal,au,eV: -596.8464 -0.95113521 -25.8817070

header

1.00000000000000000

22.0272006988999998	0.0000000000000000	0.0000000000000000
0.0000000000000000	22.0272006988999998	0.0000000000000000
0.0000000000000000	0.0000000000000000	25.0000000000000000

Si	O	H	W
90	207	58	1

Selective dynamics

Direct

0.8921822677578017	0.1017755701247502	0.5759595537144446	T	T	T
0.8904903070351710	0.4343133251581658	0.5758030869632149	T	T	T
0.8893870354694557	0.7682598651519910	0.5762782041267801	T	T	T
0.5570262206367423	0.1002133593782177	0.5765833557848763	T	T	T
0.5554940547325775	0.4338649950975372	0.5744568291828477	T	T	T
0.5547202714854189	0.7682595975153750	0.5769433367503544	T	T	T
0.2246749056226736	0.0982406956397282	0.5746237043772969	T	T	T
0.2233893128949554	0.4318462474283695	0.5747062996508079	T	T	T
0.2219908981834503	0.7647240988371011	0.5752014949801246	T	T	T
0.7886120707484976	0.2015985869842442	0.5727728480046747	T	T	T
0.7877998723888817	0.5350814202971784	0.5727100276656899	T	T	T
0.7865914601522686	0.8688909195742587	0.5732463757146972	T	T	T

0.4545805426878705	0.2012653610877699	0.5712864530141017	T	T	T
0.4543217848111782	0.5343414690575217	0.5684977946438593	T	T	T
0.4517484766291978	0.8687345475962359	0.5717867486333589	T	T	T
0.1227536362758962	0.1997523087936750	0.5699545337119725	T	T	T
0.1213605046387670	0.5327128004549291	0.5695191315240108	T	T	T
0.1199623221361955	0.8666429825290292	0.5705529352328594	T	T	T
0.8374705106632663	0.3228409764160208	0.5147379898726508	T	T	T
0.8362682945184982	0.6566839854313115	0.5147748307252558	T	T	T
0.8370942499742823	0.9903695662939324	0.5166583719829086	T	T	T
0.0038111109089826	0.1523563463423565	0.5086295050298255	T	T	T
0.0020008240194299	0.4854377797329206	0.5086739080482783	T	T	T
0.0008047466526163	0.8191417726007727	0.5088696483465542	T	T	T
0.5047033564292683	0.3216359394301140	0.5120356206688367	T	T	T
0.5036197550616377	0.6564367516462959	0.5125751652299844	T	T	T
0.5040486457130270	0.9890300082414973	0.5139820999234779	T	T	T
0.6692304771671553	0.1547241174687946	0.5119286376875887	T	T	T
0.6682752299966379	0.4883361909252157	0.5118118412629790	T	T	T
0.6668159866295851	0.8220196916157911	0.5121002197086766	T	T	T
0.1716696065765526	0.3204029031471658	0.5100883491091331	T	T	T
0.1699724495554732	0.6536356065953053	0.5101402275890831	T	T	T
0.1706948366022663	0.9872193799276101	0.5118670080429212	T	T	T
0.3360928550125445	0.1530946210565192	0.5097677586479172	T	T	T
0.3344808962045123	0.4865189486281180	0.5098292389256597	T	T	T
0.3331381171374644	0.8200610022430449	0.5101255791138791	T	T	T
0.9252931479857907	0.2479518950073754	0.4412057999999988	F	F	F
0.9234721060137190	0.5812809469992928	0.4411998089999969	F	F	F
0.9216511250117136	0.9146088959913143	0.4411938189999987	F	F	F
0.7529111499777699	0.0692349000150614	0.4404208059999988	F	F	F
0.7510890959842271	0.4025638999803931	0.4404148160000005	F	F	F
0.7492681149822289	0.7358919379987086	0.4404087959999998	F	F	F
0.5919740800133155	0.2461308839970187	0.4390428069999999	F	F	F
0.5901531579838561	0.5794588920069828	0.4390367869999992	F	F	F
0.5883311029915461	0.9127878550180029	0.4390307959999973	F	F	F
0.4195911590101247	0.0674138810145806	0.4382577840000010	F	F	F
0.4177701179914166	0.4007419050047858	0.4382517929999992	F	F	F
0.4159491659989953	0.7340708370086944	0.4382458030000009	F	F	F

0.2586551010217377	0.2443099019962531	0.4368798140000010	F	F	F
0.2568331360090852	0.5776379110049774	0.4368738229999991	F	F	F
0.2550121249987285	0.9109659190149415	0.4368678029999984	F	F	F
0.0862721130104802	0.0655929070039392	0.4360948200000010	F	F	F
0.0844511320084749	0.3989208939944362	0.4360888000000003	F	F	F
0.0826301130080012	0.7322489020043932	0.4360828100000020	F	F	F
0.8370211119890811	0.1602299059805716	0.3748038109999996	F	F	F
0.8352000710157768	0.4935579000078292	0.3747977909999989	F	F	F
0.8333781360114756	0.8268868919830368	0.3747918010000006	F	F	F
0.5037021039879122	0.1584088799887411	0.3726407890000019	F	F	F
0.5018801090123119	0.4917368889974725	0.3726347980000000	F	F	F
0.5000591280103066	0.8250648379894940	0.3726287779999993	F	F	F
0.1703821270029735	0.1565868999946147	0.3704777960000030	F	F	F
0.1685611460009753	0.4899158779871158	0.3704718050000011	F	F	F
0.1667401190103774	0.8232439160054312	0.3704658150000029	F	F	F
0.6731271740190010	0.3229198749868942	0.3590378170000008	F	F	F
0.6713061330003001	0.6562489269788117	0.3590317959999965	F	F	F
0.6694851519982947	0.9895768760162298	0.3590258059999982	F	F	F
0.3398081360094807	0.3210988939848889	0.3568748240000019	F	F	F
0.3379871249991311	0.6544268729852689	0.3568687739999987	F	F	F
0.3361651000151724	0.9877559539867704	0.3568627829999969	F	F	F
0.0064891129814413	0.3192778830199359	0.3547118010000005	F	F	F
0.0046671389799045	0.6526058909844963	0.3547058110000023	F	F	F
0.0028461179818962	0.9859338999932277	0.3546997899999980	F	F	F
0.9212151169991074	0.0815368740018627	0.2959358100000031	F	F	F
0.9193941349983419	0.4148648979920679	0.2959297900000024	F	F	F
0.9175731539963365	0.7481939200210306	0.2959237990000005	F	F	F
0.5878961680158810	0.0797158999912213	0.2937727870000018	F	F	F
0.5860751269971729	0.4130438570187636	0.2937667970000035	F	F	F
0.5842531320215656	0.7463728790023225	0.2937608060000017	F	F	F
0.7602561120186380	0.2375698979880525	0.2928088009999996	F	F	F
0.7584350709999299	0.5708978769884254	0.2928027809999989	F	F	F
0.7566142079884131	0.9042268990173881	0.2927967910000007	F	F	F
0.2545771300063606	0.0778948739993837	0.2916097940000029	F	F	F
0.2527551349853567	0.4112229049809457	0.2916038039999975	F	F	F
0.2509341240203966	0.7445508839813257	0.2915978130000028	F	F	F

0.4269371030187088	0.2357488869776958	0.2906458080000007	F	F	F
0.4251161220167035	0.5690768959864272	0.2906397880000000	F	F	F
0.4232951100075866	0.9024049039963842	0.2906337980000018	F	F	F
0.0936181169903705	0.2339268619937371	0.2884827849999994	F	F	F
0.0917971359883651	0.5672559139856546	0.2884767950000011	F	F	F
0.0899751189945306	0.9005838629776761	0.2884708050000029	F	F	F
0.9024462260788810	0.1076669511942153	0.6405743786339756	T	T	T
0.9005283198631157	0.4395194919963090	0.6404726533889009	T	T	T
0.8985329933022239	0.7726166531681136	0.6410964254279329	T	T	T
0.5641969537033020	0.1029046588352598	0.6416473631110117	T	T	T
0.5568738048672620	0.4404693792821164	0.6395931634765375	T	T	T
0.5596030320372953	0.7690108924291732	0.6421664408265436	T	T	T
0.2316085920685615	0.1002420792581267	0.6397231706845206	T	T	T
0.2272084955862173	0.4330006474437553	0.6398242815493451	T	T	T
0.2283797212035003	0.7647071086228306	0.6403389588574876	T	T	T
0.7860433658249790	0.2097224910486829	0.6377866899659739	T	T	T
0.7845847541325620	0.5417754575657012	0.6378148547263356	T	T	T
0.7817989246072329	0.8741678561740305	0.6383854421251901	T	T	T
0.4513825672121220	0.2107081950393632	0.6360767672619644	T	T	T
0.4489433705314000	0.5411046138067135	0.6344166438946972	T	T	T
0.4462036388910412	0.8772801830134404	0.6365684785692967	T	T	T
0.1194996125314544	0.2095159876064965	0.6347533624502956	T	T	T
0.1181328731681077	0.5428107665798078	0.6342695040000925	T	T	T
0.1148512225343567	0.8761315934333105	0.6353076148068584	T	T	T
0.8406759199737933	0.1513670196185323	0.5591522336258365	T	T	T
0.8391634267215811	0.4842769891657054	0.5591340029929286	T	T	T
0.8382117888874056	0.8182655746746506	0.5596771050540824	T	T	T
0.5053537340810007	0.1493311047527558	0.5594856915247937	T	T	T
0.5042795218367109	0.4825739281351933	0.5544064966315867	T	T	T
0.5027525567627091	0.8169553185362814	0.5598768110698110	T	T	T
0.1740467382467863	0.1483812966283981	0.5576895024517409	T	T	T
0.1725641940847097	0.4814299786596014	0.5566751404940049	T	T	T
0.1712833753816562	0.8151755525231900	0.5590918754275255	T	T	T
0.8688676704384518	0.0334338441443964	0.5613057875155496	T	T	T
0.8674080266664617	0.3662564753783311	0.5602380352011253	T	T	T
0.8663884525197685	0.7004307471742417	0.5598119039313075	T	T	T

0.5367661178468427	0.0321162186891379	0.5581434075115331	T	T	T
0.5362534975123950	0.3655306318070485	0.5565944280556930	T	T	T
0.5356048041183302	0.7009630743656808	0.5557389514509080	T	T	T
0.2036419672731132	0.0304863258730438	0.5557939585781159	T	T	T
0.2037504139349421	0.3646563653582452	0.5536997420433122	T	T	T
0.2014604221047382	0.6978010589197473	0.5541975023897215	T	T	T
0.9554106538141022	0.1131068158248978	0.5436098586706776	T	T	T
0.9538354830643143	0.4464492706503086	0.5439560820041511	T	T	T
0.9532396568194755	0.7806818416208484	0.5452601451003452	T	T	T
0.6213309251775695	0.1159009176131351	0.5475118334402631	T	T	T
0.6213953209472981	0.4494774052264343	0.5486361878296809	T	T	T
0.6201729484047027	0.7850410340087631	0.5503773830386991	T	T	T
0.7216915293521107	0.1819704823213164	0.5508974734456278	T	T	T
0.7210879384151110	0.5163569789928607	0.5496903799701830	T	T	T
0.7205608691026952	0.8509516784164292	0.5484695322267286	T	T	T
0.2893247966040490	0.1134614206519839	0.5457791670172850	T	T	T
0.2892769066571840	0.4483391776957473	0.5482348131187083	T	T	T
0.2869376235011224	0.7816408806655506	0.5475872700797418	T	T	T
0.8075795076545946	0.2643214767941136	0.5432181455571307	T	T	T
0.8070646483135474	0.5982062229351646	0.5440544882310425	T	T	T
0.8071806498238462	0.9325886529596275	0.5463312241393894	T	T	T
0.3882188676289489	0.1821354687874116	0.5481076415909740	T	T	T
0.3878277301651458	0.5167585422949679	0.5463188920214562	T	T	T
0.3859454708293994	0.8502753512242008	0.5469233925810449	T	T	T
0.4757749868618861	0.2631579466473089	0.5413043669219476	T	T	T
0.4757704930666980	0.5987835381403842	0.5448811797769048	T	T	T
0.4746388482399966	0.9308614833532820	0.5434758114981005	T	T	T
0.0564397196317092	0.1789875722175730	0.5477507691119075	T	T	T
0.0549493718104651	0.5119598934161161	0.5474787177433993	T	T	T
0.0541919140051996	0.8465782755496402	0.5466825243552426	T	T	T
0.1431070093982859	0.2618262038426231	0.5398132754099634	T	T	T
0.1414391862929935	0.5948350869916504	0.5394501228524006	T	T	T
0.1415450567959074	0.9287872464835519	0.5412851088458155	T	T	T
0.7836308088526270	0.0273424842105246	0.4862949193325727	T	T	T
0.7833060230151105	0.3595350351933929	0.4845930920917564	T	T	T
0.7819149671382570	0.6925535033114451	0.4843125150188352	T	T	T

0.9669132479449246	0.2089623406136648	0.4823904926203101	T	T	T
0.9652587272373715	0.5418616787959083	0.4820629412508783	T	T	T
0.9636647467153757	0.8747295931721466	0.4815466064462009	T	T	T
0.4499777981130475	0.0259662169825675	0.4845156962900951	T	T	T
0.4498725235210853	0.3583512200267552	0.4830050145325582	T	T	T
0.4480532757581606	0.6906439184950695	0.4825694854370203	T	T	T
0.6323559152860498	0.2094809701254599	0.4827464082850056	T	T	T
0.6306214746544386	0.5421697542493548	0.4823782496023130	T	T	T
0.6292235534576871	0.8750420390461201	0.4816308224753770	T	T	T
0.1164958229052849	0.0243059575325558	0.4826479380060891	T	T	T
0.1163765654270337	0.3563398957022975	0.4808528226651496	T	T	T
0.1149457424850016	0.6894794142130277	0.4805519082316347	T	T	T
0.2986498775322436	0.2072077864726512	0.4804374403779801	T	T	T
0.2969495631003927	0.5398968624737833	0.4799654624523743	T	T	T
0.2955352742588008	0.8729647230698820	0.4794822547701541	T	T	T
0.8901750529808099	0.3023956244367155	0.4725818928131005	T	T	T
0.8888813516696616	0.6358013446209106	0.4727264370750641	T	T	T
0.8882253517569294	0.9689474931455223	0.4734577589255652	T	T	T
0.5561673107276383	0.3015391215627022	0.4687824059454790	T	T	T
0.5543830900732858	0.6341904752658303	0.4695274338539778	T	T	T
0.5542502989130881	0.9680446393441579	0.4698310018803682	T	T	T
0.7003104623357688	0.1115033466466573	0.4671987529288363	T	T	T
0.6983841812060242	0.4439186084591608	0.4674856683840108	T	T	T
0.6964586504739571	0.7763550822527084	0.4683094939691361	T	T	T
0.2228788839728955	0.2996261351348946	0.4666124019224814	T	T	T
0.2217213386973926	0.6329843354032866	0.4670500951333532	T	T	T
0.2208862278306792	0.9662454037998109	0.4675827939482718	T	T	T
0.3673145440030017	0.1100712035645023	0.4649544944545959	T	T	T
0.3652864444078145	0.4421110884592893	0.4657547210770269	T	T	T
0.3638328394392978	0.7751480987069390	0.4662478125868920	T	T	T
0.0343584341973332	0.1094710055322133	0.4630693155356703	T	T	T
0.0323742070956555	0.4417475799022611	0.4634823815569420	T	T	T
0.0307375192507283	0.7746872191964663	0.4639816541494153	T	T	T
0.8040411469935194	0.1112479120019287	0.4128897789999968	F	F	F
0.8022201659915140	0.4445758760208278	0.4128837880000020	F	F	F
0.8003991839907485	0.7779049280127381	0.4128777979999967	F	F	F

0.8755741120097511	0.2052448690053339	0.4126187860000030	F	F	F
0.8737530709910430	0.5385729069782528	0.4126137789999973	F	F	F
0.8719320889902775	0.8719009159869842	0.4126077889999991	F	F	F
0.4707220790210442	0.1094268860100911	0.4107268149999967	F	F	F
0.4689011279819937	0.4427549239830100	0.4107207950000031	F	F	F
0.4670801160182734	0.7760828730204352	0.4107148049999978	F	F	F
0.5422551040085821	0.2034229039926885	0.4104558229999995	F	F	F
0.5404341220078166	0.5367518660049484	0.4104507859999984	F	F	F
0.5386121269868127	0.8700799349849788	0.4104447960000002	F	F	F
0.1374031309911814	0.1076049130073216	0.4085637929999990	F	F	F
0.1355821489904159	0.4409338830096985	0.4085578019999971	F	F	F
0.1337601240064572	0.7742619509909687	0.4085517820000035	F	F	F
0.2089361399984782	0.2016018780008579	0.4082927999999981	F	F	F
0.2071140999876491	0.5349298719827118	0.4082878230000020	F	F	F
0.2052931489940022	0.8682588930129072	0.4082817729999988	F	F	F
0.9701991080086358	0.2756438849854916	0.3967227939999987	F	F	F
0.9683781859791765	0.6089718939942230	0.3967168029999968	F	F	F
0.9665570859879296	0.9422998429862446	0.3967108129999986	F	F	F
0.7212521429830758	0.0278279130144128	0.3955617849999982	F	F	F
0.7194311020097715	0.3611558970086151	0.3955557940000034	F	F	F
0.7176091670054703	0.6944839359802941	0.3955498039999981	F	F	F
0.6368801000074669	0.2738219200182428	0.3945597709999973	F	F	F
0.6350591180067013	0.6071508530209115	0.3945537809999990	F	F	F
0.6332371229856975	0.9404789210021818	0.3945478199999997	F	F	F
0.3879331649902653	0.0260058919801196	0.3933988209999981	F	F	F
0.3861111099979553	0.3593349160066097	0.3933928009999974	F	F	F
0.3842900989875986	0.6926628950069897	0.3933868109999992	F	F	F
0.3035611509788367	0.2720008789995347	0.3923968080000009	F	F	F
0.3017391259948852	0.6053289180166175	0.3923907880000002	F	F	F
0.2999181449928798	0.9386578799834737	0.3923847969999983	F	F	F
0.0546131130071430	0.0241849160173402	0.3912357990000004	F	F	F
0.0527921389964945	0.3575128910226582	0.3912297789999997	F	F	F
0.0509711169997260	0.6908419130062171	0.3912237879999978	F	F	F
0.7851661440059274	0.1981308910132142	0.3429927829999997	F	F	F
0.7833451630039221	0.5314588549867167	0.3429867919999978	F	F	F
0.7815241219852211	0.8647879359882182	0.3429808019999996	F	F	F

0.4518471059964071	0.1963098939855712	0.3408298189999996	F	F	F
0.4500260949860504	0.5296378729859512	0.3408237700000001	F	F	F
0.4482041000104502	0.8629658819946755	0.3408178090000007	F	F	F
0.1185281349949463	0.1944879000087383	0.3386667970000019	F	F	F
0.1167061180011117	0.5278168919839459	0.3386608060000000	F	F	F
0.1148851360003462	0.8611448999939100	0.3386547859999993	F	F	F
0.8824861050079207	0.1266008759859929	0.3339448270000034	F	F	F
0.8806651230071552	0.4599289000215947	0.3339387770000002	F	F	F
0.8788440819884471	0.7932578329788669	0.3339327870000020	F	F	F
0.9598761199854096	0.0364938789993445	0.3337728079999991	F	F	F
0.9580550790120981	0.3698219059858445	0.3337678019999970	F	F	F
0.9562330839910942	0.7031508679981044	0.3337617809999998	F	F	F
0.5491670970067517	0.1247799100108651	0.3317818049999985	F	F	F
0.5473461750226889	0.4581078590028866	0.3317758139999967	F	F	F
0.5455251340039808	0.7914358970212021	0.3317697940000031	F	F	F
0.6265561579819448	0.0346729049887031	0.3316098150000002	F	F	F
0.6247351170086333	0.3680008650125401	0.3316038250000020	F	F	F
0.6229141350078677	0.7013288740212644	0.3315988180000033	F	F	F
0.2158481329966477	0.1229578849815098	0.3296188119999997	F	F	F
0.2140271070048172	0.4562869070104725	0.3296128209999978	F	F	F
0.2122051419921647	0.7896149160192039	0.3296067710000017	F	F	F
0.2932371499807758	0.0328508839998065	0.3294467930000025	F	F	F
0.2914161090074643	0.3661799130201260	0.3294408020000006	F	F	F
0.2895951570150501	0.6995078920204989	0.3294357959999985	F	F	F
0.7125151159940089	0.2879979009914209	0.3141148090000030	F	F	F
0.7106941339932362	0.6213259100001522	0.3141087890000023	F	F	F
0.7088730930199318	0.9546548720124051	0.3141027989999969	F	F	F
0.3791961069940726	0.2861769199894226	0.3119518159999970	F	F	F
0.3773751259920672	0.6195048689814442	0.3119457960000034	F	F	F
0.3755531310164670	0.9528329370081110	0.3119398059999980	F	F	F
0.0458771439827359	0.2843548950054640	0.3097878100000031	F	F	F
0.0440551229938393	0.6176839469973814	0.3097828029999974	F	F	F
0.0422341449881287	0.9510118959894029	0.3097768129999992	F	F	F
0.8732610939964118	0.0398549059864806	0.2601577940000013	F	F	F
0.8714401720123490	0.3731828630140228	0.2601518029999994	F	F	F
0.8696190710223348	0.7065109009869417	0.2601457829999987	F	F	F

0.5399420859952428	0.0380328859963512	0.2579948010000024	F	F	F
0.5381201509909488	0.3713619110216086	0.2579888109999970	F	F	F
0.5362991090188771	0.7046898600136302	0.2579828200000023	F	F	F
0.8128991129996024	0.2660319209917859	0.2561918199999980	F	F	F
0.8110781309988297	0.5993608830040458	0.2561857999999972	F	F	F
0.8092561359778330	0.9326888920127701	0.2561797799999965	F	F	F
0.2066221390186556	0.0362119119857027	0.2558318080000035	F	F	F
0.2048011119826612	0.3695398869910207	0.2558258179999982	F	F	F
0.2029801460075333	0.7028688790116249	0.2558197979999974	F	F	F
0.4795800749900820	0.2642108800184815	0.2540287969999966	F	F	F
0.4777581389870207	0.5975399020020404	0.2540228069999984	F	F	F
0.4759371280220606	0.9308679100120045	0.2540167869999976	F	F	F
0.7232301240073369	0.1896398960131407	0.2532247899999973	F	F	F
0.7214091420065714	0.5229678750135207	0.2532187999999991	F	F	F
0.7195871469855675	0.8562968369803770	0.2532128100000008	F	F	F
0.9609541890203559	0.1165518979961959	0.2520078120000022	F	F	F
0.9591330889837053	0.4498798549783416	0.2520017920000015	F	F	F
0.9573121669996425	0.7832078929966571	0.2519958020000033	F	F	F
0.1462601129866172	0.2623898980177088	0.2518658039999977	F	F	F
0.1444391460127221	0.5957179069810365	0.2518597839999970	F	F	F
0.1426181350023725	0.9290469290099992	0.2518538240000012	F	F	F
0.3899110849990564	0.1878188850027840	0.2510617969999984	F	F	F
0.3880891499947552	0.5211468940115154	0.2510558070000002	F	F	F
0.3862681090214437	0.8544749020214795	0.2510498170000020	F	F	F
0.6276350619845417	0.1147298810023614	0.2498448039999985	F	F	F
0.6258141400004789	0.4480589029859274	0.2498387839999978	F	F	F
0.6239920850081688	0.7813869119946588	0.2498328089999973	F	F	F
0.0565911229955915	0.1859969050086576	0.2488987890000018	F	F	F
0.0547701489849430	0.5193258519940400	0.2488927989999965	F	F	F
0.0529491269881746	0.8526539210194741	0.2488867940000006	F	F	F
0.2943161430096666	0.1129089000003631	0.2476818109999996	F	F	F
0.2924941479886627	0.4462378920209744	0.2476758059999966	F	F	F
0.2906731369783060	0.7795658710213473	0.2476697860000030	F	F	F
0.9211210265692299	0.7405051416627274	0.6586164162338191	T	T	T
0.5843941170304824	0.0693301311842976	0.6592859654897991	T	T	T
0.2486745125069590	0.7306213079538098	0.6571479974297855	T	T	T

0.7481021587157990	0.2233404866995163	0.6533265023960275	T	T	T
0.0814413907909989	0.2234803397380195	0.6498005988505688	T	T	T
0.0787912118351946	0.5515446678962116	0.6497969875292771	T	T	T
0.9247843758817359	0.0756199307980843	0.6584392202928760	T	T	T
0.9208634695345476	0.4062473806955631	0.6584309643046110	T	T	T
0.5813588099424444	0.4131464093291005	0.6609722247545303	T	T	T
0.5868118832392510	0.7404700965952629	0.6593648614720395	T	T	T
0.2526724062568542	0.0670402648522581	0.6571310363527644	T	T	T
0.2596742443289326	0.4115637334156232	0.6579740247496857	T	T	T
0.7497587248215223	0.5623883493013904	0.6530564139401203	T	T	T
0.7458073447853790	0.8929862844388488	0.6533102045238119	T	T	T
0.4166033820943912	0.2318041977930649	0.6508628264723597	T	T	T
0.4069732723994776	0.8883984621185013	0.6511194447236850	T	T	T
0.0759705293927464	0.8887316112384638	0.6496589716514253	T	T	T
0.5003200770105281	0.3033929169825811	0.2606458070000031	F	F	F
0.4984991549810687	0.6367208959829540	0.2606397870000023	F	F	F
0.4966780839994058	0.9700488450203792	0.2606337960000005	F	F	F
0.9957210419885669	0.1439058930061066	0.2590687869999968	F	F	F
0.9939001800212068	0.4772338869879604	0.2590627969999986	F	F	F
0.9920781249835002	0.8105618359799820	0.2590568070000003	F	F	F
0.1670011130004241	0.3015708919986224	0.2584828139999971	F	F	F
0.1651801469798997	0.6348999139821885	0.2584767940000035	F	F	F
0.1633591199893019	0.9682278630196066	0.2584708030000016	F	F	F
0.6624021530220432	0.1420838830036288	0.2569057939999979	F	F	F
0.6605801580010393	0.4754129049871949	0.2568998039999997	F	F	F
0.6587591169823312	0.8087408539792165	0.2568938140000014	F	F	F
0.3290821310018757	0.1402629020016306	0.2547428009999990	F	F	F
0.3272611199915190	0.4735908810020035	0.2547368110000008	F	F	F
0.3254401389895136	0.8069198730226148	0.2547307910000001	F	F	F
0.8734241129859370	0.0502678940068506	0.2223587929999979	F	F	F
0.8716031309851715	0.3835968970138808	0.2223527880000020	F	F	F
0.8697810769916288	0.7169249060226051	0.2223467979999967	F	F	F
0.5401051640027106	0.0484468750063769	0.2201958149999967	F	F	F
0.5382831100091678	0.3817759160118754	0.2201897950000031	F	F	F
0.5364621280084023	0.7151039240218395	0.2201837900000001	F	F	F
0.2067850979914780	0.0466258970006734	0.2180327920000025	F	F	F

0.2049641310175829	0.3799538610195654	0.2180267869999994	F	F	F
0.2031431200072333	0.7132828830031315	0.2180207970000012	F	F	F
0.7250071169868377	0.2004988790164575	0.2167577889999990	F	F	F
0.7231860760135334	0.5338268879797852	0.2167517990000007	F	F	F
0.7213641410092322	0.8671548959897493	0.2167457939999977	F	F	F
0.3916881680036113	0.1986769140038120	0.2145947960000001	F	F	F
0.3898661140100685	0.5320059059790196	0.2145887909999971	F	F	F
0.3880451620176544	0.8653339149877510	0.2145828009999988	F	F	F
0.0583681089837356	0.1968558880119744	0.2124317880000035	F	F	F
0.0547261130217152	0.8635128740144395	0.2124197930000022	F	F	F
0.0565471309780250	0.5301839110034194	0.2124257979999982	F	F	F
0.8336401580032344	0.3052138690203918	0.2628088000000020	F	F	F
0.8318190570132202	0.6385419370016621	0.2628027800000012	F	F	F
0.8299971220089262	0.9718708990139220	0.2627967889999994	F	F	F
0.3757835200031348	0.4591733811593495	0.7040920940518788	T	T	T
0.4813173887426725	0.5259852052267978	0.7480655894592383	T	T	T
0.3527704999045735	0.5380859290012810	0.7157893378423515	T	T	T
0.4175121319542458	0.5793129764851445	0.7425261434611775	T	T	T
0.4549463829867048	0.4521965557895659	0.7253218132219283	T	T	T
0.4253935026150972	0.5182074282797056	0.7021815647532905	T	T	T

:::::::::::::

CH_4 (VASP)

:::::::::::::

E(PBE, VASP) = -24.028625 (eV)

Edisp /kcal,au,eV: -0.7432 -0.00118436 -0.0322280

header

1.00000000000000

22.0272006988999998	0.0000000000000000	0.0000000000000000
0.0000000000000000	22.0272006988999998	0.0000000000000000
0.0000000000000000	0.0000000000000000	25.0000000000000000

C H

1 4

Direct

0.8978081961584471	0.0000000208569366	0.8578485374272109
0.9220021988677616	0.9566058873199326	0.8609134532301347

0.9304929996964398 0.0374885349853317 0.8601632774602556
0.8651223350212589 0.0038246169595985 0.8907917633244431
0.8736130011937860 0.0020809399235901 0.8195387603579509

:::::::::::::
IV (VASP)
:::::::::::::
E(PBE, VASP) = -2613.56429492 (eV)
Edisp /kcal,au,eV: -611.2915 -0.97415486 -26.5081036
header
1.00000000000000000
22.0272006988999998 0.0000000000000000 0.0000000000000000
0.0000000000000000 22.0272006988999998 0.0000000000000000
0.0000000000000000 0.0000000000000000 25.0000000000000000
Si O H W C
90 207 66 1 4
Selective dynamics
Direct
0.8924731015189581 0.1019525835339682 0.5761420856105097 T T T
0.8905924953919622 0.4346235606221068 0.5759143180313653 T T T
0.8896454569620262 0.7684314204741585 0.5763763859887056 T T T
0.5573147196054254 0.1005053983142972 0.5768301230094516 T T T
0.5560334609690223 0.4340831104656801 0.5740946086300779 T T T
0.5549558685837369 0.7684077427061189 0.5770077576099873 T T T
0.2250658028793401 0.0984786510692408 0.5747449663379821 T T T
0.2235278897902177 0.4322806234478132 0.5744607763760751 T T T
0.2222192472743913 0.7650918615665810 0.5750417108258151 T T T
0.7889414084451279 0.2017697360135151 0.5728067389036401 T T T
0.7880157577970673 0.5354083848615107 0.5728043039088877 T T T
0.7868993417758057 0.8690557164916191 0.5731812886084118 T T T
0.4546434512452461 0.2015174816209375 0.5715875252799723 T T T
0.4544730998775880 0.5343463495278841 0.5703321808736109 T T T
0.4519664462449460 0.8689860220443351 0.5719367697393645 T T T
0.1231001576317144 0.1998969512331276 0.5699617090160031 T T T
0.1212212542184778 0.5330601825941815 0.5697442312601055 T T T
0.1201506213238278 0.8668497108330785 0.5705314008759541 T T T

0.8376511786965241	0.3230494889119122	0.5147675955727148	T	T	T
0.8364633434259274	0.6568570802704957	0.5147799450720681	T	T	T
0.8373055418135248	0.9905970186969572	0.5166477825895873	T	T	T
0.0040101558026722	0.1524346559059608	0.5086101460350668	T	T	T
0.0020699929100075	0.4855600077246930	0.5087506887119624	T	T	T
0.0009654517655555	0.8192044286328338	0.5088477056128811	T	T	T
0.5046906567439371	0.3216957426089143	0.5119670087293553	T	T	T
0.5033332111749446	0.6560684546989920	0.5130674715251303	T	T	T
0.5041447066011803	0.9892729742520388	0.5141142675224100	T	T	T
0.6694563837928390	0.1548631586101379	0.5119990675612679	T	T	T
0.6686241090874335	0.4887274830310466	0.5117678506323080	T	T	T
0.6670038128880632	0.8221177919947240	0.5121274150214843	T	T	T
0.1717844425801927	0.3205760751573405	0.5101474189438773	T	T	T
0.1700146880478011	0.6538062687742293	0.5101871320298329	T	T	T
0.1708605131684607	0.9874600716260230	0.5119443944303879	T	T	T
0.3363086398515123	0.1532529425282788	0.5097716682804310	T	T	T
0.3350641320257217	0.4869315886222795	0.5096167434381702	T	T	T
0.3334086957043388	0.8202336981361203	0.5100460285591699	T	T	T
0.9252931479857907	0.2479518950073754	0.4412057999999988	F	F	F
0.9234721060137190	0.5812809469992928	0.4411998089999969	F	F	F
0.9216511250117136	0.9146088959913143	0.4411938189999987	F	F	F
0.7529111499777699	0.0692349000150614	0.4404208059999988	F	F	F
0.7510890959842271	0.4025638999803931	0.4404148160000005	F	F	F
0.7492681149822289	0.7358919379987086	0.4404087959999998	F	F	F
0.5919740800133155	0.2461308839970187	0.4390428069999999	F	F	F
0.5901531579838561	0.5794588920069828	0.4390367869999992	F	F	F
0.5883311029915461	0.9127878550180029	0.4390307959999973	F	F	F
0.4195911590101247	0.0674138810145806	0.4382577840000010	F	F	F
0.4177701179914166	0.4007419050047858	0.4382517929999992	F	F	F
0.4159491659989953	0.7340708370086944	0.4382458030000009	F	F	F
0.2586551010217377	0.2443099019962531	0.4368798140000010	F	F	F
0.2568331360090852	0.5776379110049774	0.4368738229999991	F	F	F
0.2550121249987285	0.9109659190149415	0.4368678029999984	F	F	F
0.0862721130104802	0.0655929070039392	0.4360948200000010	F	F	F
0.0844511320084749	0.3989208939944362	0.4360888000000003	F	F	F
0.0826301130080012	0.7322489020043932	0.4360828100000020	F	F	F

0.8370211119890811	0.1602299059805716	0.3748038109999996	F	F	F
0.8352000710157768	0.4935579000078292	0.3747977909999989	F	F	F
0.8333781360114756	0.8268868919830368	0.3747918010000006	F	F	F
0.5037021039879122	0.1584088799887411	0.3726407890000019	F	F	F
0.5018801090123119	0.4917368889974725	0.3726347980000000	F	F	F
0.5000591280103066	0.8250648379894940	0.3726287779999993	F	F	F
0.1703821270029735	0.1565868999946147	0.3704777960000030	F	F	F
0.1685611460009753	0.4899158779871158	0.3704718050000011	F	F	F
0.1667401190103774	0.8232439160054312	0.3704658150000029	F	F	F
0.6731271740190010	0.3229198749868942	0.3590378170000008	F	F	F
0.6713061330003001	0.6562489269788117	0.3590317959999965	F	F	F
0.6694851519982947	0.9895768760162298	0.3590258059999982	F	F	F
0.3398081360094807	0.3210988939848889	0.3568748240000019	F	F	F
0.3379871249991311	0.6544268729852689	0.3568687739999987	F	F	F
0.3361651000151724	0.9877559539867704	0.3568627829999969	F	F	F
0.0064891129814413	0.3192778830199359	0.3547118010000005	F	F	F
0.0046671389799045	0.6526058909844963	0.3547058110000023	F	F	F
0.0028461179818962	0.9859338999932277	0.3546997899999980	F	F	F
0.9212151169991074	0.0815368740018627	0.2959358100000031	F	F	F
0.9193941349983419	0.4148648979920679	0.2959297900000024	F	F	F
0.9175731539963365	0.7481939200210306	0.2959237990000005	F	F	F
0.5878961680158810	0.0797158999912213	0.2937727870000018	F	F	F
0.5860751269971729	0.4130438570187636	0.2937667970000035	F	F	F
0.5842531320215656	0.7463728790023225	0.2937608060000017	F	F	F
0.7602561120186380	0.2375698979880525	0.2928088009999996	F	F	F
0.7584350709999299	0.5708978769884254	0.2928027809999989	F	F	F
0.7566142079884131	0.9042268990173881	0.2927967910000007	F	F	F
0.2545771300063606	0.0778948739993837	0.2916097940000029	F	F	F
0.2527551349853567	0.4112229049809457	0.2916038039999975	F	F	F
0.2509341240203966	0.7445508839813257	0.2915978130000028	F	F	F
0.4269371030187088	0.2357488869776958	0.2906458080000007	F	F	F
0.4251161220167035	0.5690768959864272	0.2906397880000000	F	F	F
0.4232951100075866	0.9024049039963842	0.2906337980000018	F	F	F
0.0936181169903705	0.2339268619937371	0.2884827849999994	F	F	F
0.0917971359883651	0.5672559139856546	0.2884767950000011	F	F	F
0.0899751189945306	0.9005838629776761	0.2884708050000029	F	F	F

0.9022458885883554	0.1075828355084808	0.6408504944892492	T	T	T
0.9001829193249666	0.4397131176734275	0.6406572218599367	T	T	T
0.8984539739984921	0.7726185998361237	0.6412628291279300	T	T	T
0.5641902549383236	0.1027190033071171	0.6419584423235415	T	T	T
0.5610158031248337	0.4358899469901139	0.6396700002922778	T	T	T
0.5596733241717945	0.7685410635226551	0.6423393647203657	T	T	T
0.2317725486886176	0.1002184752868904	0.6398892263821968	T	T	T
0.2283446808602298	0.4335091534763764	0.6396490104987566	T	T	T
0.2282505883144861	0.7649837979539560	0.6402432319608197	T	T	T
0.7861934511047592	0.2093371964007616	0.6378685686114297	T	T	T
0.7848233248801403	0.5424898412571544	0.6378789977464852	T	T	T
0.7818289740338822	0.8741378436557947	0.6383459005332902	T	T	T
0.4501184379463993	0.2106321715859749	0.6364229515470413	T	T	T
0.4484802435088615	0.5389884338374709	0.6354202719527275	T	T	T
0.4458605744743074	0.8768175157075876	0.6367732639361074	T	T	T
0.1195153455419708	0.2091266774954258	0.6348199508256093	T	T	T
0.1179533131704054	0.5428444186204303	0.6345569720490656	T	T	T
0.1148384075184648	0.8757753475194682	0.6353430313903183	T	T	T
0.8410356803381238	0.1515872242997535	0.5592131751887833	T	T	T
0.8395823865184856	0.4847999127481887	0.5590977574342628	T	T	T
0.8386379672267706	0.8185619840533149	0.5596162553869690	T	T	T
0.5056667735827380	0.1497501600352869	0.5600398585430528	T	T	T
0.5056596566657311	0.4836426303277398	0.5544375626420776	T	T	T
0.5033154962979601	0.8175258519045980	0.5602345273034657	T	T	T
0.1746032957529718	0.1487368913344455	0.5576842572828048	T	T	T
0.1722908409389868	0.4816501634316286	0.5569436529170527	T	T	T
0.1716091137172784	0.8155477381171922	0.5588574318830912	T	T	T
0.8692498347288652	0.0336912909937320	0.5611724336133453	T	T	T
0.8675305480824548	0.3666114521399799	0.5601692347533069	T	T	T
0.8667285720835262	0.7007002264752104	0.5596909564825857	T	T	T
0.5370908082355746	0.0325903481772430	0.5579407467890143	T	T	T
0.5363621407582738	0.3659334851424330	0.5560491918775147	T	T	T
0.5357351097588393	0.7014315484854021	0.5554619219650183	T	T	T
0.2040234856324865	0.0308422518369789	0.5556567074913874	T	T	T
0.2037350137733122	0.3651090041694321	0.5536155763202643	T	T	T
0.2017404759548073	0.6981877816885840	0.5539664729096986	T	T	T

0.9559334080896318	0.1134515241853724	0.5441923460792381	T	T	T
0.9541715653078698	0.4465948894785742	0.5443297233013146	T	T	T
0.9536826080710321	0.7809362854858630	0.5456711019312193	T	T	T
0.6217815863418565	0.1163421948966959	0.5480809671870916	T	T	T
0.6217716067217944	0.4502493887320531	0.5485912389577683	T	T	T
0.6205930623733266	0.7854026018918085	0.5508502330775482	T	T	T
0.7221306774224421	0.1823868154292774	0.5505710337096063	T	T	T
0.7213774239884061	0.5167856645519582	0.5497097599899650	T	T	T
0.7209778789522063	0.8513031928901874	0.5480795569767494	T	T	T
0.2899121706516422	0.1137750124745792	0.5463224316628135	T	T	T
0.2892118646506978	0.4489463101065671	0.5478351280908969	T	T	T
0.2873864951595927	0.7819673297221259	0.5478301849492691	T	T	T
0.8080256471462516	0.2645896187469244	0.5435641818667564	T	T	T
0.8074928221016318	0.5984748112471641	0.5442148123952454	T	T	T
0.8075954889592976	0.9328836836617564	0.5465861351954672	T	T	T
0.3885837149413994	0.1826176041989015	0.5477203613527136	T	T	T
0.3886739050219759	0.5171478363770198	0.5453613284181799	T	T	T
0.3864458169999777	0.8504600350748599	0.5465452738258818	T	T	T
0.4764923524185984	0.2635433827964860	0.5422737445919611	T	T	T
0.4757488320077181	0.5992576192482503	0.5463888296573564	T	T	T
0.4747471034160869	0.9314631498623859	0.5441526897745768	T	T	T
0.0569516463898768	0.1792218586151952	0.5473544522797281	T	T	T
0.0548791698018410	0.5125953810647361	0.5474625611420939	T	T	T
0.0545369940816669	0.8468492783505156	0.5463505334289920	T	T	T
0.1434154179915755	0.2621543981654921	0.5401956034319958	T	T	T
0.1416228962478822	0.5952719377730836	0.5399799681902974	T	T	T
0.1417759502127829	0.9292695341734476	0.5417693059036424	T	T	T
0.7837132636986051	0.0274391659206022	0.4863327756386377	T	T	T
0.7834762502127958	0.3594077448441584	0.4844481061807255	T	T	T
0.7820288733893449	0.6926956140806072	0.4843601489527856	T	T	T
0.9669170521810744	0.2088590191521963	0.4823221112889921	T	T	T
0.9651736590687896	0.5417870716839217	0.4820214783336365	T	T	T
0.9636162533478574	0.8746961811038853	0.4815684542470556	T	T	T
0.4499911740086698	0.0260108314142438	0.4845515973222620	T	T	T
0.4497041547870873	0.3577708817815076	0.4827165275127676	T	T	T
0.4480365959701854	0.6906158655294222	0.4826089370814435	T	T	T

0.6323897037249444	0.2093404370671143	0.4826343158764683	T	T	T
0.6312123078075909	0.5423925707574031	0.4819822978113748	T	T	T
0.6292407867213701	0.8749821752916705	0.4815839069957859	T	T	T
0.1165410050785554	0.0243992237754226	0.4827331183639063	T	T	T
0.1163955937352591	0.3562432797147691	0.4808165674404057	T	T	T
0.1148566629008550	0.6895360713583253	0.4806796198827928	T	T	T
0.2985846803187196	0.2071405331390514	0.4804252846904349	T	T	T
0.2971167950514175	0.5401409824287825	0.4798615077756088	T	T	T
0.2955632870020007	0.8730655133772799	0.4795578409432670	T	T	T
0.8903758845129218	0.3024131868470236	0.4726896195529399	T	T	T
0.8889573107861652	0.6359320530786921	0.4726236924331775	T	T	T
0.8883078155099953	0.9690373843516579	0.4733896697992635	T	T	T
0.5561688614345650	0.3013853219702237	0.4688090880903161	T	T	T
0.5541551006177960	0.6337597517519175	0.4699201325820689	T	T	T
0.5542109043937395	0.9679186731538029	0.4699642348698649	T	T	T
0.7002600005490787	0.1113209449684894	0.4673359667251478	T	T	T
0.6986869979278676	0.4439817036761729	0.4676560127258434	T	T	T
0.6964296896948448	0.7762627382499604	0.4683777906205224	T	T	T
0.2229569798867356	0.2996456062656906	0.4667122025167948	T	T	T
0.2214615354239426	0.6329327922774254	0.4669892697698929	T	T	T
0.2208518202297471	0.9662421975378400	0.4676150104923110	T	T	T
0.3673159490638722	0.1100062974455443	0.4650233699353507	T	T	T
0.3651125724438695	0.4419083972596964	0.4655944852890004	T	T	T
0.3637590876666419	0.7752109541608908	0.4661341701480455	T	T	T
0.0342750478526318	0.1092774457453912	0.4631010806574875	T	T	T
0.0325588004296563	0.4417629766477768	0.4636892932444698	T	T	T
0.0307009951605827	0.7746656969247090	0.4639644565743405	T	T	T
0.8040411469935194	0.1112479120019287	0.4128897789999968	F	F	F
0.8022201659915140	0.4445758760208278	0.4128837880000020	F	F	F
0.8003991839907485	0.7779049280127381	0.4128777979999967	F	F	F
0.8755741120097511	0.2052448690053339	0.4126187860000030	F	F	F
0.8737530709910430	0.5385729069782528	0.4126137789999973	F	F	F
0.8719320889902775	0.8719009159869842	0.4126077889999991	F	F	F
0.4707220790210442	0.1094268860100911	0.4107268149999967	F	F	F
0.4689011279819937	0.4427549239830100	0.4107207950000031	F	F	F
0.4670801160182734	0.7760828730204352	0.4107148049999978	F	F	F

0.5422551040085821	0.2034229039926885	0.4104558229999995	F	F	F
0.5404341220078166	0.5367518660049484	0.4104507859999984	F	F	F
0.5386121269868127	0.8700799349849788	0.4104447960000002	F	F	F
0.1374031309911814	0.1076049130073216	0.4085637929999990	F	F	F
0.1355821489904159	0.4409338830096985	0.4085578019999971	F	F	F
0.1337601240064572	0.7742619509909687	0.4085517820000035	F	F	F
0.2089361399984782	0.2016018780008579	0.4082927999999981	F	F	F
0.2071140999876491	0.5349298719827118	0.4082878230000020	F	F	F
0.2052931489940022	0.8682588930129072	0.4082817729999988	F	F	F
0.9701991080086358	0.2756438849854916	0.3967227939999987	F	F	F
0.9683781859791765	0.6089718939942230	0.3967168029999968	F	F	F
0.9665570859879296	0.9422998429862446	0.3967108129999986	F	F	F
0.7212521429830758	0.0278279130144128	0.3955617849999982	F	F	F
0.7194311020097715	0.3611558970086151	0.3955557940000034	F	F	F
0.7176091670054703	0.6944839359802941	0.3955498039999981	F	F	F
0.6368801000074669	0.2738219200182428	0.3945597709999973	F	F	F
0.6350591180067013	0.6071508530209115	0.3945537809999990	F	F	F
0.6332371229856975	0.9404789210021818	0.3945478199999997	F	F	F
0.3879331649902653	0.0260058919801196	0.3933988209999981	F	F	F
0.3861111099979553	0.3593349160066097	0.3933928009999974	F	F	F
0.3842900989875986	0.6926628950069897	0.3933868109999992	F	F	F
0.3035611509788367	0.2720008789995347	0.3923968080000009	F	F	F
0.3017391259948852	0.6053289180166175	0.3923907880000002	F	F	F
0.2999181449928798	0.9386578799834737	0.3923847969999983	F	F	F
0.0546131130071430	0.0241849160173402	0.3912357990000004	F	F	F
0.0527921389964945	0.3575128910226582	0.3912297789999997	F	F	F
0.0509711169997260	0.6908419130062171	0.3912237879999978	F	F	F
0.7851661440059274	0.1981308910132142	0.3429927829999997	F	F	F
0.7833451630039221	0.5314588549867167	0.3429867919999978	F	F	F
0.7815241219852211	0.8647879359882182	0.3429808019999996	F	F	F
0.4518471059964071	0.1963098939855712	0.3408298189999996	F	F	F
0.4500260949860504	0.5296378729859512	0.3408237700000001	F	F	F
0.4482041000104502	0.8629658819946755	0.3408178090000007	F	F	F
0.1185281349949463	0.1944879000087383	0.3386667970000019	F	F	F
0.1167061180011117	0.5278168919839459	0.3386608060000000	F	F	F
0.1148851360003462	0.8611448999939100	0.3386547859999993	F	F	F

0.8824861050079207	0.1266008759859929	0.3339448270000034	F	F	F
0.8806651230071552	0.4599289000215947	0.3339387770000002	F	F	F
0.8788440819884471	0.7932578329788669	0.3339327870000020	F	F	F
0.9598761199854096	0.0364938789993445	0.3337728079999991	F	F	F
0.9580550790120981	0.3698219059858445	0.3337678019999970	F	F	F
0.9562330839910942	0.7031508679981044	0.3337617809999998	F	F	F
0.5491670970067517	0.1247799100108651	0.3317818049999985	F	F	F
0.5473461750226889	0.4581078590028866	0.3317758139999967	F	F	F
0.5455251340039808	0.7914358970212021	0.3317697940000031	F	F	F
0.6265561579819448	0.0346729049887031	0.3316098150000002	F	F	F
0.6247351170086333	0.3680008650125401	0.3316038250000020	F	F	F
0.6229141350078677	0.7013288740212644	0.3315988180000033	F	F	F
0.2158481329966477	0.1229578849815098	0.3296188119999997	F	F	F
0.2140271070048172	0.4562869070104725	0.3296128209999978	F	F	F
0.2122051419921647	0.7896149160192039	0.3296067710000017	F	F	F
0.2932371499807758	0.0328508839998065	0.3294467930000025	F	F	F
0.2914161090074643	0.3661799130201260	0.3294408020000006	F	F	F
0.2895951570150501	0.6995078920204989	0.3294357959999985	F	F	F
0.7125151159940089	0.2879979009914209	0.3141148090000030	F	F	F
0.7106941339932362	0.6213259100001522	0.3141087890000023	F	F	F
0.7088730930199318	0.9546548720124051	0.3141027989999969	F	F	F
0.3791961069940726	0.2861769199894226	0.3119518159999970	F	F	F
0.3773751259920672	0.6195048689814442	0.3119457960000034	F	F	F
0.3755531310164670	0.9528329370081110	0.3119398059999980	F	F	F
0.0458771439827359	0.2843548950054640	0.3097878100000031	F	F	F
0.0440551229938393	0.6176839469973814	0.3097828029999974	F	F	F
0.0422341449881287	0.9510118959894029	0.3097768129999992	F	F	F
0.8732610939964118	0.0398549059864806	0.2601577940000013	F	F	F
0.8714401720123490	0.3731828630140228	0.2601518029999994	F	F	F
0.8696190710223348	0.7065109009869417	0.2601457829999987	F	F	F
0.5399420859952428	0.0380328859963512	0.2579948010000024	F	F	F
0.5381201509909488	0.3713619110216086	0.2579888109999970	F	F	F
0.5362991090188771	0.7046898600136302	0.2579828200000023	F	F	F
0.8128991129996024	0.2660319209917859	0.2561918199999980	F	F	F
0.8110781309988297	0.5993608830040458	0.2561857999999972	F	F	F
0.8092561359778330	0.9326888920127701	0.2561797799999965	F	F	F

0.2066221390186556	0.0362119119857027	0.2558318080000035	F	F	F
0.2048011119826612	0.3695398869910207	0.2558258179999982	F	F	F
0.2029801460075333	0.7028688790116249	0.2558197979999974	F	F	F
0.4795800749900820	0.2642108800184815	0.2540287969999966	F	F	F
0.4777581389870207	0.5975399020020404	0.2540228069999984	F	F	F
0.4759371280220606	0.9308679100120045	0.2540167869999976	F	F	F
0.7232301240073369	0.1896398960131407	0.2532247899999973	F	F	F
0.7214091420065714	0.5229678750135207	0.2532187999999991	F	F	F
0.7195871469855675	0.8562968369803770	0.2532128100000008	F	F	F
0.9609541890203559	0.1165518979961959	0.2520078120000022	F	F	F
0.9591330889837053	0.4498798549783416	0.2520017920000015	F	F	F
0.9573121669996425	0.7832078929966571	0.2519958020000033	F	F	F
0.1462601129866172	0.2623898980177088	0.2518658039999977	F	F	F
0.1444391460127221	0.5957179069810365	0.2518597839999970	F	F	F
0.1426181350023725	0.9290469290099992	0.2518538240000012	F	F	F
0.3899110849990564	0.1878188850027840	0.2510617969999984	F	F	F
0.3880891499947552	0.5211468940115154	0.2510558070000002	F	F	F
0.3862681090214437	0.8544749020214795	0.2510498170000020	F	F	F
0.6276350619845417	0.1147298810023614	0.2498448039999985	F	F	F
0.6258141400004789	0.4480589029859274	0.2498387839999978	F	F	F
0.6239920850081688	0.7813869119946588	0.2498328089999973	F	F	F
0.0565911229955915	0.1859969050086576	0.2488987890000018	F	F	F
0.0547701489849430	0.5193258519940400	0.2488927989999965	F	F	F
0.0529491269881746	0.8526539210194741	0.2488867940000006	F	F	F
0.2943161430096666	0.1129089000003631	0.2476818109999996	F	F	F
0.2924941479886627	0.4462378920209744	0.2476758059999966	F	F	F
0.2906731369783060	0.7795658710213473	0.2476697860000030	F	F	F
0.9196617429856628	0.7396962197720592	0.6589426380634757	T	T	T
0.5855775091221986	0.0697254915422365	0.6593373327013287	T	T	T
0.2494555737071759	0.7313473599392923	0.6568591799603354	T	T	T
0.7489880483540839	0.2250337376512092	0.6532863850818252	T	T	T
0.0815536312733812	0.2236404771004823	0.6496552849495593	T	T	T
0.0789430698449475	0.5534608822792358	0.6498235648279230	T	T	T
0.9233939676241010	0.0749637706247542	0.6589944180710369	T	T	T
0.9201952589525590	0.4063812415597403	0.6588030914012019	T	T	T
0.5639202942332954	0.3969390383530103	0.6575712007858412	T	T	T

0.5876259873514897	0.7402984999378545	0.6589758998867504	T	T	T
0.2514635992720742	0.0661679513709956	0.6572473617154860	T	T	T
0.2622334276940529	0.4132128775627449	0.6568011634666659	T	T	T
0.7476439972009549	0.5582186725916980	0.6532890432346737	T	T	T
0.7454987964766761	0.8923877134719784	0.6531623520990186	T	T	T
0.4129671752844131	0.2278814270877476	0.6506247302674760	T	T	T
0.4071172544497607	0.8900877487461663	0.6509206357788820	T	T	T
0.0759594303098655	0.8885480517627325	0.6495633033418624	T	T	T
0.5003200770105281	0.3033929169825811	0.2606458070000031	F	F	F
0.4984991549810687	0.6367208959829540	0.2606397870000023	F	F	F
0.4966780839994058	0.9700488450203792	0.2606337960000005	F	F	F
0.9957210419885669	0.1439058930061066	0.2590687869999968	F	F	F
0.9939001800212068	0.4772338869879604	0.2590627969999986	F	F	F
0.9920781249835002	0.8105618359799820	0.2590568070000003	F	F	F
0.1670011130004241	0.3015708919986224	0.2584828139999971	F	F	F
0.1651801469798997	0.6348999139821885	0.2584767940000035	F	F	F
0.1633591199893019	0.9682278630196066	0.2584708030000016	F	F	F
0.6624021530220432	0.1420838830036288	0.2569057939999979	F	F	F
0.6605801580010393	0.4754129049871949	0.2568998039999997	F	F	F
0.6587591169823312	0.8087408539792165	0.2568938140000014	F	F	F
0.3290821310018757	0.1402629020016306	0.2547428009999990	F	F	F
0.3272611199915190	0.4735908810020035	0.2547368110000008	F	F	F
0.3254401389895136	0.8069198730226148	0.2547307910000001	F	F	F
0.8734241129859370	0.0502678940068506	0.2223587929999979	F	F	F
0.8716031309851715	0.3835968970138808	0.2223527880000020	F	F	F
0.8697810769916288	0.7169249060226051	0.2223467979999967	F	F	F
0.5401051640027106	0.0484468750063769	0.2201958149999967	F	F	F
0.5382831100091678	0.3817759160118754	0.2201897950000031	F	F	F
0.5364621280084023	0.7151039240218395	0.2201837900000001	F	F	F
0.2067850979914780	0.0466258970006734	0.2180327920000025	F	F	F
0.2049641310175829	0.3799538610195654	0.2180267869999994	F	F	F
0.2031431200072333	0.7132828830031315	0.2180207970000012	F	F	F
0.7250071169868377	0.2004988790164575	0.2167577889999990	F	F	F
0.7231860760135334	0.5338268879797852	0.2167517990000007	F	F	F
0.7213641410092322	0.8671548959897493	0.2167457939999977	F	F	F
0.3916881680036113	0.1986769140038120	0.2145947960000001	F	F	F

0.3898661140100685	0.5320059059790196	0.2145887909999971	F	F	F
0.3880451620176544	0.8653339149877510	0.2145828009999988	F	F	F
0.0583681089837356	0.1968558880119744	0.2124317880000035	F	F	F
0.0547261130217152	0.8635128740144395	0.2124197930000022	F	F	F
0.0565471309780250	0.5301839110034194	0.2124257979999982	F	F	F
0.8336401580032344	0.3052138690203918	0.2628088000000020	F	F	F
0.8318190570132202	0.6385419370016621	0.2628027800000012	F	F	F
0.8299971220089262	0.9718708990139220	0.2627967889999994	F	F	F
0.3373960491719857	0.5904470650323721	0.7018703506238171	T	T	T
0.3635300994346051	0.5881471593360595	0.7695071748339762	T	T	T
0.4094234050493192	0.6229113243865001	0.7184536304522567	T	T	T
0.3075123348668212	0.4894609446584205	0.7331481183197155	T	T	T
0.3464831196908283	0.4206521850446140	0.7496191146779150	T	T	T
0.3564023573158111	0.4868153828901049	0.7894781236756180	T	T	T
0.3890757353951219	0.3873062453017020	0.6800588567993573	T	T	T
0.3658479323179804	0.4423503618855176	0.6311487644111168	T	T	T
0.4422204963644083	0.4154262036425749	0.6325286751917544	T	T	T
0.5046357799291683	0.5149873376366703	0.7893205121846444	T	T	T
0.5471150295180002	0.4944680346516511	0.7313235226577048	T	T	T
0.5217663209747138	0.5715383255668967	0.7394013573052680	T	T	T
0.4479633168035756	0.4431652623800457	0.7379266404488469	T	T	T
0.4284068568633336	0.5070811035744249	0.7045436537498745	T	T	T
0.3777219615430210	0.5852881705197245	0.7275187356234900	T	T	T
0.3505713898058316	0.4702212523591168	0.7480020879653271	T	T	T
0.4030756883627677	0.4276961477411125	0.6577509878360116	T	T	T
0.5099166162219767	0.5232791087328187	0.7460873783370311	T	T	T

::::::::::::::

I + $\mathbf{H_2}$ complex (VASP)

::::::::::::::

E(PBE, VASP) = -2636.66356631 (eV)

Edisp /kcal,au,eV: -614.9065 -0.97991571 -26.6648644

header

1.00000000000000000

22.0272006988999998	0.0000000000000000	0.0000000000000000
0.0000000000000000	22.0272006988999998	0.0000000000000000

0.0000000000000000 0.0000000000000000 25.0000000000000000

Si O H W C

90 207 70 1 5

Selective dynamics

Direct

0.8913831100037465	0.1008611107184273	0.5758262883422410	T	T	T
0.8897794370568297	0.4336628978320103	0.5759081459410101	T	T	T
0.8884460502116260	0.7675434159383697	0.5759255210105242	T	T	T
0.5564676379087813	0.0996240461389898	0.5765239607250804	T	T	T
0.5556623701934194	0.4326133201364563	0.5742007715792592	T	T	T
0.5539284594772160	0.7663536762218709	0.5767709214166354	T	T	T
0.2239748355149312	0.0973155228041349	0.5745963393793705	T	T	T
0.2229735011365834	0.4303578119403439	0.5745809655961981	T	T	T
0.2212959022563204	0.7640780094857538	0.5749112498982395	T	T	T
0.7879784043261874	0.2012839149251073	0.5727896229975347	T	T	T
0.7868971086160834	0.5346977157882905	0.5728886536169129	T	T	T
0.7856268398903247	0.8683949628632958	0.5730953751001920	T	T	T
0.4538991510464075	0.2006711889288816	0.5712404878189798	T	T	T
0.4526407574205956	0.5335564307553106	0.5722290202736586	T	T	T
0.4516336803769591	0.8679387255506390	0.5716441086471139	T	T	T
0.1217025028551489	0.1991860802575255	0.5697684078787872	T	T	T
0.1206429268511379	0.5323328628999957	0.5698807200845227	T	T	T
0.1191968989090097	0.8663084481212305	0.5703240481565973	T	T	T
0.8371798692755476	0.3223931465257232	0.5146208399375429	T	T	T
0.8357391959627873	0.6560264256858978	0.5146931292036712	T	T	T
0.8367340274012612	0.9897034470069952	0.5164516109876621	T	T	T
0.0030202730647517	0.1517663781946290	0.5085607908645401	T	T	T
0.0014981166620279	0.4851251903741720	0.5087580869752159	T	T	T
0.0001549716465527	0.8186556364789916	0.5088187753767042	T	T	T
0.5041227108963832	0.3209374065850104	0.5118948601149241	T	T	T
0.5023225038997232	0.6544729771630621	0.5127989705568740	T	T	T
0.5036923897535845	0.9885081936766694	0.5139787344426398	T	T	T
0.6687688657411981	0.1543802598271147	0.5119937820264227	T	T	T
0.6679742689549059	0.4882397307313352	0.5113226111375745	T	T	T
0.6659734066959245	0.8214408606166689	0.5121849488046900	T	T	T
0.1710663170974160	0.3194662782544047	0.5098536528834705	T	T	T

0.1694749669992996	0.6529970152452371	0.5100322650616812	T	T	T
0.1703653388571398	0.9866439956899119	0.5116218247620734	T	T	T
0.3355451004741127	0.1527278390241062	0.5097676251294208	T	T	T
0.3343310424048649	0.4863810307868651	0.5099889272335093	T	T	T
0.3327721971500038	0.8197705705842744	0.5100909634810326	T	T	T
0.9252931479857907	0.2479518950073754	0.4412057999999988	F	F	F
0.9234721060137190	0.5812809469992928	0.4411998089999969	F	F	F
0.9216511250117136	0.9146088959913143	0.4411938189999987	F	F	F
0.7529111499777699	0.0692349000150614	0.4404208059999988	F	F	F
0.7510890959842271	0.4025638999803931	0.4404148160000005	F	F	F
0.7492681149822289	0.7358919379987086	0.4404087959999998	F	F	F
0.5919740800133155	0.2461308839970187	0.4390428069999999	F	F	F
0.5901531579838561	0.5794588920069828	0.4390367869999992	F	F	F
0.5883311029915461	0.9127878550180029	0.4390307959999973	F	F	F
0.4195911590101247	0.0674138810145806	0.4382577840000010	F	F	F
0.4177701179914166	0.4007419050047858	0.4382517929999992	F	F	F
0.4159491659989953	0.7340708370086944	0.4382458030000009	F	F	F
0.2586551010217377	0.2443099019962531	0.4368798140000010	F	F	F
0.2568331360090852	0.5776379110049774	0.4368738229999991	F	F	F
0.2550121249987285	0.9109659190149415	0.4368678029999984	F	F	F
0.0862721130104802	0.0655929070039392	0.4360948200000010	F	F	F
0.0844511320084749	0.3989208939944362	0.4360888000000003	F	F	F
0.0826301130080012	0.7322489020043932	0.4360828100000020	F	F	F
0.8370211119890811	0.1602299059805716	0.3748038109999996	F	F	F
0.8352000710157768	0.4935579000078292	0.3747977909999989	F	F	F
0.8333781360114756	0.8268868919830368	0.3747918010000006	F	F	F
0.5037021039879122	0.1584088799887411	0.3726407890000019	F	F	F
0.5018801090123119	0.4917368889974725	0.3726347980000000	F	F	F
0.5000591280103066	0.8250648379894940	0.3726287779999993	F	F	F
0.1703821270029735	0.1565868999946147	0.3704777960000030	F	F	F
0.1685611460009753	0.4899158779871158	0.3704718050000011	F	F	F
0.1667401190103774	0.8232439160054312	0.3704658150000029	F	F	F
0.6731271740190010	0.3229198749868942	0.3590378170000008	F	F	F
0.6713061330003001	0.6562489269788117	0.3590317959999965	F	F	F
0.6694851519982947	0.9895768760162298	0.3590258059999982	F	F	F
0.3398081360094807	0.3210988939848889	0.3568748240000019	F	F	F

0.3379871249991311	0.6544268729852689	0.3568687739999987	F	F	F
0.3361651000151724	0.9877559539867704	0.3568627829999969	F	F	F
0.0064891129814413	0.3192778830199359	0.3547118010000005	F	F	F
0.0046671389799045	0.6526058909844963	0.3547058110000023	F	F	F
0.0028461179818962	0.9859338999932277	0.3546997899999980	F	F	F
0.9212151169991074	0.0815368740018627	0.2959358100000031	F	F	F
0.9193941349983419	0.4148648979920679	0.2959297900000024	F	F	F
0.9175731539963365	0.7481939200210306	0.2959237990000005	F	F	F
0.5878961680158810	0.0797158999912213	0.2937727870000018	F	F	F
0.5860751269971729	0.4130438570187636	0.2937667970000035	F	F	F
0.5842531320215656	0.7463728790023225	0.2937608060000017	F	F	F
0.7602561120186380	0.2375698979880525	0.2928088009999996	F	F	F
0.7584350709999299	0.5708978769884254	0.2928027809999989	F	F	F
0.7566142079884131	0.9042268990173881	0.2927967910000007	F	F	F
0.2545771300063606	0.0778948739993837	0.2916097940000029	F	F	F
0.2527551349853567	0.4112229049809457	0.2916038039999975	F	F	F
0.2509341240203966	0.7445508839813257	0.2915978130000028	F	F	F
0.4269371030187088	0.2357488869776958	0.2906458080000007	F	F	F
0.4251161220167035	0.5690768959864272	0.2906397880000000	F	F	F
0.4232951100075866	0.9024049039963842	0.2906337980000018	F	F	F
0.0936181169903705	0.2339268619937371	0.2884827849999994	F	F	F
0.0917971359883651	0.5672559139856546	0.2884767950000011	F	F	F
0.0899751189945306	0.9005838629776761	0.2884708050000029	F	F	F
0.9032933891085109	0.1069250293571360	0.6402425463182377	T	T	T
0.9006764049468667	0.4387346146025297	0.6405032464976061	T	T	T
0.8986289021693575	0.7722723059656114	0.6406747853880173	T	T	T
0.5641721118665174	0.1024392069936721	0.6415556450476034	T	T	T
0.5657330628391245	0.4355939035407123	0.6391778430609391	T	T	T
0.5619675868123679	0.7658398043730907	0.6418284428416809	T	T	T
0.2322085199279707	0.0987953102409087	0.6396611037291862	T	T	T
0.2327788250873552	0.4295283242371878	0.6395090437894843	T	T	T
0.2285617798658200	0.7636737493738377	0.6400686968245803	T	T	T
0.7854752420674650	0.2110312154572483	0.6376498152029845	T	T	T
0.7845614407957981	0.5431384164373827	0.6378772587299649	T	T	T
0.7814430014485464	0.8765563391528878	0.6380323398344548	T	T	T
0.4503945772070702	0.2111111691571831	0.6359187063222156	T	T	T

0.4462614949154739	0.5414441817905364	0.6365626002735870	T	T	T
0.4461592968138009	0.8772803008986422	0.6363994218628595	T	T	T
0.1180711314878060	0.2135951877945588	0.6339942768285283	T	T	T
0.1167853644423536	0.5441390173258746	0.6344851467476378	T	T	T
0.1140621202023740	0.8793009690840243	0.6346603502772541	T	T	T
0.8397813567208307	0.1505733001764100	0.5596122694297893	T	T	T
0.8378730009421379	0.4832827902139371	0.5600095057943887	T	T	T
0.8371761725053138	0.8174709498947991	0.5595233509071120	T	T	T
0.5046750235233803	0.1486256235663440	0.5595466437846424	T	T	T
0.5028292724538957	0.4810043858474837	0.5585301663085362	T	T	T
0.5027003492190316	0.8161751558885939	0.5598375183908244	T	T	T
0.1729346102297432	0.1474082888443161	0.5583434802431969	T	T	T
0.1720352185209794	0.4808341922668450	0.5584502645812270	T	T	T
0.1704286420185007	0.8145081404950065	0.5590362736127127	T	T	T
0.8678762054070038	0.0323676615446499	0.5618612859990351	T	T	T
0.8669006536375969	0.3654605864542200	0.5605234002578036	T	T	T
0.8654267138092724	0.6993695386943320	0.5603912695962716	T	T	T
0.5361864244811583	0.0313513842735934	0.5584834791319364	T	T	T
0.5349493705272159	0.3645997233593757	0.5567777863605686	T	T	T
0.5335631317672658	0.6990111038037028	0.5567027234137288	T	T	T
0.2029199749972877	0.0293597658812537	0.5562453228467934	T	T	T
0.2016732061797129	0.3629614409127411	0.5550759426223405	T	T	T
0.2006593095332395	0.6968658654514756	0.5545810898186915	T	T	T
0.9539357368818484	0.1122011865914865	0.5424432305509527	T	T	T
0.9526731010110553	0.4462321579602637	0.5434198585023722	T	T	T
0.9519485114876463	0.7797839232111764	0.5442118026155163	T	T	T
0.6205845811776128	0.1153885825893429	0.5470881624069331	T	T	T
0.6198876377607103	0.4482081973427824	0.5453925498872155	T	T	T
0.6185339465311206	0.7838313118751143	0.5490550181434140	T	T	T
0.7208927784271413	0.1814873734502803	0.5514738929862311	T	T	T
0.7199883765772237	0.5149963363451318	0.5508725288966061	T	T	T
0.7190538477638805	0.8497489020764575	0.5498508652919141	T	T	T
0.2879966064379727	0.1130981582435905	0.5450395974285377	T	T	T
0.2870880218502890	0.4475379617348898	0.5460705699533932	T	T	T
0.2859662747318500	0.7813076570522197	0.5469982077912992	T	T	T
0.8073836288879458	0.2633935494334700	0.5423979705696922	T	T	T

0.8059143131277667	0.5972073852169589	0.5429699339978339	T	T	T
0.8066737562782937	0.9311361607774553	0.5447188440766125	T	T	T
0.3872065262556256	0.1812550621563201	0.5488212243349619	T	T	T
0.3866863298295855	0.5151357906106699	0.5480209087776179	T	T	T
0.3854638344592953	0.8492580132705844	0.5474866328886715	T	T	T
0.4752832394632343	0.2619449214677579	0.5404784516833516	T	T	T
0.4745311471613636	0.5963784961115939	0.5440895923013287	T	T	T
0.4742166796989433	0.9300132826541939	0.5429468127565603	T	T	T
0.0551621931545802	0.1772778434434153	0.5489821272081856	T	T	T
0.0542244048767149	0.5110858940428821	0.5482012309561871	T	T	T
0.0531079387384412	0.8452237939971637	0.5477592665683756	T	T	T
0.1424732465992398	0.2599559779212640	0.5380202069328556	T	T	T
0.1409224711914280	0.5938479116679360	0.5388791462035037	T	T	T
0.1411157422989866	0.9276086282487308	0.5400136677046035	T	T	T
0.7836571395243519	0.0272050221973067	0.4861318460112242	T	T	T
0.7831748367531395	0.3594095218089640	0.4846249256921486	T	T	T
0.7818700603860025	0.6924907034980771	0.4842207969052561	T	T	T
0.9670103421319894	0.2090513403428300	0.4824871141962674	T	T	T
0.9654058469123111	0.5419078107758930	0.4820460160959771	T	T	T
0.9637222911484038	0.8748190158272811	0.4816596165329104	T	T	T
0.4498291374578675	0.0257977236044453	0.4844929297894336	T	T	T
0.4494907856981004	0.3574854998907626	0.4825306931152735	T	T	T
0.4476783786102176	0.6902384837186312	0.4824929571129508	T	T	T
0.6323878939233450	0.2092982385588055	0.4826283783726041	T	T	T
0.6313570196688404	0.5429173444885721	0.4822320111067795	T	T	T
0.6293574263632329	0.8751514757139233	0.4816429974297393	T	T	T
0.1163877458308080	0.0242683595946024	0.4826614233588963	T	T	T
0.1162473810703594	0.3560821902838961	0.4807426185446608	T	T	T
0.1145982506621845	0.6893370581680798	0.4806823254940658	T	T	T
0.2985450333995950	0.2072363818039842	0.4804936795234854	T	T	T
0.2972969167118262	0.5402857203745840	0.4799727880174988	T	T	T
0.2955174719895775	0.8731331818874324	0.4796651096942098	T	T	T
0.8902190012148681	0.3024272829458441	0.4725645398818821	T	T	T
0.8888689244949186	0.6356320229564620	0.4729564422983784	T	T	T
0.8884381283585022	0.9690816387003244	0.4734373938664982	T	T	T
0.5562097302817869	0.3013631682102976	0.4689060320586265	T	T	T

0.5540326548836930	0.6338019034839423	0.4698058339460890	T	T	T
0.5541168636877019	0.9679428164016484	0.4698359644278048	T	T	T
0.7003981556513641	0.1113618941701160	0.4673912656616772	T	T	T
0.6988976151331626	0.4446256393651035	0.4669128747385860	T	T	T
0.6966297064268177	0.7764898019469526	0.4683457990814818	T	T	T
0.2230502015405170	0.2998279389050558	0.4667259459299780	T	T	T
0.2213226824708924	0.6328869359566811	0.4669623423817054	T	T	T
0.2208606919645595	0.9663989068227182	0.4674099714895488	T	T	T
0.3672292011334759	0.1099913923159500	0.4649951537128583	T	T	T
0.3654116080294322	0.4422391558018466	0.4657249997888266	T	T	T
0.3636973261671411	0.7752268672906140	0.4661053193407466	T	T	T
0.0343975544988710	0.1095515120109465	0.4629938374911018	T	T	T
0.0324249138261595	0.4416373075668057	0.4636814876659208	T	T	T
0.0307227403396231	0.7747925725685553	0.4638758628008176	T	T	T
0.8040411469935194	0.1112479120019287	0.4128897789999968	F	F	F
0.8022201659915140	0.4445758760208278	0.4128837880000020	F	F	F
0.8003991839907485	0.7779049280127381	0.4128777979999967	F	F	F
0.8755741120097511	0.2052448690053339	0.4126187860000030	F	F	F
0.8737530709910430	0.5385729069782528	0.4126137789999973	F	F	F
0.8719320889902775	0.8719009159869842	0.4126077889999991	F	F	F
0.4707220790210442	0.1094268860100911	0.4107268149999967	F	F	F
0.4689011279819937	0.4427549239830100	0.4107207950000031	F	F	F
0.4670801160182734	0.7760828730204352	0.4107148049999978	F	F	F
0.5422551040085821	0.2034229039926885	0.4104558229999995	F	F	F
0.5404341220078166	0.5367518660049484	0.4104507859999984	F	F	F
0.5386121269868127	0.8700799349849788	0.4104447960000002	F	F	F
0.1374031309911814	0.1076049130073216	0.4085637929999990	F	F	F
0.1355821489904159	0.4409338830096985	0.4085578019999971	F	F	F
0.1337601240064572	0.7742619509909687	0.4085517820000035	F	F	F
0.2089361399984782	0.2016018780008579	0.4082927999999981	F	F	F
0.2071140999876491	0.5349298719827118	0.4082878230000020	F	F	F
0.2052931489940022	0.8682588930129072	0.4082817729999988	F	F	F
0.9701991080086358	0.2756438849854916	0.3967227939999987	F	F	F
0.9683781859791765	0.6089718939942230	0.3967168029999968	F	F	F
0.9665570859879296	0.9422998429862446	0.3967108129999986	F	F	F
0.7212521429830758	0.0278279130144128	0.3955617849999982	F	F	F

0.7194311020097715	0.3611558970086151	0.3955557940000034	F	F	F
0.7176091670054703	0.6944839359802941	0.3955498039999981	F	F	F
0.6368801000074669	0.2738219200182428	0.3945597709999973	F	F	F
0.6350591180067013	0.6071508530209115	0.3945537809999990	F	F	F
0.6332371229856975	0.9404789210021818	0.3945478199999997	F	F	F
0.3879331649902653	0.0260058919801196	0.3933988209999981	F	F	F
0.3861111099979553	0.3593349160066097	0.3933928009999974	F	F	F
0.3842900989875986	0.6926628950069897	0.3933868109999992	F	F	F
0.3035611509788367	0.2720008789995347	0.3923968080000009	F	F	F
0.3017391259948852	0.6053289180166175	0.3923907880000002	F	F	F
0.2999181449928798	0.9386578799834737	0.3923847969999983	F	F	F
0.0546131130071430	0.0241849160173402	0.3912357990000004	F	F	F
0.0527921389964945	0.3575128910226582	0.3912297789999997	F	F	F
0.0509711169997260	0.6908419130062171	0.3912237879999978	F	F	F
0.7851661440059274	0.1981308910132142	0.3429927829999997	F	F	F
0.7833451630039221	0.5314588549867167	0.3429867919999978	F	F	F
0.7815241219852211	0.8647879359882182	0.3429808019999996	F	F	F
0.4518471059964071	0.1963098939855712	0.3408298189999996	F	F	F
0.4500260949860504	0.5296378729859512	0.3408237700000001	F	F	F
0.4482041000104502	0.8629658819946755	0.3408178090000007	F	F	F
0.1185281349949463	0.1944879000087383	0.3386667970000019	F	F	F
0.1167061180011117	0.5278168919839459	0.3386608060000000	F	F	F
0.1148851360003462	0.8611448999939100	0.3386547859999993	F	F	F
0.8824861050079207	0.1266008759859929	0.3339448270000034	F	F	F
0.8806651230071552	0.4599289000215947	0.3339387770000002	F	F	F
0.8788440819884471	0.7932578329788669	0.3339327870000020	F	F	F
0.9598761199854096	0.0364938789993445	0.3337728079999991	F	F	F
0.9580550790120981	0.3698219059858445	0.3337678019999970	F	F	F
0.9562330839910942	0.7031508679981044	0.3337617809999998	F	F	F
0.5491670970067517	0.1247799100108651	0.3317818049999985	F	F	F
0.5473461750226889	0.4581078590028866	0.3317758139999967	F	F	F
0.5455251340039808	0.7914358970212021	0.3317697940000031	F	F	F
0.6265561579819448	0.0346729049887031	0.3316098150000002	F	F	F
0.6247351170086333	0.3680008650125401	0.3316038250000020	F	F	F
0.6229141350078677	0.7013288740212644	0.3315988180000033	F	F	F
0.2158481329966477	0.1229578849815098	0.3296188119999997	F	F	F

0.2140271070048172	0.4562869070104725	0.3296128209999978	F	F	F
0.2122051419921647	0.7896149160192039	0.3296067710000017	F	F	F
0.2932371499807758	0.0328508839998065	0.3294467930000025	F	F	F
0.2914161090074643	0.3661799130201260	0.3294408020000006	F	F	F
0.2895951570150501	0.6995078920204989	0.3294357959999985	F	F	F
0.7125151159940089	0.2879979009914209	0.3141148090000030	F	F	F
0.7106941339932362	0.6213259100001522	0.3141087890000023	F	F	F
0.7088730930199318	0.9546548720124051	0.3141027989999969	F	F	F
0.3791961069940726	0.2861769199894226	0.3119518159999970	F	F	F
0.3773751259920672	0.6195048689814442	0.3119457960000034	F	F	F
0.3755531310164670	0.9528329370081110	0.3119398059999980	F	F	F
0.0458771439827359	0.2843548950054640	0.3097878100000031	F	F	F
0.0440551229938393	0.6176839469973814	0.3097828029999974	F	F	F
0.0422341449881287	0.9510118959894029	0.3097768129999992	F	F	F
0.8732610939964118	0.0398549059864806	0.2601577940000013	F	F	F
0.8714401720123490	0.3731828630140228	0.2601518029999994	F	F	F
0.8696190710223348	0.7065109009869417	0.2601457829999987	F	F	F
0.5399420859952428	0.0380328859963512	0.2579948010000024	F	F	F
0.5381201509909488	0.3713619110216086	0.2579888109999970	F	F	F
0.5362991090188771	0.7046898600136302	0.2579828200000023	F	F	F
0.8128991129996024	0.2660319209917859	0.2561918199999980	F	F	F
0.8110781309988297	0.5993608830040458	0.2561857999999972	F	F	F
0.8092561359778330	0.9326888920127701	0.2561797799999965	F	F	F
0.2066221390186556	0.0362119119857027	0.2558318080000035	F	F	F
0.2048011119826612	0.3695398869910207	0.2558258179999982	F	F	F
0.2029801460075333	0.7028688790116249	0.2558197979999974	F	F	F
0.4795800749900820	0.2642108800184815	0.2540287969999966	F	F	F
0.4777581389870207	0.5975399020020404	0.2540228069999984	F	F	F
0.4759371280220606	0.9308679100120045	0.2540167869999976	F	F	F
0.7232301240073369	0.1896398960131407	0.2532247899999973	F	F	F
0.7214091420065714	0.5229678750135207	0.2532187999999991	F	F	F
0.7195871469855675	0.8562968369803770	0.2532128100000008	F	F	F
0.9609541890203559	0.1165518979961959	0.2520078120000022	F	F	F
0.9591330889837053	0.4498798549783416	0.2520017920000015	F	F	F
0.9573121669996425	0.7832078929966571	0.2519958020000033	F	F	F
0.1462601129866172	0.2623898980177088	0.2518658039999977	F	F	F

0.1444391460127221	0.5957179069810365	0.2518597839999970	F	F	F
0.1426181350023725	0.9290469290099992	0.2518538240000012	F	F	F
0.3899110849990564	0.1878188850027840	0.2510617969999984	F	F	F
0.3880891499947552	0.5211468940115154	0.2510558070000002	F	F	F
0.3862681090214437	0.8544749020214795	0.2510498170000020	F	F	F
0.6276350619845417	0.1147298810023614	0.2498448039999985	F	F	F
0.6258141400004789	0.4480589029859274	0.2498387839999978	F	F	F
0.6239920850081688	0.7813869119946588	0.2498328089999973	F	F	F
0.0565911229955915	0.1859969050086576	0.2488987890000018	F	F	F
0.0547701489849430	0.5193258519940400	0.2488927989999965	F	F	F
0.0529491269881746	0.8526539210194741	0.2488867940000006	F	F	F
0.2943161430096666	0.1129089000003631	0.2476818109999996	F	F	F
0.2924941479886627	0.4462378920209744	0.2476758059999966	F	F	F
0.2906731369783060	0.7795658710213473	0.2476697860000030	F	F	F
0.9198197950228655	0.7391656689795345	0.6581069701996806	T	T	T
0.5827304640294138	0.0679506883123191	0.6592142637773493	T	T	T
0.2417081704923104	0.7261375629783160	0.6566831455963117	T	T	T
0.7463179751477188	0.2203558166636539	0.6532976095868936	T	T	T
0.0783571697562393	0.2111585978181071	0.6505303525910601	T	T	T
0.0769825271993870	0.5522895428822083	0.6493567177433702	T	T	T
0.9231627906006780	0.0732619056251934	0.6580486464397771	T	T	T
0.9212061275086878	0.4052776065379678	0.6580313722880715	T	T	T
0.6023914229162622	0.4168962354181462	0.6530480474662713	T	T	T
0.5641224637646036	0.7265090986900157	0.6592363534836158	T	T	T
0.2460693280040335	0.0619164800898861	0.6569191319206824	T	T	T
0.2298827567519655	0.3904250633935392	0.6570899729138304	T	T	T
0.7557116247146982	0.5719886071125420	0.6524192676563975	T	T	T
0.7414234054776025	0.8815517179067354	0.6535143916010536	T	T	T
0.4122879336166313	0.2252330244900930	0.6507671088315792	T	T	T
0.4061106006623838	0.8847194791487593	0.6510284688125311	T	T	T
0.0739825314583349	0.8770907720246875	0.6505071411540662	T	T	T
0.5003200770105281	0.3033929169825811	0.2606458070000031	F	F	F
0.4984991549810687	0.6367208959829540	0.2606397870000023	F	F	F
0.4966780839994058	0.9700488450203792	0.2606337960000005	F	F	F
0.9957210419885669	0.1439058930061066	0.2590687869999968	F	F	F
0.9939001800212068	0.4772338869879604	0.2590627969999986	F	F	F

0.9920781249835002	0.8105618359799820	0.2590568070000003	F	F	F
0.1670011130004241	0.3015708919986224	0.2584828139999971	F	F	F
0.1651801469798997	0.6348999139821885	0.2584767940000035	F	F	F
0.1633591199893019	0.9682278630196066	0.2584708030000016	F	F	F
0.6624021530220432	0.1420838830036288	0.2569057939999979	F	F	F
0.6605801580010393	0.4754129049871949	0.2568998039999997	F	F	F
0.6587591169823312	0.8087408539792165	0.2568938140000014	F	F	F
0.3290821310018757	0.1402629020016306	0.2547428009999990	F	F	F
0.3272611199915190	0.4735908810020035	0.2547368110000008	F	F	F
0.3254401389895136	0.8069198730226148	0.2547307910000001	F	F	F
0.8734241129859370	0.0502678940068506	0.2223587929999979	F	F	F
0.8716031309851715	0.3835968970138808	0.2223527880000020	F	F	F
0.8697810769916288	0.7169249060226051	0.2223467979999967	F	F	F
0.5401051640027106	0.0484468750063769	0.2201958149999967	F	F	F
0.5382831100091678	0.3817759160118754	0.2201897950000031	F	F	F
0.5364621280084023	0.7151039240218395	0.2201837900000001	F	F	F
0.2067850979914780	0.0466258970006734	0.2180327920000025	F	F	F
0.2049641310175829	0.3799538610195654	0.2180267869999994	F	F	F
0.2031431200072333	0.7132828830031315	0.2180207970000012	F	F	F
0.7250071169868377	0.2004988790164575	0.2167577889999990	F	F	F
0.7231860760135334	0.5338268879797852	0.2167517990000007	F	F	F
0.7213641410092322	0.8671548959897493	0.2167457939999977	F	F	F
0.3916881680036113	0.1986769140038120	0.2145947960000001	F	F	F
0.3898661140100685	0.5320059059790196	0.2145887909999971	F	F	F
0.3880451620176544	0.8653339149877510	0.2145828009999988	F	F	F
0.0583681089837356	0.1968558880119744	0.2124317880000035	F	F	F
0.0547261130217152	0.8635128740144395	0.2124197930000022	F	F	F
0.0565471309780250	0.5301839110034194	0.2124257979999982	F	F	F
0.8336401580032344	0.3052138690203918	0.2628088000000020	F	F	F
0.8318190570132202	0.6385419370016621	0.2628027800000012	F	F	F
0.8299971220089262	0.9718708990139220	0.2627967889999994	F	F	F
0.5583886497644229	0.5351858640095530	0.7852957777363735	T	T	T
0.5172266518546619	0.4655975573492377	0.7911024297275070	T	T	T
0.5597851758812933	0.4810738629812701	0.7322445937214314	T	T	T
0.5644859243325941	0.5626603190527896	0.6649909516462109	T	T	T
0.5203668570283454	0.6307082155829927	0.6687819962647308	T	T	T

0.5666192407994618	0.6077947441878900	0.7247870269866512	T	T	T
0.3462691449929301	0.5870070602199352	0.7583475919020578	T	T	T
0.3648372741987642	0.6194406579663234	0.6947139119871530	T	T	T
0.3369647902517464	0.5436982903829483	0.6980963938515369	T	T	T
0.4594798206187451	0.6565365322339225	0.7483434031075193	T	T	T
0.4279185953848092	0.6222417473969426	0.8057269274179146	T	T	T
0.5081683715215731	0.6192129504087327	0.7952005707784608	T	T	T
0.4400738537423841	0.4173858549246839	0.7478818920839541	T	T	T
0.3941964410378330	0.4398078843016091	0.6918479327825385	T	T	T
0.4736027948942266	0.4255382369077770	0.6834587461162219	T	T	T
0.4229480777768089	0.5073436882523724	0.7747694703641692	T	T	T
0.4380639610280842	0.5404649496586612	0.7858103632380762	T	T	T
0.4562778743907818	0.5409921135052675	0.7131463110583591	T	T	T
0.5310336692592779	0.5017535384498729	0.7633858149042912	T	T	T
0.5377753451351330	0.5917327159462623	0.6920580376917342	T	T	T
0.3655505349667617	0.5775505294965469	0.7188487445743890	T	T	T
0.4638041807069622	0.6173177671726715	0.7753889240978125	T	T	T
0.4392464759295040	0.4440467349190932	0.7108946698112583	T	T	T

:::::::::::::

I (VASP)

:::::::::::::

E(PBE, VASP) = -2629.95255319 (eV)

Edisp /kcal,au,eV: -615.1629 -0.98032431 -26.6759829

header

1.00000000000000000

22.0272006988999998	0.0000000000000000	0.0000000000000000
0.0000000000000000	22.0272006988999998	0.0000000000000000
0.0000000000000000	0.0000000000000000	25.0000000000000000

Si	O	H	W	C
90	207	68	1	5

Selective dynamics

Direct

0.8922238835139111	0.1018854006082081	0.5760581185263058	T	T	T
0.8904811936848828	0.4344307355185352	0.5758713563095607	T	T	T
0.8894657579717404	0.7683547056089808	0.5762518951953947	T	T	T

0.5571139076718207	0.1003167256713472	0.5766841998778650	T	T	T
0.5562345226348059	0.4339789982921792	0.5741532675451504	T	T	T
0.5547276282650956	0.7681925079167943	0.5769265385182415	T	T	T
0.2249770261925798	0.0983256374456539	0.5746820951518384	T	T	T
0.2230280256193356	0.4319378663425356	0.5740319405952776	T	T	T
0.2221598075731810	0.7648530266810694	0.5752173455388512	T	T	T
0.7886914866140821	0.2016689111340317	0.5727797196426690	T	T	T
0.7878931704675262	0.5352809619738410	0.5727314740716312	T	T	T
0.7866655544862096	0.8689739804700084	0.5732020405928283	T	T	T
0.4546424443115264	0.2013791471156561	0.5714886968821312	T	T	T
0.4542232423076389	0.5337840144824747	0.5701437302603262	T	T	T
0.4518316566681723	0.8688158512768401	0.5718663663551247	T	T	T
0.1228830308257695	0.1996699672295952	0.5698890637540607	T	T	T
0.1212011646295696	0.5328701400948952	0.5694722654567030	T	T	T
0.1200274457009654	0.8667671105909349	0.5705638983387734	T	T	T
0.8375285550997853	0.3229263207143492	0.5147486018332713	T	T	T
0.8363648246545665	0.6567615004671675	0.5147637816110904	T	T	T
0.8371666142491495	0.9904554214763786	0.5166513487261051	T	T	T
0.0038677358337390	0.1523219058089152	0.5086119134415893	T	T	T
0.0019893285116322	0.4854752698803422	0.5087703937635967	T	T	T
0.0009212348386066	0.8191467105824944	0.5088370114230385	T	T	T
0.5047310474594885	0.3217486492329136	0.5121523603195337	T	T	T
0.5031907419885642	0.6558148031028362	0.5131497672493562	T	T	T
0.5040290545267064	0.9891210546747414	0.5140634040991519	T	T	T
0.6693150902783517	0.1547577536272192	0.5119531288342299	T	T	T
0.6685973931907832	0.4885766769336460	0.5116343093437158	T	T	T
0.6668502968313752	0.8220207850256874	0.5121181992233621	T	T	T
0.1715752583666038	0.3202371799221666	0.5098555971074017	T	T	T
0.1699765286675938	0.6537495347655166	0.5101537123265176	T	T	T
0.1707931771335635	0.9873309370626393	0.5118712736689635	T	T	T
0.3362933345680000	0.1532464987711097	0.5097410291958486	T	T	T
0.3346922533549172	0.4865062630511826	0.5094035162736354	T	T	T
0.3332478606964814	0.8201421679729015	0.5100896121441066	T	T	T
0.9252931479857907	0.2479518950073754	0.4412057999999988	F	F	F
0.9234721060137190	0.5812809469992928	0.4411998089999969	F	F	F
0.9216511250117136	0.9146088959913143	0.4411938189999987	F	F	F

0.7529111499777699	0.0692349000150614	0.4404208059999988	F	F	F
0.7510890959842271	0.4025638999803931	0.4404148160000005	F	F	F
0.7492681149822289	0.7358919379987086	0.4404087959999998	F	F	F
0.5919740800133155	0.2461308839970187	0.4390428069999999	F	F	F
0.5901531579838561	0.5794588920069828	0.4390367869999992	F	F	F
0.5883311029915461	0.9127878550180029	0.4390307959999973	F	F	F
0.4195911590101247	0.0674138810145806	0.4382577840000010	F	F	F
0.4177701179914166	0.4007419050047858	0.4382517929999992	F	F	F
0.4159491659989953	0.7340708370086944	0.4382458030000009	F	F	F
0.2586551010217377	0.2443099019962531	0.4368798140000010	F	F	F
0.2568331360090852	0.5776379110049774	0.4368738229999991	F	F	F
0.2550121249987285	0.9109659190149415	0.4368678029999984	F	F	F
0.0862721130104802	0.0655929070039392	0.4360948200000010	F	F	F
0.0844511320084749	0.3989208939944362	0.4360888000000003	F	F	F
0.0826301130080012	0.7322489020043932	0.4360828100000020	F	F	F
0.8370211119890811	0.1602299059805716	0.3748038109999996	F	F	F
0.8352000710157768	0.4935579000078292	0.3747977909999989	F	F	F
0.8333781360114756	0.8268868919830368	0.3747918010000006	F	F	F
0.5037021039879122	0.1584088799887411	0.3726407890000019	F	F	F
0.5018801090123119	0.4917368889974725	0.3726347980000000	F	F	F
0.5000591280103066	0.8250648379894940	0.3726287779999993	F	F	F
0.1703821270029735	0.1565868999946147	0.3704777960000030	F	F	F
0.1685611460009753	0.4899158779871158	0.3704718050000011	F	F	F
0.1667401190103774	0.8232439160054312	0.3704658150000029	F	F	F
0.6731271740190010	0.3229198749868942	0.3590378170000008	F	F	F
0.6713061330003001	0.6562489269788117	0.3590317959999965	F	F	F
0.6694851519982947	0.9895768760162298	0.3590258059999982	F	F	F
0.3398081360094807	0.3210988939848889	0.3568748240000019	F	F	F
0.3379871249991311	0.6544268729852689	0.3568687739999987	F	F	F
0.3361651000151724	0.9877559539867704	0.3568627829999969	F	F	F
0.0064891129814413	0.3192778830199359	0.3547118010000005	F	F	F
0.0046671389799045	0.6526058909844963	0.3547058110000023	F	F	F
0.0028461179818962	0.9859338999932277	0.3546997899999980	F	F	F
0.9212151169991074	0.0815368740018627	0.2959358100000031	F	F	F
0.9193941349983419	0.4148648979920679	0.2959297900000024	F	F	F
0.9175731539963365	0.7481939200210306	0.2959237990000005	F	F	F

0.5878961680158810	0.0797158999912213	0.2937727870000018	F	F	F
0.5860751269971729	0.4130438570187636	0.2937667970000035	F	F	F
0.5842531320215656	0.7463728790023225	0.2937608060000017	F	F	F
0.7602561120186380	0.2375698979880525	0.2928088009999996	F	F	F
0.7584350709999299	0.5708978769884254	0.2928027809999989	F	F	F
0.7566142079884131	0.9042268990173881	0.2927967910000007	F	F	F
0.2545771300063606	0.0778948739993837	0.2916097940000029	F	F	F
0.2527551349853567	0.4112229049809457	0.2916038039999975	F	F	F
0.2509341240203966	0.7445508839813257	0.2915978130000028	F	F	F
0.4269371030187088	0.2357488869776958	0.2906458080000007	F	F	F
0.4251161220167035	0.5690768959864272	0.2906397880000000	F	F	F
0.4232951100075866	0.9024049039963842	0.2906337980000018	F	F	F
0.0936181169903705	0.2339268619937371	0.2884827849999994	F	F	F
0.0917971359883651	0.5672559139856546	0.2884767950000011	F	F	F
0.0899751189945306	0.9005838629776761	0.2884708050000029	F	F	F
0.9023353429270718	0.1076951153249086	0.6406997676098115	T	T	T
0.9002288239336089	0.4395761160140922	0.6405953000562390	T	T	T
0.8985480243550596	0.7726983611787539	0.6411222488996284	T	T	T
0.5642630954779819	0.1028580340092637	0.6417953041408597	T	T	T
0.5611946678811145	0.4379596065333167	0.6395929169473852	T	T	T
0.5596381410136675	0.7686982460073039	0.6422429164157533	T	T	T
0.2316303043869650	0.1001681265897333	0.6398558604284549	T	T	T
0.2266411474757908	0.4332385894267475	0.6392418730996194	T	T	T
0.2284359235956172	0.7645320208653248	0.6404190913631013	T	T	T
0.7860519917093590	0.2096844635291722	0.6378219098893145	T	T	T
0.7846874270296733	0.5421017629465182	0.6378732964531193	T	T	T
0.7817062063229314	0.8742011747959632	0.6383334923493369	T	T	T
0.4507188012107099	0.2097698417883825	0.6363843081534981	T	T	T
0.4473349983389884	0.5377522885750594	0.6351876591619751	T	T	T
0.4459999519559290	0.8768760955925685	0.6367161539372973	T	T	T
0.1194918244362540	0.2096148692555583	0.6346579723836887	T	T	T
0.1183958016851066	0.5424308749410823	0.6343404316915142	T	T	T
0.1147122894531553	0.8761565692561403	0.6353342973460648	T	T	T
0.8407884134301180	0.1514957374338974	0.5591950004541343	T	T	T
0.8393697699337720	0.4845283688311308	0.5591864541573509	T	T	T
0.8383668336484107	0.8184279884670824	0.5596409313631417	T	T	T

0.5054190355844952	0.1493496933277214	0.5597311684008018	T	T	T
0.5051353011793410	0.4827488645191489	0.5549181275512982	T	T	T
0.5029569532455667	0.8171638709186000	0.5600925136296010	T	T	T
0.1742811206873044	0.1484298063551031	0.5577568576432306	T	T	T
0.1722871472938480	0.4816011000765132	0.5561171382360161	T	T	T
0.1714171099718821	0.8153446078989052	0.5592674353023267	T	T	T
0.8689539418053417	0.0335392399576082	0.5613608713010542	T	T	T
0.8673871653665870	0.3663847465651618	0.5602491423005831	T	T	T
0.8664636551565512	0.7005367728945069	0.5598446886112156	T	T	T
0.5369238408882568	0.0322619012106278	0.5581246161517711	T	T	T
0.5366329023477001	0.3658059227495304	0.5561719648784281	T	T	T
0.5354571210816745	0.7010468502930154	0.5557207893375343	T	T	T
0.2039709046908690	0.0306078332953333	0.5556616802163278	T	T	T
0.2037301296904521	0.3645873767443683	0.5533328826598501	T	T	T
0.2016619818577462	0.6979622296421129	0.5540510588654319	T	T	T
0.9555122453929243	0.1132244322682360	0.5438567361676266	T	T	T
0.9539390826485517	0.4464320827158519	0.5441499148948572	T	T	T
0.9533961825140699	0.7807251995933830	0.5453267553757897	T	T	T
0.6214772519128505	0.1160329151583872	0.5477247109114523	T	T	T
0.6219217176226832	0.4493091297514116	0.5479561924515115	T	T	T
0.6202719681806167	0.7850532817661601	0.5504826617276836	T	T	T
0.7218314132289720	0.1820818053761404	0.5508435420922694	T	T	T
0.7212432753619802	0.5164526384099857	0.5497756824412021	T	T	T
0.7206605509163865	0.8510498778253452	0.5483613596433377	T	T	T
0.2897523572784132	0.1136977363031331	0.5461508995762948	T	T	T
0.2890884784748837	0.4486680778979705	0.5479901585756042	T	T	T
0.2872124587837317	0.7818400125583838	0.5477792823252510	T	T	T
0.8077458043865845	0.2644692579003278	0.5433719186367686	T	T	T
0.8072210541233756	0.5983814174495725	0.5441275788064113	T	T	T
0.8073713081278490	0.9327417280691499	0.5464754906001469	T	T	T
0.3885583529859645	0.1825658702703105	0.5476420377496768	T	T	T
0.3886151046408713	0.5166865587320840	0.5447982389438979	T	T	T
0.3861551895925993	0.8504049007278633	0.5467614013054297	T	T	T
0.4762377819048237	0.2635377583348143	0.5421626703280771	T	T	T
0.4758147827443184	0.5987891272269628	0.5465098181429884	T	T	T
0.4746152547133336	0.9311597226911716	0.5438402670740179	T	T	T

0.0566165360428495	0.1788749799814704	0.5476923135262989	T	T	T
0.0546624988545237	0.5124182201287490	0.5476145851495127	T	T	T
0.0543672795760707	0.8466337694418784	0.5465373854960377	T	T	T
0.1432049526624571	0.2616634021045282	0.5396788135533033	T	T	T
0.1413505932847822	0.5952000910081239	0.5398094727377217	T	T	T
0.1416971823325075	0.9289836858934142	0.5414898039688492	T	T	T
0.7837257842486378	0.0273333089362739	0.4862463060188286	T	T	T
0.7833340811298655	0.3594393990033171	0.4845694132376314	T	T	T
0.7819736691552049	0.6925968651587258	0.4842951030207004	T	T	T
0.9669123919272254	0.2088884505949584	0.4823581299451558	T	T	T
0.9651469960250582	0.5417627091525171	0.4820543761748263	T	T	T
0.9636678651340010	0.8747058374837402	0.4815508289213643	T	T	T
0.4499955585354071	0.0259722669434729	0.4845425224695218	T	T	T
0.4497245812377020	0.3579382801757286	0.4828643980519376	T	T	T
0.4479301584997272	0.6904114749869845	0.4826130259952562	T	T	T
0.6324339251112199	0.2093664054177299	0.4826298837923647	T	T	T
0.6310623904495540	0.5424257840220793	0.4821830773437148	T	T	T
0.6292355257763319	0.8750120101327612	0.4816118994391944	T	T	T
0.1165437515387663	0.0243580214263233	0.4826883111263042	T	T	T
0.1161818976241215	0.3561438832840697	0.4808173492023009	T	T	T
0.1148685119198717	0.6895611225425416	0.4806464935403075	T	T	T
0.2984752954777805	0.2072084351608748	0.4804911174206867	T	T	T
0.2968240966085197	0.5399042528916066	0.4798736062975017	T	T	T
0.2955024983144625	0.8729716472594999	0.4795219500452022	T	T	T
0.8902767090539121	0.3024161774477777	0.4726787674562425	T	T	T
0.8889790274847359	0.6358586565808175	0.4727420032235540	T	T	T
0.8883590076598336	0.9689807638849280	0.4734951339332787	T	T	T
0.5560606602297415	0.3013599904287602	0.4688104176525522	T	T	T
0.5540480743817282	0.6336368388910620	0.4699780145674983	T	T	T
0.5542100515023445	0.9679524511120704	0.4699236647884082	T	T	T
0.7003187849349380	0.1113800846763582	0.4673105547425678	T	T	T
0.6987086184086014	0.4442181328573901	0.4672890154518637	T	T	T
0.6964571351975070	0.7762763789792348	0.4683487156672829	T	T	T
0.2227933235568008	0.2997005893407427	0.4663842853570308	T	T	T
0.2215515462807742	0.6329204394979803	0.4670431218663291	T	T	T
0.2208730216639769	0.9662835052362811	0.4675524443904818	T	T	T

0.3672393944672708	0.1100427869630053	0.4649208661816144	T	T	T
0.3646237293697506	0.4415618485526641	0.4652750236662961	T	T	T
0.3637945579234023	0.7751867986601011	0.4661859506792344	T	T	T
0.0342751034884218	0.1093558876766440	0.4630765122902613	T	T	T
0.0325135966854153	0.4417844515271169	0.4636693395870230	T	T	T
0.0306686218375186	0.7747180896819084	0.4639098365281689	T	T	T
0.8040411469935194	0.1112479120019287	0.4128897789999968	F	F	F
0.8022201659915140	0.4445758760208278	0.4128837880000020	F	F	F
0.8003991839907485	0.7779049280127381	0.4128777979999967	F	F	F
0.8755741120097511	0.2052448690053339	0.4126187860000030	F	F	F
0.8737530709910430	0.5385729069782528	0.4126137789999973	F	F	F
0.8719320889902775	0.8719009159869842	0.4126077889999991	F	F	F
0.4707220790210442	0.1094268860100911	0.4107268149999967	F	F	F
0.4689011279819937	0.4427549239830100	0.4107207950000031	F	F	F
0.4670801160182734	0.7760828730204352	0.4107148049999978	F	F	F
0.5422551040085821	0.2034229039926885	0.4104558229999995	F	F	F
0.5404341220078166	0.5367518660049484	0.4104507859999984	F	F	F
0.5386121269868127	0.8700799349849788	0.4104447960000002	F	F	F
0.1374031309911814	0.1076049130073216	0.4085637929999990	F	F	F
0.1355821489904159	0.4409338830096985	0.4085578019999971	F	F	F
0.1337601240064572	0.7742619509909687	0.4085517820000035	F	F	F
0.2089361399984782	0.2016018780008579	0.4082927999999981	F	F	F
0.2071140999876491	0.5349298719827118	0.4082878230000020	F	F	F
0.2052931489940022	0.8682588930129072	0.4082817729999988	F	F	F
0.9701991080086358	0.2756438849854916	0.3967227939999987	F	F	F
0.9683781859791765	0.6089718939942230	0.3967168029999968	F	F	F
0.9665570859879296	0.9422998429862446	0.3967108129999986	F	F	F
0.7212521429830758	0.0278279130144128	0.3955617849999982	F	F	F
0.7194311020097715	0.3611558970086151	0.3955557940000034	F	F	F
0.7176091670054703	0.6944839359802941	0.3955498039999981	F	F	F
0.6368801000074669	0.2738219200182428	0.3945597709999973	F	F	F
0.6350591180067013	0.6071508530209115	0.3945537809999990	F	F	F
0.6332371229856975	0.9404789210021818	0.3945478199999997	F	F	F
0.3879331649902653	0.0260058919801196	0.3933988209999981	F	F	F
0.3861111099979553	0.3593349160066097	0.3933928009999974	F	F	F
0.3842900989875986	0.6926628950069897	0.3933868109999992	F	F	F

0.3035611509788367	0.2720008789995347	0.3923968080000009	F	F	F
0.3017391259948852	0.6053289180166175	0.3923907880000002	F	F	F
0.2999181449928798	0.9386578799834737	0.3923847969999983	F	F	F
0.0546131130071430	0.0241849160173402	0.3912357990000004	F	F	F
0.0527921389964945	0.3575128910226582	0.3912297789999997	F	F	F
0.0509711169997260	0.6908419130062171	0.3912237879999978	F	F	F
0.7851661440059274	0.1981308910132142	0.3429927829999997	F	F	F
0.7833451630039221	0.5314588549867167	0.3429867919999978	F	F	F
0.7815241219852211	0.8647879359882182	0.3429808019999996	F	F	F
0.4518471059964071	0.1963098939855712	0.3408298189999996	F	F	F
0.4500260949860504	0.5296378729859512	0.3408237700000001	F	F	F
0.4482041000104502	0.8629658819946755	0.3408178090000007	F	F	F
0.1185281349949463	0.1944879000087383	0.3386667970000019	F	F	F
0.1167061180011117	0.5278168919839459	0.3386608060000000	F	F	F
0.1148851360003462	0.8611448999939100	0.3386547859999993	F	F	F
0.8824861050079207	0.1266008759859929	0.3339448270000034	F	F	F
0.8806651230071552	0.4599289000215947	0.3339387770000002	F	F	F
0.8788440819884471	0.7932578329788669	0.3339327870000020	F	F	F
0.9598761199854096	0.0364938789993445	0.3337728079999991	F	F	F
0.9580550790120981	0.3698219059858445	0.3337678019999970	F	F	F
0.9562330839910942	0.7031508679981044	0.3337617809999998	F	F	F
0.5491670970067517	0.1247799100108651	0.3317818049999985	F	F	F
0.5473461750226889	0.4581078590028866	0.3317758139999967	F	F	F
0.5455251340039808	0.7914358970212021	0.3317697940000031	F	F	F
0.6265561579819448	0.0346729049887031	0.3316098150000002	F	F	F
0.6247351170086333	0.3680008650125401	0.3316038250000020	F	F	F
0.6229141350078677	0.7013288740212644	0.3315988180000033	F	F	F
0.2158481329966477	0.1229578849815098	0.3296188119999997	F	F	F
0.2140271070048172	0.4562869070104725	0.3296128209999978	F	F	F
0.2122051419921647	0.7896149160192039	0.3296067710000017	F	F	F
0.2932371499807758	0.0328508839998065	0.3294467930000025	F	F	F
0.2914161090074643	0.3661799130201260	0.3294408020000006	F	F	F
0.2895951570150501	0.6995078920204989	0.3294357959999985	F	F	F
0.7125151159940089	0.2879979009914209	0.3141148090000030	F	F	F
0.7106941339932362	0.6213259100001522	0.3141087890000023	F	F	F
0.7088730930199318	0.9546548720124051	0.3141027989999969	F	F	F

0.3791961069940726	0.2861769199894226	0.3119518159999970	F	F	F
0.3773751259920672	0.6195048689814442	0.3119457960000034	F	F	F
0.3755531310164670	0.9528329370081110	0.3119398059999980	F	F	F
0.0458771439827359	0.2843548950054640	0.3097878100000031	F	F	F
0.0440551229938393	0.6176839469973814	0.3097828029999974	F	F	F
0.0422341449881287	0.9510118959894029	0.3097768129999992	F	F	F
0.8732610939964118	0.0398549059864806	0.2601577940000013	F	F	F
0.8714401720123490	0.3731828630140228	0.2601518029999994	F	F	F
0.8696190710223348	0.7065109009869417	0.2601457829999987	F	F	F
0.5399420859952428	0.0380328859963512	0.2579948010000024	F	F	F
0.5381201509909488	0.3713619110216086	0.2579888109999970	F	F	F
0.5362991090188771	0.7046898600136302	0.2579828200000023	F	F	F
0.8128991129996024	0.2660319209917859	0.2561918199999980	F	F	F
0.8110781309988297	0.5993608830040458	0.2561857999999972	F	F	F
0.8092561359778330	0.9326888920127701	0.2561797799999965	F	F	F
0.2066221390186556	0.0362119119857027	0.2558318080000035	F	F	F
0.2048011119826612	0.3695398869910207	0.2558258179999982	F	F	F
0.2029801460075333	0.7028688790116249	0.2558197979999974	F	F	F
0.4795800749900820	0.2642108800184815	0.2540287969999966	F	F	F
0.4777581389870207	0.5975399020020404	0.2540228069999984	F	F	F
0.4759371280220606	0.9308679100120045	0.2540167869999976	F	F	F
0.7232301240073369	0.1896398960131407	0.2532247899999973	F	F	F
0.7214091420065714	0.5229678750135207	0.2532187999999991	F	F	F
0.7195871469855675	0.8562968369803770	0.2532128100000008	F	F	F
0.9609541890203559	0.1165518979961959	0.2520078120000022	F	F	F
0.9591330889837053	0.4498798549783416	0.2520017920000015	F	F	F
0.9573121669996425	0.7832078929966571	0.2519958020000033	F	F	F
0.1462601129866172	0.2623898980177088	0.2518658039999977	F	F	F
0.1444391460127221	0.5957179069810365	0.2518597839999970	F	F	F
0.1426181350023725	0.9290469290099992	0.2518538240000012	F	F	F
0.3899110849990564	0.1878188850027840	0.2510617969999984	F	F	F
0.3880891499947552	0.5211468940115154	0.2510558070000002	F	F	F
0.3862681090214437	0.8544749020214795	0.2510498170000020	F	F	F
0.6276350619845417	0.1147298810023614	0.2498448039999985	F	F	F
0.6258141400004789	0.4480589029859274	0.2498387839999978	F	F	F
0.6239920850081688	0.7813869119946588	0.2498328089999973	F	F	F

0.0565911229955915	0.1859969050086576	0.2488987890000018	F	F	F
0.0547701489849430	0.5193258519940400	0.2488927989999965	F	F	F
0.0529491269881746	0.8526539210194741	0.2488867940000006	F	F	F
0.2943161430096666	0.1129089000003631	0.2476818109999996	F	F	F
0.2924941479886627	0.4462378920209744	0.2476758059999966	F	F	F
0.2906731369783060	0.7795658710213473	0.2476697860000030	F	F	F
0.9203707995278965	0.7401202323965194	0.6587170646541650	T	T	T
0.5850693997172259	0.0695839617955337	0.6593185651162561	T	T	T
0.2498058338391269	0.7308880808306459	0.6569336905925346	T	T	T
0.7481667047487032	0.2235643857805793	0.6532698363172006	T	T	T
0.0811850813189513	0.2230288744186223	0.6496085903999302	T	T	T
0.0793696462112858	0.5521626357365529	0.6500567984550855	T	T	T
0.9237409978668322	0.0751870177620681	0.6587383616965945	T	T	T
0.9205278392014413	0.4063155578079603	0.6585860886668378	T	T	T
0.5889799465031628	0.4100935374734270	0.6570166310396434	T	T	T
0.5872778413784036	0.7402813999269203	0.6590708738385008	T	T	T
0.2526462014782371	0.0669317892458455	0.6572526223381170	T	T	T
0.2553456279700120	0.4070263728376255	0.6574584978530126	T	T	T
0.7491903507897001	0.5616154163082969	0.6530069703766004	T	T	T
0.7453563531318512	0.8924457410861190	0.6530983381446787	T	T	T
0.4169955526321439	0.2327781145078131	0.6508700420106627	T	T	T
0.4071632488225649	0.8897284179776551	0.6509760021593419	T	T	T
0.0758096320131974	0.8889415933639351	0.6495272453514193	T	T	T
0.5003200770105281	0.3033929169825811	0.2606458070000031	F	F	F
0.4984991549810687	0.6367208959829540	0.2606397870000023	F	F	F
0.4966780839994058	0.9700488450203792	0.2606337960000005	F	F	F
0.9957210419885669	0.1439058930061066	0.2590687869999968	F	F	F
0.9939001800212068	0.4772338869879604	0.2590627969999986	F	F	F
0.9920781249835002	0.8105618359799820	0.2590568070000003	F	F	F
0.1670011130004241	0.3015708919986224	0.2584828139999971	F	F	F
0.1651801469798997	0.6348999139821885	0.2584767940000035	F	F	F
0.1633591199893019	0.9682278630196066	0.2584708030000016	F	F	F
0.6624021530220432	0.1420838830036288	0.2569057939999979	F	F	F
0.6605801580010393	0.4754129049871949	0.2568998039999997	F	F	F
0.6587591169823312	0.8087408539792165	0.2568938140000014	F	F	F
0.3290821310018757	0.1402629020016306	0.2547428009999990	F	F	F

0.3272611199915190	0.4735908810020035	0.2547368110000008	F	F	F
0.3254401389895136	0.8069198730226148	0.2547307910000001	F	F	F
0.8734241129859370	0.0502678940068506	0.2223587929999979	F	F	F
0.8716031309851715	0.3835968970138808	0.2223527880000020	F	F	F
0.8697810769916288	0.7169249060226051	0.2223467979999967	F	F	F
0.5401051640027106	0.0484468750063769	0.2201958149999967	F	F	F
0.5382831100091678	0.3817759160118754	0.2201897950000031	F	F	F
0.5364621280084023	0.7151039240218395	0.2201837900000001	F	F	F
0.2067850979914780	0.0466258970006734	0.2180327920000025	F	F	F
0.2049641310175829	0.3799538610195654	0.2180267869999994	F	F	F
0.2031431200072333	0.7132828830031315	0.2180207970000012	F	F	F
0.7250071169868377	0.2004988790164575	0.2167577889999990	F	F	F
0.7231860760135334	0.5338268879797852	0.2167517990000007	F	F	F
0.7213641410092322	0.8671548959897493	0.2167457939999977	F	F	F
0.3916881680036113	0.1986769140038120	0.2145947960000001	F	F	F
0.3898661140100685	0.5320059059790196	0.2145887909999971	F	F	F
0.3880451620176544	0.8653339149877510	0.2145828009999988	F	F	F
0.0583681089837356	0.1968558880119744	0.2124317880000035	F	F	F
0.0547261130217152	0.8635128740144395	0.2124197930000022	F	F	F
0.0565471309780250	0.5301839110034194	0.2124257979999982	F	F	F
0.8336401580032344	0.3052138690203918	0.2628088000000020	F	F	F
0.8318190570132202	0.6385419370016621	0.2628027800000012	F	F	F
0.8299971220089262	0.9718708990139220	0.2627967889999994	F	F	F
0.4485702490405281	0.4647127144640851	0.8022681344901625	T	T	T
0.4198628959594154	0.4028789704378435	0.7637591549976276	T	T	T
0.4937244753163942	0.4326447662983864	0.7505937729549582	T	T	T
0.3896108046117628	0.6294921405871055	0.6962052993638393	T	T	T
0.3227919193444939	0.5881702422270572	0.6780286352139995	T	T	T
0.3399200671292704	0.6003490699398064	0.7470533597098783	T	T	T
0.4978602089611858	0.5983417626115997	0.7248560437712563	T	T	T
0.4703727332789204	0.5692051998847079	0.7871197221277336	T	T	T
0.5303522307668150	0.5307113639485064	0.7528926171171640	T	T	T
0.2944675460429961	0.4916382996203306	0.7088846622010965	T	T	T
0.3300673044834205	0.4298491140813365	0.7434447528139465	T	T	T
0.3242349565828512	0.5025394791110097	0.7740695177619735	T	T	T
0.3863475634671493	0.3893819188358948	0.6850122734332253	T	T	T

0.3782065696691674	0.4359921289424279	0.6266084026996023	T	T	T
0.4522303203431469	0.4128325338027874	0.6489621956239874	T	T	T
0.4173592970666430	0.5130526543966093	0.7034096893545557	T	T	T
0.4467818610186195	0.4446931345841185	0.7618756903389702	T	T	T
0.3597370141585933	0.5913243393028087	0.7075696323593463	T	T	T
0.4881434607983909	0.5563928156593395	0.7474808473358319	T	T	T
0.3320210452677182	0.4788427522719391	0.7357378009322194	T	T	T
0.4065325717803290	0.4266030609999372	0.6620834760257907	T	T	T

:::::::::::::

H_2 (VASP)

:::::::::::::

E(PBE, VASP) = -6.719431 (eV)

Edisp /kcal,au,eV: -0.0584 -0.00009308 -0.0025327

header

1.00000000000000000

10.0000000000000000	0.0000000000000000	0.0000000000000000
0.0000000000000000	10.0000000000000000	0.0000000000000000
0.0000000000000000	0.0000000000000000	10.0000000000000000

H

2

Direct

0.5373510052685101	0.5000000000000000	0.5000000000000000
0.4626489947314898	0.5000000000000000	0.5000000000000000

:::::::::::::

CH_4

:::::::::::::

Multiplicity: 1

E (PBE/L1, Priroda)= -40.47792299 (a.u.)

Thermal correction to Gibbs free energy= 0.0239514278429 (a.u.)

E (M06/def2-tzvpp, Orca)= -40.494295715906 (a.u.)

H	-0.76043213	-0.31736382	-0.72711378
C	-0.00001891	0.00003662	-0.00000820

H	-0.26087377	-0.38642684	0.99510830
H	0.98091053	-0.39398665	-0.30031706
H	0.04041428	1.09774068	0.03233074

:::::::::::::

H_2

:::::::::::::

Multiplicity: 1

E (PBE/L1, Priroda)= -1.16633010 (a.u.)

Thermal correction to Gibbs free energy= -0.00224805583886 (a.u.)

E (M06/def2-tzvpp, Orca)= -1.171010275108 (a.u.)

H	0.21900118	-0.04709255	0.30312663
H	-0.21900118	0.04709255	-0.30312663

:::::::::::::

II

:::::::::::::

Multiplicity: 1

E (PBE/L1, Priroda)= -17998.87044968 (a.u.)

Thermal correction to Gibbs free energy= 0.139532923253 (a.u.)

E (M06/def2-tzvpp, Orca)= -1913.053839163263 (a.u.)

Si	-1.17865230	0.34092526	0.07461367
O	-1.91077215	-1.13475122	-0.30718540
O	-2.17276241	1.53188161	-0.58681972
O	-1.32048759	0.50165646	1.75201181
O	0.34052660	0.46591267	-0.46039753
Si	-2.88625543	0.41303911	2.36744709
Si	-3.78406408	1.49980277	-0.09967208
Si	-3.50861764	-1.31286364	0.19656745
O	-4.37816818	-0.03456990	-0.48216238
O	-3.52123899	-1.07023910	1.86799744
O	-3.78612444	1.60894331	1.58594686

H	-4.06753043	-2.62656552	-0.17747288
H	-4.57695125	2.57471731	-0.72692881
H	-2.91683027	0.56400029	3.83522047
W	2.23929942	-0.07344678	-0.58897712
C	2.84266754	1.83470117	0.10071722
H	2.58968002	1.86607232	1.17973627
H	3.90475359	2.09649177	-0.01086456
H	2.21232175	2.58938192	-0.40786924
C	3.81440001	-1.01750914	-0.29565375
H	4.59644100	-1.70654855	0.02572226
H	4.17931515	-0.21954245	-1.04989506
C	1.50981695	-1.54581836	0.81728377
H	2.23623085	-2.27482794	1.19432627
H	1.07720500	-0.97884711	1.66264877
H	0.68122745	-2.07378961	0.30922053
C	2.01799719	-0.40208650	-2.66840955
H	2.93708894	-0.53327513	-3.25743343
H	1.40553819	-1.32048876	-2.77183708
H	1.42394553	0.43764374	-3.07788129

:::::::::::::

TS [XVI - XVIII]

:::::::::::::

Multiplicity: 1

E (PBE/L1, Priroda)= -17883.36259113 (a.u.)

Thermal correction to Gibbs free energy= 0.0990416241713 (a.u.)

E (M06/def2-tzvpp, Orca)= -1797.512136203221 (a.u.)

Si	-0.72269461	0.23294646	0.57338785
O	-0.93060340	-0.58072393	-0.89518859
O	-1.43735539	1.74237922	0.38417275
O	-1.64971321	-0.59634087	1.70475084
O	0.84861074	0.30143450	0.98798964
Si	-3.28901676	-0.74359542	1.33175594
Si	-3.06380138	1.72596644	-0.06435228

Si	-2.53374677	-0.72469498	-1.41198400
O	-3.15363013	0.84108379	-1.50115318
O	-3.36818618	-1.50862834	-0.17283934
O	-3.87777253	0.82181746	1.10635379
H	-2.63475770	-1.44283898	-2.69707032
H	-3.61700603	3.08519012	-0.20925381
H	-4.03182967	-1.47951537	2.37182176
W	2.73458308	0.55787664	0.55674573
H	4.25484818	-0.23541287	0.68667834
H	3.89491403	0.88785377	-0.65700834
H	3.19118838	-0.49595805	-0.98215417
H	2.45587977	1.91127804	-0.39413105
C	2.53193005	-1.46468625	-0.87573061
H	2.44996759	-1.96186355	0.10300723
H	1.54651873	-1.32777493	-1.34048977
H	3.18246357	-2.09516362	-1.50105958
H	3.45110940	0.67823849	2.07418783
H	3.76810026	1.87113223	0.82156335

:::::::::::::

XVI (ORCA OPT)

:::::::::::::

Multiplicity: 1

E (PBE/def2-svp, Orca)= -1794.353253259797 (a.u.)

E (M06/def2-tzvpp, Orca)= -1796.344211201802 (a.u.)

Si	-0.597606	-0.438830	0.102229
O	-1.694122	-1.629159	-0.350928
O	-0.919725	0.904885	-0.859157
O	-0.979596	-0.006360	1.682554
O	0.954038	-0.976993	-0.056983
Si	-2.565360	0.529698	1.923368
Si	-2.502431	1.494858	-0.768986
Si	-3.323631	-1.192074	-0.231712
O	-3.508964	0.198692	-1.173961

O	-3.568821	-0.713080	1.370048
O	-2.791721	1.825532	0.862424
H	-4.231053	-2.283224	-0.644486
H	-2.717401	2.672653	-1.635591
H	-2.833728	0.892513	3.330612
W	2.747785	-0.333239	0.182787
H	3.575497	-1.282093	1.342009
H	3.393255	1.180541	-0.188030
H	3.425613	0.495847	1.501029
H	1.962283	1.044816	0.724373
C	3.848029	-0.527591	-1.579030
H	3.669028	-1.575932	-1.926553
H	3.766053	0.177067	-2.428183
H	4.892577	-0.458528	-1.177832

:::::::::::::
XVI
:::::::::::::

Multiplicity: 1
E (PBE/L1, Priroda)= -17882.20059941 (a.u.)
Thermal correction to Gibbs free energy= 0.0834398903621 (a.u.)
E (M06/def2-tzvpp, Orca)= -1796.346769581239 (a.u.)

Si	-0.58482635	-0.43485023	0.09326916
O	-1.67621201	-1.62447804	-0.36634699
O	-0.90855566	0.91357751	-0.85755922
O	-0.95922792	-0.01593504	1.67716661
O	0.95967212	-0.97243615	-0.07396374
Si	-2.54488587	0.51462560	1.91892627
Si	-2.49151566	1.49530123	-0.75465461
Si	-3.30300177	-1.18483586	-0.23706325
O	-3.50008589	0.20685719	-1.17202027
O	-3.55063586	-0.72503065	1.36802181
O	-2.77827815	1.82183286	0.87639450
H	-4.20338218	-2.27558563	-0.65449609

H	-2.70672023	2.67660930	-1.61070602
H	-2.80595066	0.86532288	3.32717545
W	2.72443321	-0.30218366	0.18239141
H	3.52345141	-1.27406048	1.33217389
H	3.37643871	1.19575326	-0.21344705
H	3.39300418	0.52341715	1.50005605
H	1.92729745	1.07215065	0.69939881
C	3.82968966	-0.54590399	-1.56282412
H	3.65392609	-1.59556634	-1.88901339
H	3.75726784	0.13626402	-2.42186785
H	4.86809755	-0.47084557	-1.16101135

:::::::::::::

TS [XVI - XVII] (ORCA MECP)

:::::::::::::

Multiplicity: 1

E (PBE/def2-svp, Orca)= -1794.300887443003 (a.u.)

E (M06/def2-tzvpp, Orca)= -1796.302627815478 (a.u.)

Si	-0.831210	0.118312	-0.133051
O	-1.746116	-1.142650	-0.763570
O	-1.516380	1.544652	-0.694727
O	-1.053258	0.091523	1.533547
O	0.752898	-0.034769	-0.561203
Si	-2.660181	0.219564	2.047661
Si	-3.150331	1.759140	-0.313788
Si	-3.395295	-1.089947	-0.387294
O	-3.956596	0.407530	-0.931800
O	-3.492348	-1.047739	1.299237
O	-3.261677	1.644898	1.368695
H	-4.140942	-2.224551	-0.971749
H	-3.691665	3.031335	-0.835559
H	-2.787046	0.191021	3.519827
W	2.595095	0.253320	-0.264964
H	3.967650	-1.054722	0.125459

H	3.415728	0.985441	-1.571180
H	3.302719	0.224583	1.297989
H	2.588418	1.842648	0.303784
C	4.591350	-1.320541	-0.837823
H	5.181851	-2.129250	-0.358317
H	4.013558	-1.741431	-1.680968
H	5.273779	-0.528366	-1.190206

::::::::::::::

TS [VIII - IX] (ORCA, MECP)

::::::::::::::

Multiplicity: 1

E (PBE/def2-svp, Orca)= -1872.760577297836 (a.u.)

E (M06/def2-tzvpp, Orca)= -1874.895145760453 (a.u.)

Si	-1.351479	-0.127923	-0.331841
O	-2.472877	-0.970280	-1.265984
O	-1.269959	1.425934	-0.986790
O	-2.047622	0.044157	1.196405
O	0.089895	-0.901383	-0.276118
Si	-3.556883	0.799564	1.233641
Si	-2.733883	2.262885	-1.077759
Si	-4.007945	-0.276716	-1.373823
O	-3.783750	1.287533	-1.974600
O	-4.562930	-0.097367	0.213078
O	-3.355752	2.306038	0.493666
H	-4.932076	-1.074734	-2.207937
H	-2.581193	3.612711	-1.661783
H	-4.100782	0.912535	2.604180
W	1.995371	-0.827723	0.068208
C	2.687535	-1.260586	-1.865928
H	3.779282	-1.196672	-2.051816
H	2.348703	-2.324081	-1.992623
H	2.181334	-0.626194	-2.625199
C	3.631900	-1.643121	1.632884

H	4.002287	-2.545329	1.112278
H	4.493254	-1.194222	2.186342
H	2.877162	-1.932273	2.389560
C	1.583380	0.935629	1.249901
H	2.443418	1.492868	1.670681
H	0.908840	1.663506	0.753598
H	1.007055	0.485448	2.101880
H	3.521042	-0.557683	1.014680
H	3.206675	0.327480	-0.228779

:::::::::::::

TS [XII - XVI]

:::::::::::::

Multiplicity: 1

E (PBE/L1, Priroda)= -17922.65547587 (a.u.)

Thermal correction to Gibbs free energy= 0.124372928353 (a.u.)

E (M06/def2-tzvpp, Orca)= -1836.813202014263 (a.u.)

Si	-1.38600047	0.26179640	-0.00987436
O	-2.46404387	-0.91196991	-0.54682696
O	-1.70547077	1.65184968	-0.90190619
O	-1.81308788	0.60664097	1.58165659
O	0.15805879	-0.23433033	-0.13915681
Si	-3.40802998	1.11131389	1.80514892
Si	-3.29464656	2.21334771	-0.81620255
Si	-4.09543181	-0.49187265	-0.44224000
O	-4.28270013	0.93917108	-1.31815497
O	-4.39141931	-0.10836878	1.17532845
O	-3.62862614	2.46449101	0.81999591
H	-4.97765980	-1.56759591	-0.93246702
H	-3.49953245	3.43139313	-1.62232426
H	-3.70767684	1.39569475	3.22125711
W	2.06874895	-0.10589025	-0.20227424
C	3.88039748	-1.24162284	-1.18598701
H	4.69713235	-1.65119317	-0.56778970

H	3.27334943	-2.10148944	-1.51535851
H	4.30173552	-0.74223427	-2.06679598
C	2.47497782	-1.70532642	1.16126894
H	3.49559492	-1.98671326	1.44883911
H	1.92765878	-1.37203891	2.06980114
H	1.95323447	-2.59818684	0.76170170
H	3.87821208	-0.07354725	-0.09664104
H	3.54051349	0.41622923	0.66841365
H	2.05935052	1.40101395	0.56206401
H	2.91041419	1.12037521	-1.02550583
H	2.03494722	-0.12093677	-1.88597010

::::::::::::::

XII (ORCA OPT)

::::::::::::::

Multiplicity: 1

E (PBE/def2-svp, Orca)= -1833.594516897889 (a.u.)

E (M06/def2-tzvpp, Orca)= -1835.654379555605 (a.u.)

Si	-1.039410	-0.041037	-0.192528
O	-2.280283	-0.986752	-0.822072
O	-1.063312	1.408993	-1.049468
O	-1.478085	0.328419	1.391291
O	0.405438	-0.823478	-0.294041
Si	-2.967619	1.106742	1.570305
Si	-2.528550	2.250598	-1.013477
Si	-3.819080	-0.289575	-0.774917
O	-3.699083	1.180837	-1.599391
O	-4.114148	0.099566	0.843023
O	-2.892541	2.500386	0.617644
H	-4.857392	-1.175101	-1.343393
H	-2.477687	3.511307	-1.783711
H	-3.287734	1.401002	2.983161
W	2.248920	-0.481436	0.169503
C	3.401564	-0.696341	-1.582724

H	4.487439	-0.683000	-1.339506
H	3.132247	-1.699747	-1.995432
H	3.230210	0.075742	-2.360683
C	2.894240	-2.044142	1.429057
H	3.995082	-1.991104	1.583376
H	2.423149	-2.069631	2.433095
H	2.647234	-2.987968	0.883281
H	2.887521	0.204150	1.578719
H	3.160569	0.928681	-0.040473
H	1.591310	0.972888	0.709363

:::::::::::::

XV

:::::::::::::

Multiplicity: 1

E (PBE/L1, Priroda)= -17921.47904796 (a.u.)

Thermal correction to Gibbs free energy= 0.110302619837 (a.u.)

E (M06/def2-tzvpp, Orca)= -1835.634371364940 (a.u.)

Si	-1.13017954	0.41378509	0.32334208
O	-1.77239743	-0.23875075	-1.08672433
O	-1.11473662	2.08322693	0.12126012
O	-2.23796849	0.09170574	1.54808924
O	0.34469354	-0.19425470	0.70397749
Si	-3.79770066	0.67645187	1.27331196
Si	-2.61105016	2.77779465	-0.23414190
Si	-3.30491080	0.32869312	-1.50757256
O	-3.18368581	2.00863470	-1.62374560
O	-4.31261554	0.01003160	-0.18971614
O	-3.65293442	2.33932623	1.02122624
H	-3.80341863	-0.28485434	-2.75272004
H	-2.52142919	4.24061126	-0.40007387
H	-4.71166466	0.35667224	2.38627663
W	2.13406462	-0.46724214	0.06312296
C	2.99377834	-2.42259141	0.09887068

H	3.29390828	-2.95973572	-0.80319643
H	2.34744998	-3.04374314	0.74561234
H	1.68789345	-1.12094264	-1.39847512
C	4.07102092	-1.68806694	0.83254610
H	3.92077627	-0.49097849	0.80323538
H	4.14550689	-1.88064962	1.91409858
H	5.05873369	-1.72989606	0.35545938
H	3.10277686	0.92942356	0.18445064
H	3.24470601	-0.36353542	-1.22769583
H	1.80938308	0.62888437	-1.15081802

:::::::::::::

TS [XII - XV]

:::::::::::::

Multiplicity: 1

E (PBE/L1, Priroda)= -17921.41941131 (a.u.)

Thermal correction to Gibbs free energy= 0.102744135645 (a.u.)

E (M06/def2-tzvpp, Orca)= -1835.566353468102 (a.u.)

Si	-1.11165062	0.35313703	0.12800634
O	-1.87336137	-0.10425882	-1.30160971
O	-1.14182396	2.03559353	0.16832158
O	-2.10530537	-0.16805300	1.38387055
O	0.39294108	-0.25767400	0.27056991
Si	-3.69024321	0.40852057	1.33336630
Si	-2.67256025	2.73399167	0.04987162
Si	-3.44458936	0.47634554	-1.50004327
O	-3.35809446	2.15835218	-1.38179190
O	-4.32613557	-0.05462393	-0.16048039
O	-3.59223041	2.09380269	1.31394568
H	-4.04506801	0.03543059	-2.77323821
H	-2.61794864	4.20753976	0.09049761
H	-4.49753868	-0.09098727	2.46277079
W	2.19547752	-0.58932989	-0.35812308
C	2.95453624	-2.39616005	-0.34224359

H	3.76298145	-2.90193541	-0.88504889
H	2.44381838	-3.08387983	0.36606494
H	2.33169899	-1.14014906	-1.95526907
C	3.69565497	-1.07244513	1.35689525
H	4.51433278	-0.34710690	1.33840720
H	2.98301111	-0.84785973	2.17431669
H	4.11982205	-2.06601260	1.56389398
H	3.45817880	0.52933773	-0.36090915
H	3.54537651	-0.53470383	-1.43581964
H	2.07872002	0.62312817	-1.54622154

:::::::::::::

TS [XII - XIV]

:::::::::::::

Multiplicity: 1

E (PBE/L1, Priroda)= -17921.43472570 (a.u.)

Thermal correction to Gibbs free energy= 0.105275369709 (a.u.)

E (M06/def2-tzvpp, Orca)= -1835.584621957372 (a.u.)

Si	-1.13103838	0.02280152	-0.04689868
O	-2.35453279	-0.96941307	-0.64388771
O	-1.13407356	1.40181917	-1.01957622
O	-1.63108873	0.52224229	1.48182550
O	0.30283644	-0.74633594	-0.04226222
Si	-3.12406967	1.30205867	1.53963623
Si	-2.60014792	2.23065940	-1.09689435
Si	-3.88855988	-0.27243683	-0.70175267
O	-3.75206345	1.12284812	-1.64356921
O	-4.25088528	0.23903677	0.86578795
O	-3.02329049	2.62149156	0.48990004
H	-4.90139343	-1.20504697	-1.23235829
H	-2.52086023	3.42291523	-1.96268113
H	-3.49064295	1.70690155	2.91003510
W	2.19412375	-0.52551130	0.38312020
C	3.48482892	-1.06269073	-1.42434843

H	4.57281708	-1.03557439	-1.59142825
H	3.07063425	-1.98074726	-1.87548262
H	3.09660170	-0.16538738	-1.94272022
C	2.97857846	-1.96923348	1.42141603
H	3.53071759	-2.06099232	2.36288458
H	2.75693132	-2.96692690	0.96869453
H	3.72325266	-1.38205854	-0.11038854
H	2.91870200	0.05917068	1.76222831
H	3.41717497	0.65887266	0.27551631
H	1.75544763	1.03153749	0.87320372

:::::::::::::

TS [XII - XIII] (ORCA, MECP)

:::::::::::::

Multiplicity: 1

E (PBE/def2-svp, Orca)= -1833.532619105604 (a.u.)

E (M06/def2-tzvpp, Orca)= -1835.598139696978 (a.u.)

Si	-1.230899	-0.104635	-0.225083
O	-2.468825	-0.911662	-1.035793
O	-1.064023	1.387733	-0.990884
O	-1.816079	0.214249	1.323381
O	0.151097	-0.992354	-0.220100
Si	-3.253941	1.096067	1.394162
Si	-2.458497	2.337448	-1.053597
Si	-3.947500	-0.099986	-1.105259
O	-3.636631	1.398970	-1.822262
O	-4.389397	0.224480	0.493979
O	-2.978285	2.530212	0.543286
H	-4.981156	-0.869183	-1.830933
H	-2.235104	3.630381	-1.735263
H	-3.702670	1.339578	2.781935
W	2.015295	-0.822186	0.258356
C	2.961965	-0.588170	-1.606377
H	4.055797	-0.406098	-1.630414

H	2.735696	-1.564667	-2.110334
H	2.477021	0.232036	-2.180904
C	3.768884	-1.831217	1.735301
H	4.315000	-2.365901	0.938150
H	4.507135	-1.498263	2.499970
H	3.045828	-2.501777	2.235217
H	3.421872	-0.709451	1.513143
H	3.035714	0.524775	0.389034
H	1.671704	0.349622	1.441290

:::::::::::::

TS [VIII - XII]

:::::::::::::

Multiplicity: 1

E (PBE/L1, Priroda)= -17961.94288148 (a.u.)

Thermal correction to Gibbs free energy= 0.151002199133 (a.u.)

E (M06/def2-tzvpp, Orca)= -1876.115395494951 (a.u.)

Si	-1.50087534	0.14502114	0.26639625
O	-2.16574517	-0.95802270	-0.82085477
O	-1.75303453	1.67426417	-0.39908646
O	-2.45481376	0.07082584	1.65135509
O	0.06129257	-0.15196877	0.59232822
Si	-4.09828929	0.36635912	1.41747133
Si	-3.35823408	2.05690797	-0.74684048
Si	-3.79277164	-0.71865938	-1.19276016
O	-3.92691539	0.85573814	-1.78854871
O	-4.63380566	-0.75212730	0.27121750
O	-4.21878957	1.88864319	0.69632915
H	-4.29895762	-1.72136577	-2.14943841
H	-3.49699883	3.40732945	-1.32462819
H	-4.86256438	0.28410981	2.67670317
W	1.90724047	-0.11991292	0.00173687
C	3.22317414	-1.48270933	-1.43365350
H	4.12680721	-2.01171873	-1.08350264

H	2.43098037	-2.24307857	-1.52339102
H	3.41393939	-1.03123000	-2.41395898
C	2.42529476	-1.81335014	1.20732550
H	3.43178218	-2.24988808	1.18087181
H	2.21542438	-1.45276523	2.23611028
H	1.68036790	-2.60030427	0.97638899
C	2.11691642	1.77837340	0.96452699
H	3.06015708	2.33218999	0.89353765
H	1.30526530	2.39815895	0.53353984
H	1.86795913	1.60495376	2.03135743
H	3.64710011	-0.43454414	-0.42709189
H	3.64106572	0.13890241	0.38066367
H	1.31403040	-0.16438788	-1.57852152
H	2.69299772	0.90425587	-1.09558300

:::::::::::::

XI

:::::::::::::

Multiplicity: 1

E (PBE/L1, Priroda)= -17960.76770049 (a.u.)

Thermal correction to Gibbs free energy= 0.135072507649 (a.u.)

E (M06/def2-tzvpp, Orca)= -1874.941988110842 (a.u.)

Si	-0.98410915	0.49355602	0.14852165
O	-1.65960108	-1.02640772	0.42511324
O	-1.31842867	0.86112945	-1.46334810
O	-1.86185757	1.58446078	1.07753495
O	0.60090036	0.55000333	0.50279531
Si	-3.52569273	1.60192122	0.79666882
Si	-2.95380581	0.84384395	-1.88545851
Si	-3.31349465	-1.15231864	0.10943576
O	-3.53000821	-0.69616211	-1.50195713
O	-4.07393959	0.02505520	1.04988382
O	-3.73178131	1.92372841	-0.84830642
H	-3.82937451	-2.50717407	0.38138293

H	-3.16323418	1.17992098	-3.30647720
H	-4.22205823	2.58164590	1.65126457
W	2.46685515	0.04636895	0.58377495
C	1.85793414	-0.77391757	-1.83149761
H	2.27417397	0.26978569	-1.58070812
H	2.56233682	-1.12313205	-2.60013137
H	0.86969308	-0.55176824	-2.26235856
C	1.82372335	-1.65842512	-0.60822584
H	2.51705894	-2.50336035	-0.64336817
H	3.19787062	-1.31616508	1.24228316
H	0.81973263	-1.98922912	-0.30177323
C	2.87750043	0.55972766	2.56239554
H	3.99196357	0.57135647	2.58988012
H	2.51933021	1.59520506	2.74091713
H	2.53874185	-0.10300774	3.37485548
H	3.85010393	-0.54489321	-0.23784580
H	3.39946664	1.25825194	-0.16525133

:::::::::::::

X

:::::::::::::

Multiplicity: 1

E (PBE/L1, Priroda)= -17920.30158122 (a.u.)

Thermal correction to Gibbs free energy= 0.0881310555839 (a.u.)

E (M06/def2-tzvpp, Orca)= -1834.457412971837 (a.u.)

Si	-0.78430642	-0.01353840	0.37730789
O	-1.89745709	-1.23362605	0.07662054
O	-0.94713930	1.11006598	-0.86372153
O	-1.27060663	0.74950691	1.79108105
O	0.73520284	-0.60973156	0.48372329
Si	-2.83967640	1.37571823	1.76136073
Si	-2.49920470	1.75554279	-1.04123817
Si	-3.50230797	-0.71924638	-0.04954046
O	-3.53683384	0.44399541	-1.27256411

O	-3.86209190	0.08418663	1.39056418
O	-2.90658324	2.43549491	0.44877684
H	-4.42334607	-1.84012992	-0.31385238
H	-2.57225725	2.73134674	-2.14441690
H	-3.19912261	2.03132879	3.03226039
W	2.60560757	-0.45139502	0.22556884
C	2.92294595	-1.30436151	-1.70812805
H	3.95196093	-1.37403937	-2.08917269
H	2.51234646	-2.33385136	-1.62345731
H	2.33041821	-0.74230745	-2.45485113
C	3.90037559	-1.24270888	1.27686033
H	4.61967390	-0.78916251	0.51027354
H	4.36814764	-1.81812361	2.07547219
H	2.83208944	1.11018316	0.84540272
H	3.46216490	0.64485246	-0.73432978

:::::::::::::

TS (VIII - X)

:::::::::::::

Multiplicity: 1

E (PBE/L1, Priroda)= -17960.72031839 (a.u.)

Thermal correction to Gibbs free energy= 0.128936129526 (a.u.)

E (M06/def2-tzvpp, Orca)= -1874.885121900881 (a.u.)

Si	-1.21481668	0.05847701	-0.25114939
O	-2.25098671	-1.12739075	-0.85457212
O	-1.44958307	1.41781383	-1.22524368
O	-1.81548429	0.47414382	1.27138255
O	0.32520389	-0.43112570	-0.21894529
Si	-3.42058901	0.98509684	1.30965743
Si	-3.03410789	1.98177816	-1.32394253
Si	-3.87971498	-0.70284919	-0.93155663
O	-3.97467892	0.69286467	-1.87774886
O	-4.34153240	-0.25711122	0.63035494
O	-3.53708985	2.30013180	0.25631087

H	-4.71414097	-1.79558543	-1.46767027
H	-3.15027974	3.16823660	-2.19381667
H	-3.86618263	1.32488326	2.67472071
W	2.19016289	-0.51418109	0.42210363
C	3.09929251	-1.23342050	-1.53043221
H	4.12553287	-1.54188707	-1.78519278
H	2.37835114	-1.94008474	-1.97446848
H	2.97563652	-0.23008387	-1.97924878
C	2.60541333	-2.22022073	1.26114949
H	3.39828991	-1.68772114	-0.21822491
H	3.11718730	-2.52739033	2.18008854
H	2.19345538	-3.09610590	0.70611351
C	1.92916499	1.49446628	1.15532139
H	2.78108563	2.00386831	1.61891815
H	1.54047529	2.10511226	0.31517483
H	1.11768881	1.40815758	1.90888291
H	3.11584373	-0.28229423	1.79515829
H	3.75640295	0.17242147	0.32687537

::::::::::::::

VII

::::::::::::::

Multiplicity: 1

E (PBE/L1, Priroda)= -18000.06295519 (a.u.)

Thermal correction to Gibbs free energy= 0.159782317695 (a.u.)

E (M06/def2-tzvpp, Orca)= -1914.247070083369 (a.u.)

Si	-1.40024095	0.08134576	0.37843776
O	-2.35743263	-1.07554340	-0.38681196
O	-1.61376939	1.51609240	-0.48082298
O	-2.08545845	0.33382020	1.89461100
O	0.16083930	-0.35709713	0.51470380
Si	-3.70689043	0.79850866	1.88828989
Si	-3.21021085	2.04547030	-0.61959552
Si	-3.99441453	-0.68774306	-0.52014871

O	-4.07554409	0.78676635	-1.33881919
O	-4.55019337	-0.39889941	1.04777308
O	-3.80433214	2.20126945	0.95311316
H	-4.76346616	-1.74745826	-1.19961313
H	-3.31432142	3.30315763	-1.38359975
H	-4.23154901	0.99859445	3.25244356
W	1.88194498	-0.87654383	-0.19823340
C	3.41767370	1.90044222	0.29649694
H	2.47812985	2.47710160	0.26842829
H	4.10967303	2.43144900	0.97343096
H	3.85009094	1.91342274	-0.71639102
C	3.16912798	0.46604324	0.76206374
H	2.95567588	-0.27518893	-1.35523631
H	2.79758518	0.44715668	1.81424244
H	4.11944010	-0.12025410	0.75206927
C	2.19989808	-2.68767962	0.83201237
H	1.59008800	-3.51990072	0.43842221
H	3.25590758	-3.01061405	0.78734759
H	1.91778197	-2.50623307	1.89130512
C	0.84044751	-0.64973026	-2.08401118
H	1.42196730	-0.85058085	-2.99097865
H	-0.03275768	-1.33078402	-2.05428434
H	0.46055633	0.39032256	-2.11952570
H	2.51375339	-1.99671251	-1.29711935

::::::::::::::

VI

::::::::::::::

Multiplicity: 1

E (PBE/L1, Priroda)= -17959.59057430 (a.u.)

Thermal correction to Gibbs free energy= 0.113202766446 (a.u.)

E (M06/def2-tzvpp, Orca)= -1873.759788834515 (a.u.)

Si	-1.06470545	0.28336020	-0.17139910
O	-2.26776939	-0.87204883	-0.37056869

O	-1.68682508	1.73285289	-0.74758906
O	-0.84836293	0.47465821	1.48757311
O	0.31483542	-0.13428590	-0.93688633
Si	-2.23965506	0.90808773	2.34327905
Si	-3.12361730	2.23663982	-0.01581256
Si	-3.73684584	-0.51419602	0.38227655
O	-4.23382760	0.98171918	-0.22208662
O	-3.39157425	-0.28223083	2.01877865
O	-2.80798561	2.33365097	1.64002217
H	-4.74348973	-1.57016502	0.16682671
H	-3.61013472	3.51389537	-0.56961313
H	-1.98007126	1.06015393	3.78708625
W	2.14899613	-0.63868290	-0.91136985
C	3.16951183	-1.02290437	-2.39929084
H	3.98825145	-1.19121560	-1.61228375
H	3.47575711	-1.17392457	-3.43380803
C	2.95826761	1.25539180	-0.31980791
H	2.55272156	1.53923227	0.67019074
H	4.05100736	1.36602887	-0.27910268
H	2.56655075	1.96362945	-1.08164872
C	1.89782234	-2.58774763	-0.05547454
H	2.78212806	-3.23439008	0.03278187
H	1.44285898	-2.49823816	0.94950419
H	1.17066433	-3.08504071	-0.73315846
H	3.21549131	-0.84423006	0.38158097

:::::::::::::

TS [IV - VIII]

:::::::::::::

Multiplicity: 1

E (PBE/L1, Priroda)= -18001.22984935 (a.u.)

Thermal correction to Gibbs free energy= 0.178016955635 (a.u.)

E (M06/def2-tzvpp, Orca)= -1915.419881641466 (a.u.)

Si	-1.64367872	0.13057530	-0.03644236

O	-2.64745377	-1.12154596	-0.54794622
O	-1.64307172	1.27091140	-1.28366999
O	-2.43360613	0.86322115	1.26048303
O	-0.15909483	-0.38037065	0.35139592
Si	-3.99285308	1.42009239	0.94485085
Si	-3.16019513	1.84905553	-1.74007180
Si	-4.21834502	-0.67410808	-0.96424063
O	-4.08318700	0.50208142	-2.16888469
O	-4.87836248	0.09531530	0.38626359
O	-3.86885776	2.49646934	-0.35098709
H	-5.02841025	-1.82739754	-1.40065454
H	-3.07374467	2.83645543	-2.83317271
H	-4.61176749	2.04502332	2.12952233
W	1.78512279	-0.26158788	0.34333230
C	3.57742717	-1.72284077	-0.05379557
H	4.25434658	-2.01156119	0.76830848
H	2.95247921	-2.60539109	-0.26993339
H	4.17030604	-1.47547826	-0.94355534
C	1.55786900	1.81107575	0.85896935
H	0.94397050	2.23390503	0.03907182
H	0.94143681	1.84553729	1.77915884
H	2.44468328	2.44354296	0.98939394
C	1.82198358	-1.54789941	2.06541679
H	2.77090835	-1.83956689	2.53314142
H	1.20298436	-1.00199380	2.80689702
H	1.26034879	-2.46011513	1.78660985
C	1.59903795	-0.81822456	-1.72800945
H	2.50277326	-0.96080263	-2.33167656
H	0.99142347	-1.74473489	-1.74907307
H	0.98723216	-0.01005972	-2.17614473
H	2.92930068	0.59389740	-0.57221698
H	3.55645296	-0.30496236	0.78267322
H	3.19254110	0.33148178	1.32498637

::::::::::::::

TS [IV - VII]

:::::::::::::

Multiplicity: 1

E (PBE/L1, Priroda)= -17999.98512937 (a.u.)

Thermal correction to Gibbs free energy= 0.156526485212 (a.u.)

E (M06/def2-tzvpp, Orca)= -1914.171792526024 (a.u.)

Si	-1.51456236	0.24047908	0.26493724
O	-2.01228534	-0.64923079	-1.07925107
O	-1.60715129	1.86166233	-0.17032208
O	-2.68037220	-0.00332874	1.45114975
O	-0.03437623	-0.21407163	0.79913429
Si	-4.25554030	0.41279781	1.01100991
Si	-3.12142247	2.38092208	-0.70356828
Si	-3.55124155	-0.26510920	-1.66091033
O	-3.53891003	1.38669576	-2.00424761
O	-4.61963726	-0.48473884	-0.37320215
O	-4.21399294	2.03198598	0.53577722
H	-3.91603642	-1.07918253	-2.83611275
H	-3.12536134	3.80976353	-1.06854795
H	-5.21839381	0.17255875	2.10210837
W	1.81466068	-0.27212023	0.26138647
C	2.45654123	-2.44422690	-0.21230053
H	3.52272424	-2.23703268	-0.02375570
H	1.98313217	-2.97891931	0.62600070
H	2.41991288	-3.09273741	-1.10462398
C	2.53029433	1.73378612	0.56762916
H	2.42447385	2.41251518	-0.28915506
H	1.81223978	2.04664340	1.36258090
H	3.54137496	1.82753441	0.99312966
C	3.05618564	-0.59667753	2.01745394
H	4.12674807	-0.54203029	1.73288221
H	2.90717559	0.18508069	2.78439931
H	2.91388990	-1.58968629	2.48753456
C	1.40623424	-1.29749004	-1.40576899
H	1.95087752	-1.33668366	-2.35374732

H	0.44935872	-1.85025708	-1.44441466
H	1.11339642	0.31406393	-1.34014070
H	2.98006332	0.11703412	-0.92704453

:::::::::::::

TS [IV - VI]

:::::::::::::

Multiplicity: 1

E (PBE/L1, Priroda)= -18000.01745586 (a.u.)

Thermal correction to Gibbs free energy= 0.157035472973 (a.u.)

E (M06/def2-tzvpp, Orca)= -1914.199194985391 (a.u.)

Si	-1.57881094	0.62613811	0.18891950
O	-2.22170616	-0.90518277	-0.11746857
O	-1.93907368	1.56048136	-1.16783466
O	-2.48250923	1.26756519	1.45621644
O	0.00346397	0.58722477	0.53028635
Si	-4.14600352	1.34882933	1.19615576
Si	-3.57297810	1.65792680	-1.57175874
Si	-3.87166306	-0.94363688	-0.46348188
O	-4.11209068	0.07226374	-1.79054607
O	-4.65819991	-0.22179430	0.84451434
O	-4.37443475	2.25458266	-0.21057951
H	-4.35491111	-2.31463857	-0.71696356
H	-3.80322858	2.49365408	-2.76565296
H	-4.86193098	1.92249005	2.35158088
W	1.85380997	0.07675770	0.24893095
C	3.82757003	-1.23444309	-0.21930832
H	4.43429567	-1.65025900	0.60358161
H	3.25780227	-2.07999925	-0.63409052
H	4.49317476	-0.84590355	-1.00254968
C	3.05865941	1.34397350	1.11262988
H	3.79994356	2.09656227	0.82032956
H	2.88980456	1.30019730	2.21153627
H	3.64690980	0.02807003	0.60724849

C	1.84334301	-1.52534725	1.66823579
H	2.78679522	-1.91246566	2.07480016
H	1.20430326	-1.16065014	2.49768375
H	1.30306918	-2.36421038	1.18443325
C	1.28814277	-0.47145478	-1.75368142
H	2.08261325	-0.85404000	-2.40861085
H	0.51343667	-1.25628060	-1.61191907
H	0.80491139	0.38729405	-2.25444074
H	2.88549193	0.71629528	-0.90819642

:::::::::::::

TS [IV - V] (MECP, ORCA)

:::::::::::::

Multiplicity: 1

E (PBE/def2-svp, Orca)= -1912.014632519949 (a.u.)

E (M06/def2-tzvpp, Orca)= -1914.218803066382 (a.u.)

Si	-1.590177	0.105426	0.361567
O	-2.687611	-1.036166	-0.224296
O	-1.458536	1.279080	-0.858909
O	-2.356640	0.873245	1.653506
O	-0.161059	-0.555719	0.776601
Si	-3.853428	1.573995	1.317310
Si	-2.904735	2.004947	-1.341438
Si	-4.203784	-0.447253	-0.669607
O	-3.928139	0.751916	-1.830103
O	-4.828078	0.345002	0.687041
O	-3.595543	2.666250	0.051919
H	-5.103841	-1.511326	-1.164388
H	-2.706368	3.015016	-2.403993
H	-4.456855	2.219754	2.502985
W	1.732876	-0.530084	0.219340
C	4.083433	-0.836513	-0.226131
H	4.465364	-0.571893	0.779193
H	4.090308	-1.939606	-0.327025

H	4.796240	-0.426453	-0.976682
C	1.982571	1.512274	0.685827
H	1.428057	2.102972	-0.077179
H	1.525550	1.715562	1.682900
H	3.038755	1.856340	0.703926
C	2.220912	-1.990663	1.664072
H	3.281739	-2.217342	1.912707
H	1.645923	-1.808358	2.600776
H	1.778379	-2.912957	1.208235
C	1.241835	-1.206665	-1.724232
H	2.112343	-1.547072	-2.323396
H	0.509644	-2.043614	-1.651420
H	0.750855	-0.359378	-2.252750
H	3.150011	-0.080719	-0.756354

:::::::::::::

TS [VIII - XI]

:::::::::::::

Multiplicity: 1

E (PBE/L1, Priroda)= -17960.69584803 (a.u.)

Thermal correction to Gibbs free energy= 0.131373820755 (a.u.)

E (M06/def2-tzvpp, Orca)= -1874.864459971719 (a.u.)

Si	-1.28719335	-0.32864304	-0.46640565
O	-2.55132404	-1.33043570	-0.96278980
O	-1.27216652	0.95272551	-1.56217229
O	-1.77741199	0.30572443	1.02177222
O	0.09339394	-1.20976634	-0.39476587
Si	-3.24683404	1.13133669	1.00844305
Si	-2.71419114	1.81161704	-1.70189971
Si	-4.06511930	-0.60789756	-1.08071815
O	-3.90516034	0.69804764	-2.14059492
O	-4.41012564	0.04993450	0.43800563
O	-3.11129631	2.35135005	-0.15052492
H	-5.10649864	-1.55579807	-1.52193523

H	-2.61036080	2.92393503	-2.66583197
H	-3.59179579	1.66728414	2.33995495
W	1.84921862	-0.51786078	0.10314338
C	3.13837211	-0.63862781	-1.60184203
H	4.14624340	-0.54370202	-1.13448272
H	3.09968224	-1.61455819	-2.10989375
H	3.05393358	0.18478171	-2.32596469
C	2.59962865	-1.54568329	1.59210221
H	2.24515763	-2.31592075	0.04574709
H	3.66485877	-1.80109295	1.68107781
H	1.96468016	-2.06013800	2.32830405
C	2.60475995	0.32644206	2.14355882
H	3.48770780	0.91392740	1.85785057
H	1.69870223	0.95230416	2.22380897
H	2.77823955	-0.11852346	3.13750889
H	2.39189411	1.03784403	-0.30912785
H	0.83300517	0.88139358	0.20767190

:::::::::::::

III

:::::::::::::

Multiplicity: 1

E (PBE/L1, Priroda)= -18039.33186541 (a.u.)

Thermal correction to Gibbs free energy= 0.188361008414 (a.u.)

E (M06/def2-tzvpp, Orca)= -1953.535102732988 (a.u.)

Si	-1.95178863	-0.02479055	-0.22551539
O	-3.03529960	-1.31021748	-0.24891532
O	-2.42889707	1.02203562	-1.45485993
O	-2.21428212	0.79814159	1.22257417
O	-0.41526896	-0.52349326	-0.39125956
Si	-3.79535558	1.33716983	1.46694694
Si	-4.02017791	1.57452516	-1.35920696
Si	-4.66044843	-0.88669769	-0.08447264
O	-5.00512741	0.20350280	-1.32679435

O	-4.78921570	-0.02346800	1.36131705
O	-4.18057623	2.31854209	0.14844796
H	-5.54853810	-2.06404863	-0.11374226
H	-4.36022822	2.48284899	-2.47059618
H	-3.95125759	2.04459037	2.75204699
W	1.49480259	-0.51589840	-0.24602015
C	3.81271240	-1.21545885	-0.96677174
H	4.70353711	-1.09689641	-0.33177349
H	3.09171495	-1.77752784	-0.24185945
H	3.96258048	-1.96663108	-1.75507878
C	1.22897497	1.62716154	-0.24380376
H	0.76261548	1.81644314	-1.23328498
H	0.48984576	1.89070624	0.53650012
H	2.11342468	2.26613935	-0.13588003
C	1.33779893	-2.03223557	1.27082441
H	2.23165507	-2.44719891	1.75582404
H	0.64289006	-1.67467872	2.05265116
H	0.81621136	-2.84392150	0.71662455
C	2.87976884	0.20466271	1.33172968
H	2.17617475	0.55243521	2.11410782
H	3.50693961	-0.57853709	1.78659993
H	3.51590953	1.06004122	1.05638732
C	3.22574140	0.08545701	-1.43544983
H	3.01943784	0.13437776	-2.51271626
H	1.57351295	-1.40936147	-1.70173913
H	3.77021279	0.97228080	-1.09284195

:::::::::::::

TS [I - III]

:::::::::::::

Multiplicity: 1

E (PBE/L1, Priroda)= -18039.26586797 (a.u.)

Thermal correction to Gibbs free energy= 0.181241713412 (a.u.)

E (M06/def2-tzvpp, Orca)= -1953.469034687284 (a.u.)

Si	-1.79456933	-0.13321265	-0.02461588
O	-2.93770983	-1.23464636	0.52878916
O	-2.18980475	0.20192180	-1.62444767
O	-2.05471063	1.29320081	0.84091468
O	-0.27655630	-0.70361354	0.16173698
Si	-3.61517347	1.93066522	0.72068682
Si	-3.75420072	0.77980999	-1.87869488
Si	-4.54489363	-0.73855811	0.39449619
O	-4.80524298	-0.37312537	-1.23282114
O	-4.66888902	0.71932824	1.23956306
O	-3.91514728	2.16386144	-0.92293050
H	-5.48485919	-1.75744611	0.89814441
H	-4.02533715	1.05025551	-3.30290140
H	-3.76430773	3.17472471	1.49983871
W	1.62551496	-0.44086986	-0.06338263
C	3.52154955	0.58098758	-0.48969565
H	3.93006337	1.19598343	0.33005039
H	4.23643718	-0.24082394	-0.69759001
H	3.48962320	1.20047908	-1.39961979
C	1.37102770	1.35232724	0.84753281
H	0.99661254	1.21148110	-0.68109303
H	0.40755415	1.59811411	1.31999210
H	2.04257546	2.21573112	0.80716601
C	2.16874709	-2.45829057	0.49671267
H	3.27847452	-2.51819625	0.47260501
H	1.87024094	-2.73545739	1.52533902
H	1.79721372	-3.23537768	-0.19552617
C	2.13548160	0.22951897	2.16511687
H	1.41037999	-0.41243739	2.68636569
H	3.12501677	-0.25020373	2.11481146
H	2.26887948	1.15873311	2.74236100
C	2.05071338	-1.08219681	-2.05632386
H	2.84971054	-1.83658645	-2.15254934
H	1.08368490	-1.60474381	-2.25035969
H	2.17190100	-0.30133742	-2.81967139

:::::::::::::

VIII (OPT, ORCA)

:::::::::::::

Multiplicity: 1

E (PBE/def2-svp, Orca)= -1872.819354323577 (a.u.)

E (M06/def2-tzvpp, Orca)= -1874.950483672561 (a.u.)

Si	-1.153811	-0.044769	-0.215529
O	-2.244825	-1.137558	-0.888029
O	-1.311122	1.383774	-1.100533
O	-1.724391	0.297505	1.335601
O	0.367941	-0.646888	-0.225578
Si	-3.304534	0.891446	1.420063
Si	-2.867007	2.041421	-1.158870
Si	-3.855052	-0.630129	-0.934897
O	-3.869402	0.832117	-1.783627
O	-4.283554	-0.256356	0.657418
O	-3.346557	2.271985	0.445567
H	-4.747714	-1.643202	-1.537812
H	-2.923548	3.288724	-1.951197
H	-3.731125	1.165638	2.809158
W	2.273879	-0.546238	0.141855
C	3.127927	-0.956856	-1.739117
H	4.201718	-1.223538	-1.619189
H	2.574570	-1.839097	-2.142603
H	3.074208	-0.124265	-2.470089
C	2.617462	-2.292568	1.268188
H	3.706520	-2.519189	1.299249
H	2.259074	-2.250041	2.317120
H	2.087458	-3.115196	0.729905
C	1.689803	1.406371	0.910183
H	2.489850	2.040455	1.327637
H	1.198185	1.945221	0.069204
H	0.922214	1.223000	1.695351
H	3.251057	-0.134684	1.466534

H	3.520776	0.572918	-0.125964

:::::::::::::

VIII

:::::::::::::

Multiplicity: 1

E (PBE/L1, Priroda)= -17960.78106153 (a.u.)

Thermal correction to Gibbs free energy= 0.134534198113 (a.u.)

E (M06/def2-tzvpp, Orca)= -1874.953895574318 (a.u.)

Si	-1.13585400	-0.03218521	-0.20707888
O	-2.20610847	-1.14003517	-0.88327878
O	-1.30197713	1.39053494	-1.09478458
O	-1.71465680	0.30375339	1.33948107
O	0.38265155	-0.62075932	-0.21165278
Si	-3.30071208	0.87725506	1.40865824
Si	-2.86532500	2.02377966	-1.15908076
Si	-3.81876186	-0.64733825	-0.93683013
O	-3.85115719	0.80819948	-1.79221261
O	-4.26526349	-0.28240961	0.65028596
O	-3.35611366	2.25847230	0.43928258
H	-4.69130357	-1.67172243	-1.54119699
H	-2.92928271	3.26661464	-1.95104953
H	-3.73352336	1.14725630	2.79276016
W	2.27537208	-0.51973324	0.15376835
C	3.08370116	-0.95655323	-1.73589332
H	4.14737126	-1.24585694	-1.63180546
H	2.51036585	-1.81816149	-2.13968850
H	3.04404358	-0.12420060	-2.45916751
C	2.57511902	-2.28384308	1.25508894
H	3.65320233	-2.53414476	1.28216299
H	2.22681281	-2.23797452	2.30113893
H	2.02753565	-3.09111016	0.72343056
C	1.72087313	1.42886787	0.92418607
H	2.52999237	2.04368092	1.33439004

H	1.23529803	1.97640266	0.09234041
H	0.95855601	1.25983929	1.71012759
H	3.26348531	-0.14547803	1.47118657
H	3.53565916	0.56684952	-0.13456863

::::::::::::::

IV (OPT, ORCA)

::::::::::::::

Multiplicity: 1

E (PBE/def2-svp, Orca)= -1912.040961535818 (a.u.)

E (M06/def2-tzvpp, Orca)= -1914.244908922796 (a.u.)

Si	-1.429804	0.512447	0.191195
O	-2.074781	-0.957646	-0.332652
O	-1.733826	1.624301	-1.041179
O	-2.367120	0.997516	1.503440
O	0.151971	0.409901	0.589921
Si	-4.024512	1.136045	1.209260
Si	-3.353639	1.799609	-1.488590
Si	-3.715725	-0.934455	-0.737413
O	-3.895799	0.258091	-1.921224
O	-4.531369	-0.369531	0.630855
O	-4.190368	2.216330	-0.080240
H	-4.206890	-2.255358	-1.184099
H	-3.538905	2.789425	-2.570864
H	-4.776994	1.564522	2.407367
W	1.996942	-0.125134	0.281801
C	3.242377	-1.773324	-0.445401
H	4.167246	-1.624405	0.166413
H	2.845945	-2.777685	-0.183094
H	3.538408	-1.769676	-1.510209
C	2.987886	1.564929	1.054308
H	2.932773	2.464303	0.407455
H	2.505099	1.787762	2.035342
H	4.062574	1.332649	1.224453

C	2.009377	-1.508196	1.907384
H	2.971946	-1.970140	2.196938
H	1.626380	-0.884287	2.750189
H	1.268310	-2.314056	1.718544
C	1.227235	-0.367857	-1.736674
H	1.942295	-0.675187	-2.518359
H	0.407164	-1.119206	-1.675359
H	0.776963	0.611373	-2.013903
H	3.178846	0.356940	-0.835604

:::::::::::::

XVIII

:::::::::::::

Multiplicity: 1

E (PBE/L1, Priroda)= -17842.91254693 (a.u.)

Thermal correction to Gibbs free energy= 0.0545259115247 (a.u.)

E (M06/def2-tzvpp, Orca)= -1757.049037282196 (a.u.)

Si	-0.04777391	-0.15427588	0.15268309
O	-0.91577890	-1.54495877	-0.18269783
O	-0.20020708	0.86486661	-1.16809057
O	-0.78465877	0.59713453	1.45397822
O	1.52815787	-0.50314069	0.47857669
Si	-2.41658571	0.98147502	1.20809333
Si	-1.79928412	1.26421459	-1.56252849
Si	-2.55511640	-1.28188632	-0.52146531
O	-2.60136729	-0.19895619	-1.81498600
O	-3.18810170	-0.46666514	0.81367917
O	-2.46990572	1.94905449	-0.17357738
H	-3.26708710	-2.53988199	-0.80880190
H	-1.87248095	2.16081764	-2.72989324
H	-3.01126022	1.63961775	2.38505892
W	3.37401279	-0.46477954	0.42103188
H	4.02272599	-0.30642724	-1.13199540
H	4.06023758	0.32216564	1.74917551

H	4.03431781	1.05369916	0.08012818
H	4.04061228	-1.88085502	-0.21443908
H	4.06954354	-1.49121868	1.56607021

:::::::::::::

I - H_2 complex

:::::::::::::

Multiplicity: 1

E (PBE/L1, Priroda)= -18040.50254910 (a.u.)

Thermal correction to Gibbs free energy= 0.204155246048 (a.u.)

E (M06/def2-tzvpp, Orca)= -1954.709670408645 (a.u.)

Si	-1.87993802	0.12377673	0.11130032
O	-2.68647933	-1.28692198	-0.33725785
O	-1.99377485	1.16796739	-1.21093486
O	-2.79614627	0.81835330	1.34037128
O	-0.35031216	-0.15534121	0.55955211
Si	-4.40702497	1.13645352	0.95640705
Si	-3.56185818	1.50451324	-1.73474391
Si	-4.29162469	-1.08600667	-0.81367943
O	-4.28075602	0.01884649	-2.09109386
O	-5.08759325	-0.33025314	0.46924754
O	-4.39084488	2.13449810	-0.40595383
H	-4.92514428	-2.36457350	-1.18871314
H	-3.57764427	2.42277193	-2.88946895
H	-5.13813609	1.74420815	2.08439333
W	1.55625612	-0.34430337	0.26473504
C	2.90439904	0.66125496	-1.21997604
H	3.65669923	1.33575925	-0.78555772
H	3.40255345	-0.00920361	-1.93680043
H	2.18782292	1.28062759	-1.79205257
C	1.54647164	1.74652685	0.81915141
H	0.86099837	2.26147648	0.12068766
H	1.07773134	1.76327868	1.82323445
H	2.50171536	2.28390825	0.85547410

C 1.47917171 -1.97617332 1.67422245
H 2.38972260 -2.53237858 1.93152734
H 1.03921971 -1.55928699 2.59991134
H 0.73173229 -2.67582259 1.25046406
C 3.43647650 -0.06638496 1.58902845
H 2.92743667 0.25189072 2.51463813
H 4.05154736 -0.94147857 1.85957814
H 4.09688561 0.74175999 1.24701062
C 1.00380667 -1.23084104 -1.63592231
H 1.78248685 -1.53526820 -2.34716799
H 0.37781687 -2.10862180 -1.38229273
H 0.34810353 -0.49226979 -2.13380340
H 2.77321135 -1.58546829 -0.36250609
H 3.23501205 -1.11727400 0.19699030

:::::::::::::
I
:::::::::::::

Multiplicity: 1
E (PBE/L1, Priroda)= -18039.33895457 (a.u.)
Thermal correction to Gibbs free energy= 0.186795480622 (a.u.)
E (M06/def2-tzvpp, Orca)= -1953.542673071299 (a.u.)

Si -1.77385264 -0.08191448 -0.04921380
O -2.83551257 -1.36049401 -0.30678920
O -1.93528998 0.94699766 -1.37438563
O -2.36013359 0.76396246 1.28291835
O -0.25190772 -0.61924756 0.15737183
Si -3.94810664 1.31979133 1.14354716
Si -3.50064981 1.51208794 -1.66050141
Si -4.44975131 -0.92311864 -0.53400519
O -4.47877928 0.14781148 -1.83946572
O -4.90557839 -0.03429463 0.82710635
O -4.00076285 2.28137086 -0.24351790
H -5.31666147 -2.09622878 -0.75315743

H	-3.56312594	2.40380151	-2.83391143
H	-4.38966961	2.04932197	2.34706304
W	1.66609921	-0.47315991	0.15473136
C	3.24211780	0.55483485	-0.94962657
H	3.63206460	1.48323666	-0.51117500
H	4.07659869	-0.17510524	-0.99485350
H	2.93173975	0.75159068	-1.99120630
C	1.16002188	1.60656862	0.52581932
H	0.58929495	1.92216740	-0.37121411
H	0.47880665	1.63956385	1.39666482
H	1.98844266	2.30625808	0.68637040
C	1.91229788	-2.21877283	1.35686179
H	2.89747886	-2.67135623	1.13966392
H	1.81642213	-2.09617479	2.44600977
H	1.12576413	-2.91484761	1.00502848
C	3.10844154	0.12683213	1.69495674
H	2.53547225	0.12999133	2.64315095
H	3.90447835	-0.63227384	1.80138341
H	3.57963120	1.11174011	1.57496681
C	1.78579591	-1.53435281	-1.69884669
H	2.79211803	-1.84844624	-2.01504067
H	1.15721213	-2.43388606	-1.54315466
H	1.32948320	-0.94425527	-2.51354929

:::::::::::::

TS [I - II]

:::::::::::::

Multiplicity: 1

E (PBE/L1, Priroda)= -18039.29183920 (a.u.)

Thermal correction to Gibbs free energy= 0.182832419684 (a.u.)

E (M06/def2-tzvpp, Orca)= -1953.489840580106 (a.u.)

Si	-1.91813734	-0.14580477	0.05395829
O	-3.03854970	-1.40058460	0.01524196
O	-2.09217911	0.68672126	-1.40382439

O	-2.43147048	0.91151884	1.26145995
O	-0.40874343	-0.68896858	0.29399357
Si	-4.00101783	1.50406087	1.08769073
Si	-3.64352366	1.26713240	-1.72440002
Si	-4.64140308	-0.93545472	-0.22688069
O	-4.67823761	-0.06601838	-1.67435390
O	-5.01914703	0.15981752	1.00142357
O	-4.06813758	2.25568048	-0.42325050
H	-5.55838392	-2.09089039	-0.24677260
H	-3.71470217	1.98090403	-3.01371863
H	-4.37538400	2.41911034	2.18264637
W	1.51959593	-0.58142260	0.22689440
C	3.77045884	-0.09860560	-0.49536853
H	4.47485434	0.22501347	0.28432068
H	4.28079586	-0.78291368	-1.19355384
H	3.47953287	0.78300447	-1.08507953
C	1.26125688	1.56834234	0.24208622
H	0.67895422	1.71218039	-0.69476641
H	0.64372177	1.92634573	1.08232641
H	2.18230878	2.16408248	0.19228536
C	2.29899636	-2.16145077	1.05855476
H	3.19716725	-1.31381495	0.22291385
H	2.92768893	-2.47269568	1.90089801
H	1.89984272	-3.00966965	0.45676119
C	2.59086359	0.18338868	2.01311197
H	1.71469427	0.13232370	2.69970778
H	3.38213044	-0.45184986	2.44035587
H	2.94539488	1.22326069	1.98377408
C	1.61964884	-1.16109130	-1.84389591
H	2.52939993	-1.64398350	-2.22495590
H	0.75851076	-1.84211838	-1.99573128
H	1.43319947	-0.25555031	-2.45385290

::::::::::::::

IX

::::::::::::::

Multiplicity: 3

E (PBE/L1, Priroda)= -17920.28963336 (a.u.)

Thermal correction to Gibbs free energy= 0.0858853901071 (a.u.)

E (M06/def2-tzvpp, Orca)= -1834.456157318391 (a.u.)

Si	-0.88200678	-0.00149301	-0.13853195
O	-2.14004778	-0.93151241	-0.75251180
O	-0.88536771	1.44225134	-1.00732714
O	-1.30832542	0.39791710	1.44170494
O	0.53581615	-0.79683793	-0.23325315
Si	-2.78475132	1.19778665	1.61035496
Si	-2.33848599	2.30008922	-0.97465074
Si	-3.66238496	-0.20443738	-0.70515249
O	-3.52894243	1.25054606	-1.55139928
O	-3.95352699	0.20205302	0.90750774
O	-2.69433980	2.58434972	0.65111981
H	-4.71160636	-1.08026691	-1.25988953
H	-2.26636883	3.54798191	-1.75797604
H	-3.09111334	1.51105011	3.01888883
W	2.41420178	-0.70101745	0.12481440
C	3.64133318	-0.61642809	-1.56168252
H	4.68689237	-0.54459187	-1.19005917
H	3.57363170	-1.49549519	-2.23289989
H	3.44858979	0.30149247	-2.15333871
C	3.12714496	-2.09253990	1.50763343
H	4.21485212	-1.89252285	1.62421324
H	2.66613770	-1.93917686	2.50448374
H	3.00086534	-3.15340565	1.21399875
H	2.93780259	0.71420791	0.91395257

:::::::::::::

XIII

:::::::::::::

Multiplicity: 3

E (PBE/L1, Priroda)= -17880.98830573 (a.u.)

Thermal correction to Gibbs free energy= 0.0604742478327 (a.u.)

E (M06/def2-tzvpp, Orca)= -1795.144415368516 (a.u.)

Si	-0.22332396	0.35870240	0.38103834
O	-0.80095026	-0.65757644	-0.83168727
O	-0.56693690	1.92958011	-0.10947088
O	-1.15635913	0.06189654	1.74495877
O	1.36249647	0.13059638	0.69443594
Si	-2.81905661	0.27154050	1.53407773
Si	-2.19648256	2.24412245	-0.42263974
Si	-2.44485304	-0.48785116	-1.18447087
O	-2.68139927	1.13161042	-1.59693511
O	-3.27317989	-0.74418371	0.26391834
O	-3.03946050	1.85304523	0.98698472
H	-2.88274574	-1.40792948	-2.25086409
H	-2.42442557	3.63901496	-0.84329766
H	-3.57322235	-0.00481507	2.77085474
W	3.09406302	-0.17501024	-0.02982226
C	3.81274337	-2.11027072	0.01011221
H	3.31119250	-2.83561141	-0.65862380
H	4.82003679	-1.88702140	-0.42841421
H	3.97161723	-2.56103618	1.00846605
H	4.30968980	0.86030220	0.56929671
H	3.40055660	0.39089462	-1.60791767

:::::::::::::

V

:::::::::::::

Multiplicity: 3

E (PBE/L1, Priroda)= -17959.58692057 (a.u.)

Thermal correction to Gibbs free energy= 0.110318396226 (a.u.)

E (M06/def2-tzvpp, Orca)= -1873.764635449868 (a.u.)

Si	-1.21049660	0.10434924	0.32468554

O	-1.95354733	-1.29255691	-0.25831888
O	-1.23558907	1.20895346	-0.95348543
O	-2.23194286	0.72319185	1.50906493
O	0.28157071	-0.16477794	0.89741448
Si	-3.81201855	1.03877480	1.01165917
Si	-2.76260018	1.55179715	-1.58287939
Si	-3.51913521	-1.09013074	-0.85079719
O	-3.42642333	0.07708431	-2.06812573
O	-4.42670636	-0.41002411	0.40002849
O	-3.70324990	2.10410667	-0.29435533
H	-4.09892996	-2.35658248	-1.33741541
H	-2.70333112	2.52700801	-2.68867030
H	-4.64077499	1.58058010	2.10518255
W	2.15517643	-0.33589366	0.47570944
C	3.22686053	1.46035446	0.63000893
H	2.86519056	2.22108355	-0.09203592
H	3.18220417	1.90390135	1.64510122
H	4.29653245	1.27240378	0.39755212
C	3.09806136	-1.71640391	1.74083239
H	4.17630491	-1.77835445	1.48310634
H	3.02914083	-1.42632427	2.80885411
H	2.67518607	-2.73617045	1.63454521
C	2.60470928	-1.01946332	-1.45658589
H	3.70733729	-1.09270746	-1.57216301
H	2.18676517	-2.02523521	-1.66471347
H	2.23970569	-0.32896384	-2.24419897

:::::::::::::

XVII

:::::::::::::

Multiplicity: 3

E (PBE/L1, Priroda)= -17841.68240670 (a.u.)

Thermal correction to Gibbs free energy= 0.0352530596634 (a.u.)

E (M06/def2-tzvpp, Orca)= -1755.828256369339 (a.u.)

Si	0.38932980	-0.34518240	0.22210424
O	-0.58390371	-1.64354418	-0.20058701
O	0.33340002	0.74757064	-1.05374715
O	-0.33615613	0.40249166	1.53785488
O	1.91501322	-0.83213661	0.56252510
Si	-1.92553701	0.91783619	1.27599770
Si	-1.21888503	1.28386240	-1.46020893
Si	-2.18648774	-1.24203868	-0.56244634
O	-2.12293562	-0.09959420	-1.80369850
O	-2.79648496	-0.44555400	0.79499511
O	-1.87533144	1.95285185	-0.05667484
H	-2.98349887	-2.42732768	-0.92787844
H	-1.19844715	2.23762491	-2.58436422
H	-2.50138252	1.56233248	2.47045840
W	3.77761582	-0.58923285	0.46459997
H	4.28570656	0.99391657	0.09095528
H	4.50557269	-1.56238016	-0.73243706
H	4.52241205	-0.91149593	1.96255182

:::::::::::::
Benchmarking and computation for ^{1}H magnetic shielding/chemical shift calculations
:::::::::::::

:::::::::::::
$WH_6(PPhPr^{i}_2)_3$ (Ph = phenyl, Pr^i = isopropyl), based on crystal **IPPHWH01** (ADF)
:::::::::::::
E(PBE, ADF) = −1147.45122455 (eV)
Solvent (COSMO) = toluene

W	4.830704	2.642537	11.746959
P	4.481230	0.550893	10.313071
P	3.111023	3.782231	13.079390
P	7.251172	3.418505	11.420440
H	4.923488	3.175658	10.088046
H	5.850594	1.421276	12.414769
H	3.250441	2.653374	11.026648
H	3.943225	1.483930	12.685074

H	4.924258	4.379779	11.682874
H	5.463974	3.086269	13.293892
C	5.736287	0.056656	9.036205
C	4.221394	-1.067566	11.246494
C	2.916285	0.621644	9.241374
C	1.741149	2.655184	13.760799
C	3.648242	4.705871	14.630038
C	2.129358	5.108807	12.209392
C	8.555471	2.268228	10.758870
C	7.522868	4.961357	10.370121
C	8.070175	3.923806	13.057639
C	6.058942	-1.272771	8.712961
C	6.371649	1.082114	8.317841
H	4.112064	-1.829846	10.461577
C	2.934462	-1.081085	12.080276
C	5.430683	-1.436194	12.113788
H	2.135760	0.862451	9.981044
C	2.970003	1.770996	8.227252
C	2.554176	-0.692678	8.540443
H	2.336891	1.832514	14.189669
C	0.835651	3.229673	14.857839
C	0.885138	2.083855	12.624977
H	2.718320	5.122915	15.043497
C	4.259815	3.775088	15.684091
C	4.588570	5.870497	14.308421
C	2.182610	5.212188	10.809685
C	1.298480	6.008872	12.903311
C	9.663313	2.688767	10.001971
C	8.466499	0.906923	11.092612
H	8.607292	5.138686	10.416047
C	7.131843	4.758307	8.901779
C	6.828847	6.203341	10.944047
H	7.303256	4.572479	13.508950
C	8.264618	2.714582	13.981432
C	9.378540	4.711087	12.923611
C	6.995367	-1.562810	7.716491

H	5.590370	-2.104802	9.236938
C	7.296988	0.795831	7.312159
H	6.142553	2.116796	8.572161
H	2.034575	-0.910518	11.474574
H	2.826561	-2.063271	12.565968
H	2.969681	-0.311731	12.863544
H	6.360217	-1.487230	11.530986
H	5.568680	-0.696105	12.913926
H	5.266842	-2.421910	12.576431
H	3.196140	2.727467	8.713440
H	3.734276	1.584067	7.457905
H	1.997455	1.856707	7.718428
H	2.359873	-1.515118	9.241408
H	1.639346	-0.547079	7.945088
H	3.349918	-1.009497	7.849636
H	1.387815	3.579160	15.739815
H	0.215185	4.056118	14.483140
H	0.147525	2.439477	15.196892
H	1.505201	1.614239	11.854028
H	0.192191	1.326830	13.023875
H	0.284481	2.874295	12.149729
H	3.588242	2.951844	15.964864
H	5.195474	3.333724	15.312722
H	4.485548	4.350399	16.595408
H	4.141368	6.580065	13.598782
H	4.834283	6.417364	15.232232
H	5.523690	5.498938	13.869754
C	1.417339	6.159795	10.123644
H	2.843072	4.539144	10.263562
C	0.533633	6.959295	12.220482
H	1.240926	5.987919	13.990915
C	10.639232	1.777692	9.586854
H	9.781487	3.733932	9.718932
C	9.445899	-0.003544	10.690703
H	7.605827	0.564558	11.667893
H	7.672872	3.922544	8.438534

H	6.053753	4.563713	8.813560
H	7.364340	5.669594	8.329045
H	7.088526	6.388958	11.994749
H	7.132297	7.088884	10.364108
H	5.736923	6.106190	10.881546
H	7.339977	2.132697	14.084591
H	9.054338	2.050666	13.598936
H	8.573754	3.061256	14.979617
H	9.247711	5.678803	12.421709
H	9.782037	4.915499	13.927736
H	10.140832	4.136647	12.375668
C	7.618812	-0.530077	7.012201
H	7.237542	-2.602737	7.492985
H	7.782348	1.611918	6.774758
C	0.584333	7.034378	10.825884
H	1.475363	6.215303	9.035600
H	-0.103256	7.642544	12.783655
C	10.535469	0.427746	9.930212
H	11.484251	2.128541	8.992725
H	9.349414	-1.056134	10.960924
H	8.356267	-0.757400	6.241199
H	-0.015497	7.773135	10.292793
H	11.294732	-0.283534	9.602138

::::::::::::::

W[($Me_3SiNCH_2CH_2)_3N](C_5H_8$)(H) (Me = methyl), based on crystal **ZAGKEK** (ADF)

::::::::::::::

E(PBE, ADF) = −984.51669442 (eV)

Solvent (COSMO) = toluene

W	1.445328	4.428940	4.563460
N	-0.456591	4.812032	5.160294
N	2.217614	6.015720	3.580516
N	2.234846	3.411314	6.149911
N	1.766886	6.091881	6.297562
C	2.196090	3.081063	3.322961

H	0.358417	3.759611	3.492710
Si	-2.011138	4.395024	4.437412
C	-0.620736	5.598294	6.403602
Si	2.266009	6.131527	1.810925
C	2.436448	7.295455	4.284147
Si	1.839109	1.787575	6.694853
C	3.048434	4.192914	7.092720
C	2.745703	7.049511	5.747589
C	2.276651	5.431736	7.522214
C	0.428894	6.690956	6.501040
C	1.723373	2.112782	2.250088
C	3.709069	2.800665	3.501595
C	-2.817080	5.963730	3.735263
C	-3.153378	3.710381	5.794873
C	-1.979973	3.096049	3.070520
H	-0.562065	4.938536	7.289165
H	-1.611620	6.080957	6.450344
C	3.798156	5.343360	1.038146
C	0.699496	5.492197	0.970544
C	2.351102	7.970567	1.315473
H	1.561379	7.965771	4.192961
H	3.285441	7.849138	3.852006
C	3.364410	0.658410	6.734159
C	0.483070	0.983954	5.666229
C	1.180966	1.867625	8.479613
H	4.007612	4.490790	6.629297
H	3.324648	3.610916	7.987906
H	3.740189	6.588469	5.835167
H	2.753331	7.992177	6.325135
H	1.418956	5.120900	8.132393
H	2.887763	6.134720	8.115508
H	0.254858	7.424686	5.703155
H	0.381793	7.218607	7.470957
C	2.769957	0.983500	2.203868
H	1.708614	2.611285	1.259782
H	0.699342	1.752105	2.422163

C	4.086919	1.732297	2.451765
H	3.881355	2.415221	4.522364
H	4.334357	3.705233	3.419848
H	-3.826277	5.745840	3.349487
H	-2.215805	6.367003	2.905519
H	-2.914962	6.754841	4.495573
H	-2.735780	2.791022	6.235570
H	-4.135802	3.457880	5.365243
H	-3.323682	4.426460	6.612965
H	-1.498600	2.164205	3.399664
H	-1.469026	3.441940	2.162296
H	-3.026822	2.865298	2.808623
H	4.716184	5.742383	1.497705
H	3.822061	5.592773	-0.035818
H	3.812539	4.249674	1.133171
H	0.545395	4.417663	1.131311
H	0.754806	5.682621	-0.114126
H	-0.181508	6.022835	1.363685
H	1.510337	8.555895	1.718379
H	2.295482	8.026507	0.216157
H	3.286751	8.462291	1.621629
H	4.190090	1.123788	7.295858
H	3.731337	0.436050	5.720845
H	3.123767	-0.298730	7.225676
H	0.248106	-0.007674	6.087795
H	0.776938	0.856487	4.615908
H	-0.431784	1.593298	5.690130
H	0.928086	0.850526	8.820421
H	0.265366	2.476888	8.536803
H	1.908997	2.278708	9.195694
H	2.761128	0.418791	1.259571
H	2.586161	0.266807	3.021776
H	4.419678	2.209058	1.515709
H	4.904813	1.078532	2.789858

:::::::::::::

W[($Me_3SiNCH_2CH_2)_3N]H_3$, based on crystal **ZAGKEY01** (ADF)

:::::::::::::

E(PBE, ADF) = −918.00819764 (eV)

Solvent (COSMO) = benzene

W	2.792610	4.109041	6.332873
N	2.023876	2.595760	7.434546
N	2.330961	5.974583	6.965772
N	4.687924	3.845741	5.672712
N	3.996414	4.268522	8.267810
H	2.767698	4.500095	4.671895
H	2.080987	3.030327	5.219074
H	1.318430	4.513510	5.576262
Si	1.045724	1.294617	6.747573
C	2.492850	2.414522	8.818871
Si	0.955629	6.895146	6.346870
C	2.993793	6.501343	8.170844
Si	5.155187	3.975392	3.973573
C	5.781567	3.794543	6.658137
C	3.149776	3.684546	9.336515
C	4.256849	5.712864	8.480490
C	5.243181	3.495893	8.048788
C	2.082492	0.138521	5.666451
C	0.371738	0.225397	8.171820
C	-0.464792	1.920989	5.808333
H	3.201557	1.567088	8.889151
H	1.661155	2.164055	9.496698
C	0.984116	7.093018	4.471014
C	1.034932	8.658145	7.061120
C	-0.690445	6.163476	6.924818
H	2.310437	6.476711	9.041248
H	3.277370	7.558583	8.044552
C	4.167356	2.825631	2.851024
C	6.974620	3.442249	3.801055
C	5.065695	5.761886	3.358598
H	6.349290	4.744718	6.669254

H	6.515452	3.012493	6.405276
H	2.375139	4.423652	9.579390
H	3.745693	3.496386	10.246664
H	5.051940	6.007381	7.782927
H	4.613589	5.897210	9.508946
H	4.983724	2.431420	8.119377
H	5.986107	3.723417	8.833138
H	2.939739	-0.255455	6.235667
H	2.475331	0.663231	4.783610
H	1.480479	-0.719621	5.326338
H	1.171163	-0.250156	8.761130
H	-0.250765	-0.581005	7.751813
H	-0.261996	0.803299	8.863117
H	-1.080319	2.561645	6.458698
H	-1.080768	1.062203	5.494639
H	-0.189911	2.502723	4.918487
H	0.868022	6.134028	3.948877
H	1.935656	7.543783	4.149150
H	0.166629	7.765528	4.162824
H	0.962336	8.675673	8.159753
H	0.189438	9.244468	6.666392
H	1.960830	9.180972	6.773087
H	-0.717325	6.094056	8.024239
H	-0.841776	5.153134	6.518196
H	-1.531736	6.800342	6.606169
H	3.101581	3.087717	2.820908
H	4.253464	1.785299	3.201210
H	4.573737	2.875205	1.827354
H	7.134491	2.401883	4.125810
H	7.662151	4.085255	4.372045
H	7.269475	3.504783	2.741293
H	4.033756	6.140520	3.382284
H	5.444044	5.836548	2.326154
H	5.682620	6.419090	3.992215

::::::::::::::

[(≡Si-O-)WH_5], model ‘A’ (ADF)

:::::::::::::

E(PBE, ADF) = −752.90331024 (eV)

Si	3.076270	6.939910	3.835000
Si	2.106790	9.273590	5.811220
Si	3.769020	11.769330	4.856300
O	-0.314030	10.988240	3.574570
O	4.273740	5.972180	4.422250
O	3.264200	11.827590	3.311580
O	1.673560	6.139660	3.717550
O	2.812730	10.769280	5.721860
O	5.255400	11.001060	4.708210
O	0.478900	9.463690	5.586540
O	3.506550	7.288820	2.308150
O	2.851050	8.268640	4.746510
O	2.317960	8.652260	7.281900
O	-1.048330	8.500240	3.669040
Si	-0.780570	9.802270	4.611190
O	-2.200070	10.127390	5.446110
O	3.658100	13.342560	5.441000
H	5.915255	11.122943	5.407311
H	3.422365	13.415142	6.379295
H	3.826417	11.384309	2.661101
H	4.306915	5.798672	5.375073
H	0.524675	11.449309	3.721034
H	-2.395434	11.072575	5.539449
H	-0.399566	7.780526	3.705701
H	1.641761	5.412210	3.079825
H	4.445200	7.453544	2.135509
W	2.859509	7.894493	8.859057
H	1.678253	7.940460	10.061116
H	3.150926	9.021079	10.084243
H	4.493208	8.164533	9.195968
H	2.105769	6.417139	9.170141
H	3.844279	6.542747	8.602198

:::::::::::::

[(≡Si-O-)WH_5], model ‘B’ (i.e., very slightly modified ‘XVIII’ structure) (ADF)

:::::::::::::

E(PBE, ADF) = −684.41028345 (eV)

Si	-0.0512	-0.1549	0.1527
O	-0.9144	-1.5280	-0.1674
O	-0.2036	0.8424	-1.1588
O	-0.7792	0.5990	1.4315
O	1.5120	-0.4963	0.4662
Si	-2.3865	0.9829	1.1901
Si	-1.7747	1.2381	-1.5545
Si	-2.5299	-1.2721	-0.5032
O	-2.5694	-0.2096	-1.7882
O	-3.1482	-0.4488	0.8071
O	-2.4337	1.9249	-0.1851
H	-3.2415	-2.5259	-0.7757
H	-1.8469	2.1212	-2.7244
H	-2.9760	1.6469	2.3580
W	3.3334	-0.4577	0.4166
H	3.9813	-0.2524	-1.0812
H	4.0143	0.2563	1.7311
H	3.9885	1.0269	0.1462
H	4.0012	-1.8028	-0.2518
H	4.0244	-1.4904	1.4908

:::::::::::::

[(≡Si-O-)$WH_3(Me)_2$], model ‘A’ (Me = methyl) (ADF)

(note that model ‘B’ is identical to ‘XII’ and hence not presented again)

:::::::::::::

E(PBE, ADF) = −785.96782623 (eV)

Si	3.076270	6.939910	3.835000
Si	2.106790	9.273590	5.811220
Si	3.769020	11.769330	4.856300
O	-0.314030	10.988240	3.574570

O	4.273740	5.972180	4.422250
O	3.264200	11.827590	3.311580
O	1.673560	6.139660	3.717550
O	2.812730	10.769280	5.721860
O	5.255400	11.001060	4.708210
O	0.478900	9.463690	5.586540
O	3.506550	7.288820	2.308150
O	2.851050	8.268640	4.746510
O	2.317960	8.652260	7.281900
O	-1.048330	8.500240	3.669040
Si	-0.780570	9.802270	4.611190
O	-2.200070	10.127390	5.446110
O	3.658100	13.342560	5.441000
H	5.857217	11.010461	5.467741
H	3.388046	13.410606	6.370443
H	3.797010	11.341382	2.666847
H	4.292356	5.786720	5.373135
H	0.559536	11.392956	3.683533
H	-2.368885	11.071034	5.592243
H	-0.431917	7.756190	3.748803
H	1.637163	5.426044	3.064506
H	4.445094	7.451663	2.133489
W	2.709277	7.727742	8.824812
C	4.763895	8.202347	9.327716
H	5.083134	7.716841	10.264818
H	5.458245	7.865465	8.540330
H	4.868717	9.288982	9.482388
C	1.615794	5.860481	8.711516
H	0.538343	6.074015	8.617258
H	1.951029	5.251820	7.854967
H	1.741894	5.240916	9.614363
H	1.440459	7.677587	9.930224
H	3.732142	6.405667	8.636861
H	2.800177	8.711315	10.190161

:::::::::::::

[(≡Si-O-)WH_3(=CH_2)] (ADF)

::::::::::::::

E(PBE, ADF) = −761.33209353 (eV)

Si	3.076270	6.939910	3.835000
Si	2.106790	9.273590	5.811220
Si	3.769020	11.769330	4.856300
O	-0.314030	10.988240	3.574570
O	4.273740	5.972180	4.422250
O	3.264200	11.827590	3.311580
O	1.673560	6.139660	3.717550
O	2.812730	10.769280	5.721860
O	5.255400	11.001060	4.708210
O	0.478900	9.463690	5.586540
O	3.506550	7.288820	2.308150
O	2.851050	8.268640	4.746510
O	2.317960	8.652260	7.281900
O	-1.048330	8.500240	3.669040
Si	-0.780570	9.802270	4.611190
O	-2.200070	10.127390	5.446110
O	3.658100	13.342560	5.441000
H	5.777137	10.870922	5.514284
H	3.355967	13.402950	6.360825
H	3.723037	11.243603	2.691219
H	4.301079	5.819663	5.378936
H	0.536579	11.428545	3.720243
H	-2.384551	11.072620	5.557899
H	-0.407322	7.774986	3.728360
H	1.628079	5.446155	3.043859
H	4.447525	7.435410	2.132730
W	3.282397	8.248866	8.887361
C	4.664355	8.440749	10.117099
H	4.067345	7.549380	10.572281
H	5.547478	8.711691	10.692314
H	2.059105	8.502053	10.037003
H	3.781643	6.692311	8.419588
H	4.178806	9.558601	8.370882

8. Supporting references:

(1) M. K. Samantary, E. Callens, E. Abou-Hamad, A. J. Rossini, C. M. Widdifield, R. Dey, L. Emsley and J. M. Basset, *J. Am. Chem. Soc.*, 2013, **136**, 1054.

(2) D. N. Laikov and Y. A. Ustynyuk, *Russ. Chem. B+*, 2005, **54**, 820.

(3) D. N. Laikov, *Chem. Phys. Lett.*, 2005, **416**, 116.

(4) K. G. Dyall, *J. Chem. Phys.*, 1994, **100**, 2118.

(5) Y. Zhao and D. G. Truhlar, *Theor. Chem. Acc.*, 2008, **120**, 215.

(6) F. Neese, *Wires. Comput. Mol. Sci.*, 2012, **2**, 73.

(7) F. Weigend and R. Ahlrichs, *Phys. Chem. Chem. Phys.*, 2005, **7**, 3297.

(8) F. Weigend, *Phys. Chem. Chem. Phys.*, 2006, **8**, 1057.

(9) D. Andrae, U. Haussermann, M. Dolg, H. Stoll and H. Preuss, *Theor. Chim. Acta*, 1990, **77**, 123.

(10) X. Rozanska, F. Delbecq and P. Sautet, *Phys. Chem. Chem. Phys.*, 2010, **12**, 14930.

(11) V. D'Elia, H. Dong, A. J. Rossini, C. M. Widdifield, S. V. Vummaleti, Y. Minenkov, A. Poater, E. Abou-Hamad, J. D. Pelletier, L. Cavallo, L. Emsley and J. M. Basset, *J. Am. Chem. Soc.*, 2015, **137**, 7728.

(12) G. Kresse and D. Joubert, *Phys. Rev. B*, 1999, **59**, 1758.

(13) G. Kresse and J. Furthmuller, *Phys. Rev. B*, 1996, **54**, 11169.

(14) G. Kresse and J. Furthmuller, *Comp. Mater. Sci.*, 1996, **6**, 15.

(15) G. Kresse and J. Hafner, *Phys. Rev. B*, 1994, **49**, 14251.

(16) G. Kresse and J. Hafner, *Phys. Rev. B*, 1993, **48**, 13115.

(17) P. E. Blochl, *Phys. Rev. B*, 1994, **50**, 17953.

(18) S. Grimme, J. Antony, S. Ehrlich and H. Krieg, *J. Chem. Phys.*, 2010, 132, 154104.

(19) G. te Velde, F. M. Bickelhaupt, E. J. Baerends, C. F. Guerra, S. J. A. Van Gisbergen, J. G. Snijders and T. Ziegler, *J. Comput. Chem.*, 2001, **22**, 931.

(20) C. F. Guerra, J. G. Snijders, G. te Velde and E. J. Baerends, *Theor. Chem. Acc.*, 1998, **99**, 391.

(21) E. van Lenthe, A. Ehlers and E. J. Baerends, *J. Chem. Phys.*, 1999, **110**, 8943.

(22) E. van Leenthe, E. J. Baerends and J. G. Snijders, *J. Chem. Phys.*, 1994, **101**, 9783.

(23) E. van Lenthe, E. J. Baerends and J. G. Snijders, *J. Chem. Phys.*, 1993, **99**, 4597.

(24) E. van Lenthe, J. G. Snijders and E. J. Baerends, *J. Chem. Phys.*, 1996, **105**, 6505.

(25) J. P. Perdew, K. Burke and M. Ernzerhof, *Phys. Rev. Lett.*, 1996, **77**, 3865.

(26) J. P. Perdew, K. Burke and M. Ernzerhof, *Phys. Rev. Lett.*, 1997, **78**, 1396.

(27) D. Gregson, J. A. K. Howard, J. N. Nicholls, J. L. Spencer and D. G. Turner, *J. Chem. Soc.*, *Chem. Commun.*, 1980, 572.

(28) R. R. Schrock, K. Y. Shih, D. A. Dobbs and W. M. Davis, *J. Am. Chem. Soc.*, 1995, **117**, 6609.

(29) D. A. Dobbs, R. R. Schrock and W. M. Davis, *Inorg. Chim. Acta*, 1997, **263**, 171.

(30) C. C. Pye and T. Ziegler, *Theor. Chem. Acc.*, 1999, **101**, 396.

(31) A. Klamt and G. Schuurmann, *J. Chem. Soc.*, *Perkin Trans. 2*, 1993, 799.

(32) J. F. Odzak, A. M. Argo, F. S. Lai, B. C. Gates, K. Pandya and L. Feraria, *Rev. Sci. Instrum.*, 2001, **72**, 3943.

(33) M. Newville, B. Ravel, D. Haskel, J. J. Rehr, E. A. Stern and Y. Yacoby, *Physica B*, 1995, **208**, 154.

(34) M. J. Newville, *Synchrotron Radiat.*, 2001, **8**, 96.

(35) S. I. Zabinsky, J. J. Rehr, A. Ankudinov, R. C. Albers and M. J. Eller, *Phys. Rev. B*, 1995, **52**, 2995.

(36) V. Pfennig and K. Seppelt, *Science*, 1996, **271**, 626.

(37) P. Villars, L. D. Calvert and W. B. Pearson, Pearson’s Handbook of Crystallographic Data for Intermetallic Phases, American Society for Metals, Metals Park, 1985, pp. 2946-3198.

(38) P. S. Kirlin, F. B. M. van Zon, D. C. Koningsberger and B. C. Gates, *J. Phys. Chem.*,-Us 1990, **94**, 8439.

(39) J. B. A. D. van Zon,; D. C. Koningsberger, H. F. J. van't Blik and D. E. Sayers, *J. Chem. Phys.*, 1985, **82**, 5742.

(40) (a) M. V. Luzgin, V. A. Rogov, S. S. Arzumanov, A. V. Toktarev, A. G. Stepanov and V. N. Parmon, Angew. Chem. Int. Ed., 2008, 47, 4559; (b) J. F. Wu, W. D. Wang, J. Xu, F. Deng and W. Wang, Chem. – Eur. J., 2010, 16, 14016; (c) U. Patil, Y. Saih, E. Abou-Hamad, A. Hamieh, J. D. A. Pelletier and J. M. Basset, Chem. Commun., 2014, 50, 12348; (d) A. Hamieh, Y. Chen, S. Abdel-Azeim, E. Abou-hamad, S. Goh, M. Samantaray, R. Dey, L. Cavallo and J. M. Basset, ACS Catal., 2015, 5, 2164.

(41) D. Gregson, J. A. K. Howard, J. N. Nicholls, J. L. Spencer and D. G. Turner, *J. Chem. Soc., Chem. Comm.*, **1980**, 572.

(42) R.R. Schrock, K.-Y. Shih, D. A. Dobbs, W. M. Davis, *J. Am. Chem. Soc.* 1995, **117**, 6609.

(43) D. A. Dobbs, R. R. Schrock, W. M. Davis, *Inorg. Chim. Acta*, 1997, **263**, 171.
